# Supplementary material for: Evolutionary adaptation of bacterial proteomes to translation-impeding sequences
Source: EMBO J. 2025 Dec 9;45(6):1957–79. doi: 10.1038/s44318-025-00651-6 (PMC12992588; doi:10.1038/s44318-025-00651-6)
Supplement: Supplementary file 1 — Appendix [file 44318_2025_651_MOESM1_ESM.pdf]

Appendix for

Evolutionary adaptation of bacterial proteomes to translation-impeding sequences

Table of contents

Item	Page
Appendix Fig S1: Amino acid frequencies across bacterial phyla.	1
Appendix Fig S2: Small proteins with RGPP or RAPP motifs are encoded upstream of genes with diverse functions.	2
Appendix Fig S3: RGPP and RAPP are enriched in the C-termini of proteins encoded upstream of various gene groups.	3
Appendix Fig S4: Small proteins containing RGPP or RAPP motifs, encoded upstream of genes with diverse functions, tend to possess a localization signal.	4
Appendix Fig S5: Function of downstream genes.	5
Appendix Fig S6: Conserved motifs in *S. lividans* arrest peptides	6
Appendix Fig S7: Predicted structures of *S. lividans* RAPP/RGPP-containing proteins	7
Appendix Table S1: *B. subtilis* strains	8
Appendix Table S2: *E. coli* strains	12
Appendix Table S3: Plasmids	14
Appendix Table S4: Primers	16
Appendix Table S5: Templates for PURE system	18

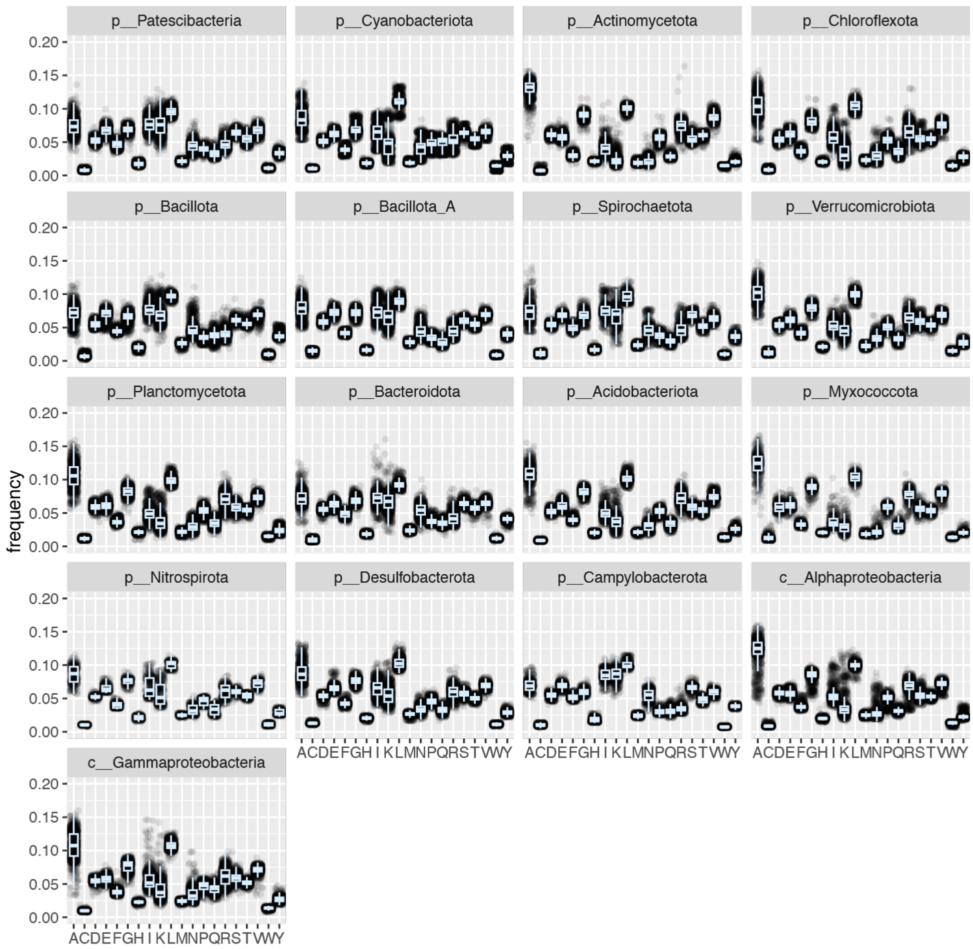

Appendix Fig. S1. Amino acid frequencies across bacterial phyla. Dot plots and box plots shows the diversity of amino acid frequencies of phyla Patescibacteria (n = 1000), Cyanobacteriota (n = 944), Actinomycetota (n = 1000), Chloroflexota (n = 1000), Bacillota (n = 1000), Bacillota_A (n = 1000), Spirochaetota (n = 606), Verrucomicrobiota (n = 865), Planctomycetota (n = 1000), Bacteroidota (n = 1000), Acidobacteriota (n = 758), Myxococcota (n = 425), Nitrospirota (n = 300), Desulfobacterota (n = 836) and Campylobacterota (n = 501), and classes Alphaproteobacteria (n = 1000) and Gammaproteobacteria (n = 1000).

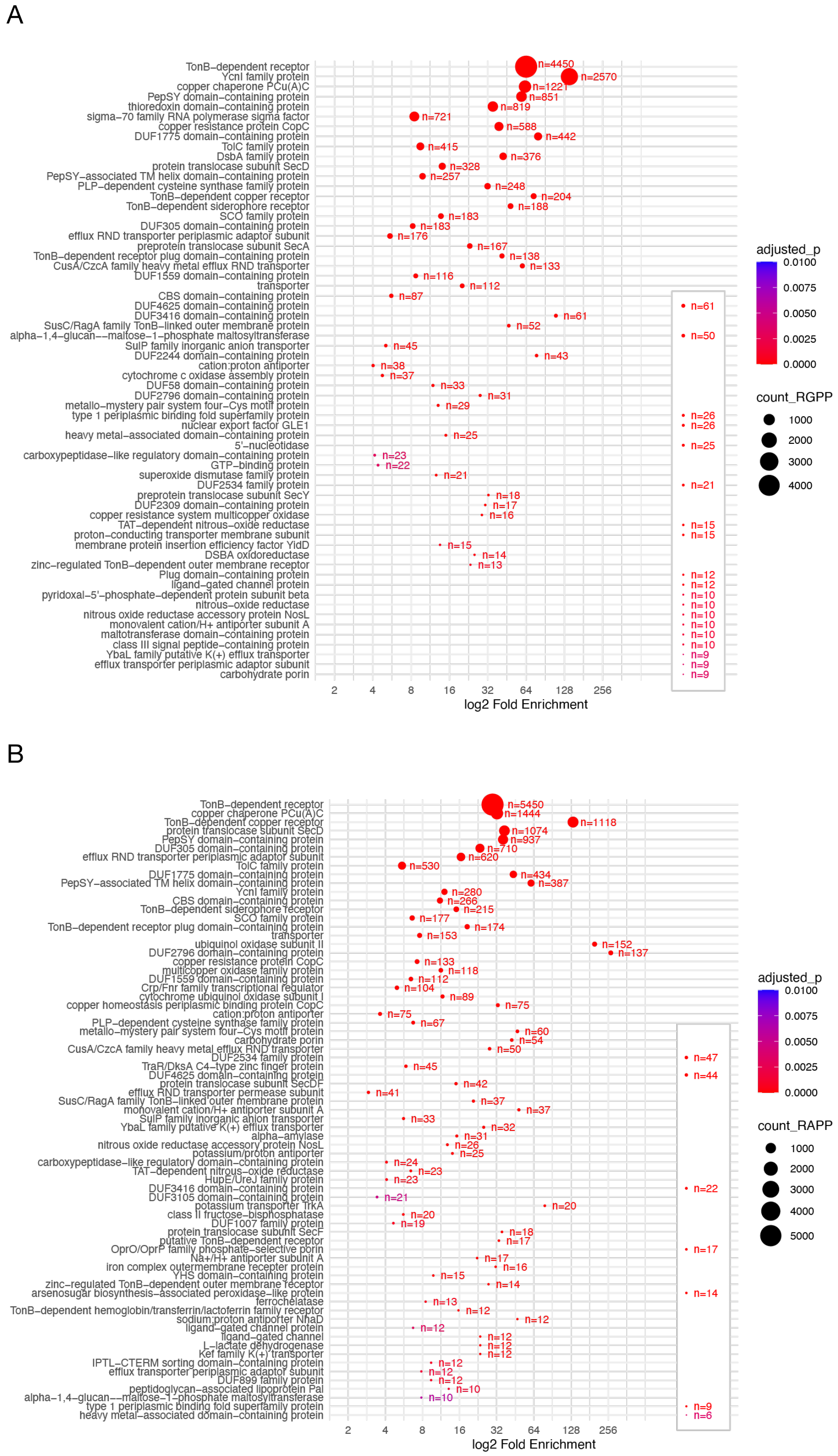

Appendix Fig. S2. Small proteins with RGPP or RAPP motifs are encoded upstream of genes with diverse functions. (A, B) Gene groups with a higher proportion of small uORFs encoding RGPP compared to AGPP (A), or RAPP compared to AAPP (Bb), were extracted. The horizontal axis represents fold enrichment (RGPP/AGPP or RAPP/AAPP), while the size of the circles corresponds to the number of genes in each group. Circle color indicates the p-value (Fisher's exact test) after Benjamini–Hochberg adjustment. Groups with an infinite fold enrichment score due to the absence of the AAPP or AGPP motif are enclosed in squares.

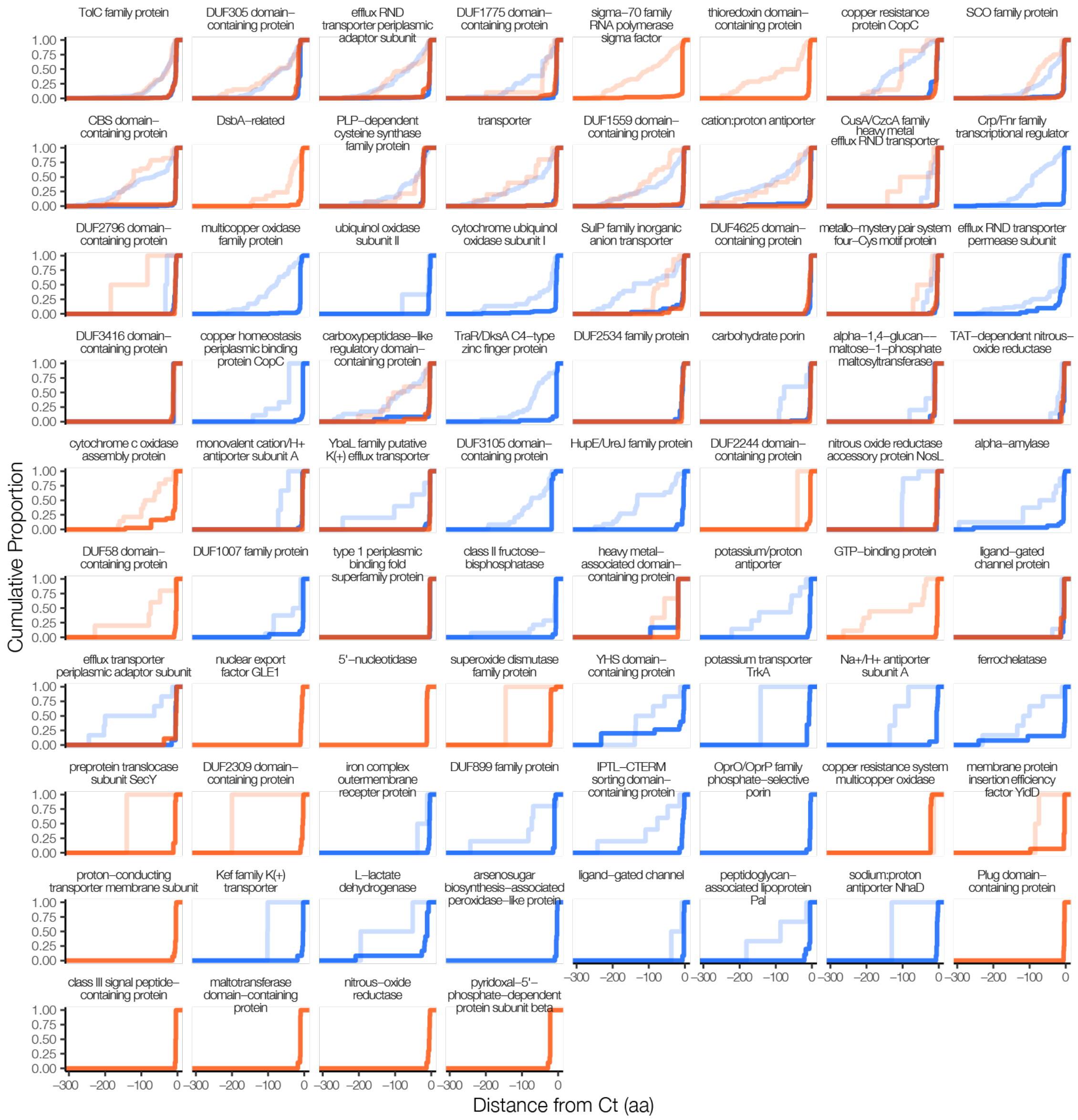

Appendix Fig. S3. RGPP and RAPP are enriched in the C-termini of proteins encoded upstream of various gene groups. CDF plots show the distances between the C-terminus and RGPP (red), RAPP (blue), AGPP (pale red), or AAPP (pale blue) motifs. uORFs are categorized based on the groups of their downstream genes.

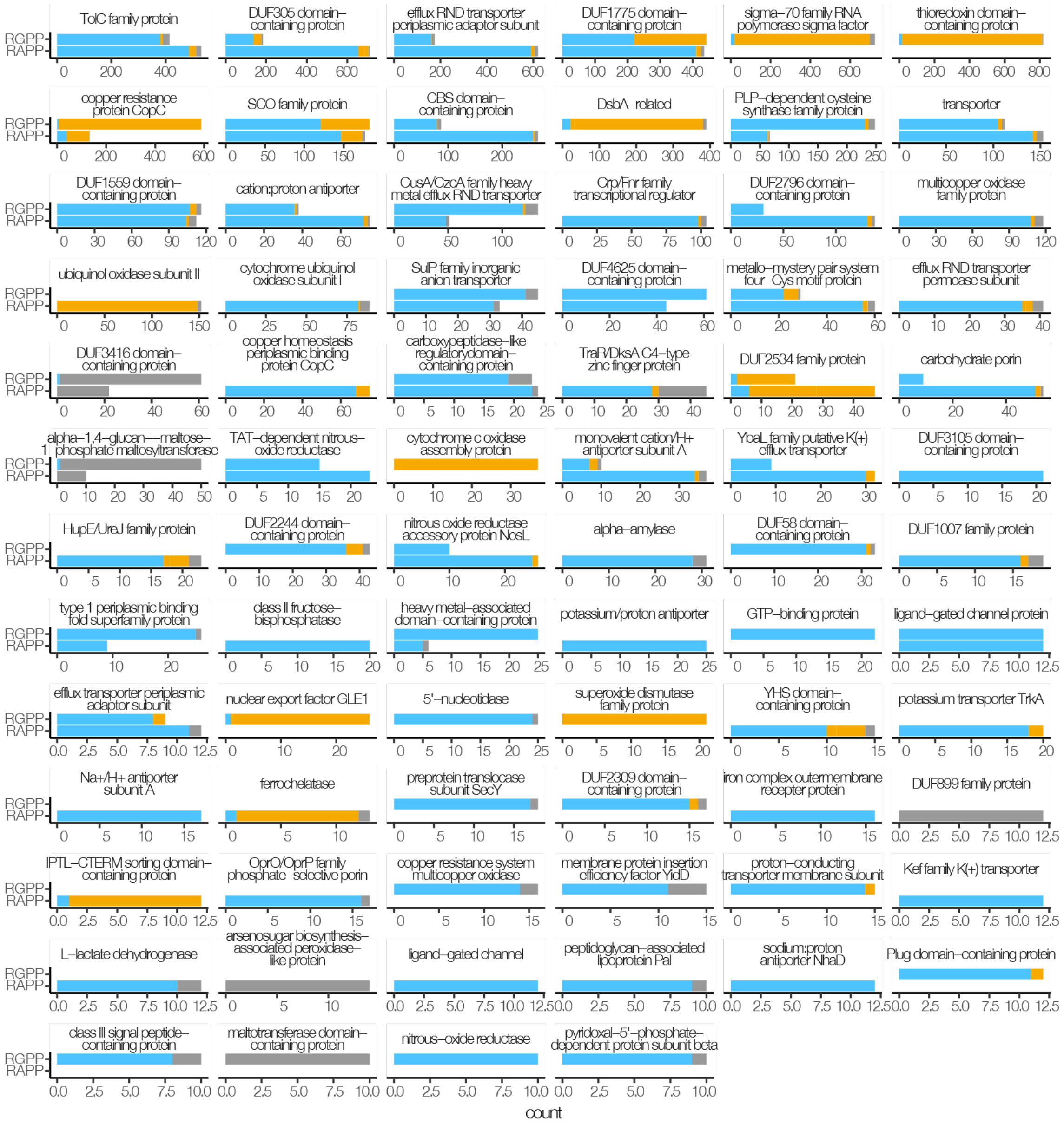

Appendix Fig. S4. Small proteins containing RGPP or RAPP motifs, encoded upstream of genes with diverse functions, tend to possess a localization signal. Stacked bar plots show the numbers of secretory proteins (SP: light blue), transmembrane proteins (TM: light orange), and cytosolic proteins (CP: grey) containing RGPP/RAPP motifs, categorized by downstream gene groups.

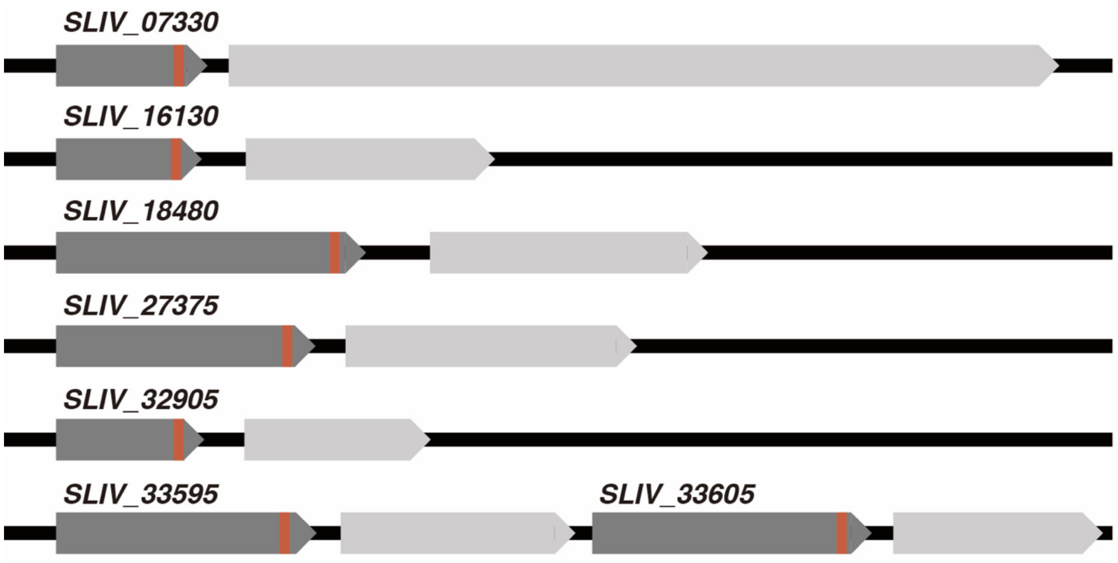

locus tag	downstream gene
SLIV_07330	SecDF
SLIV_16130	CopM
SLIV_18480	YcnI
SLIV_27375	thioredoxin like protein
SLIV_32905	short-chain dehydrogenase/reductase
SLIV_33595	ECF σ
SLIV_33605	ECF σ

Appendix Fig. S5. Function of downstream genes. Schematic representation of gene context and function of downstream genes of *S. lividans* arrest peptides.

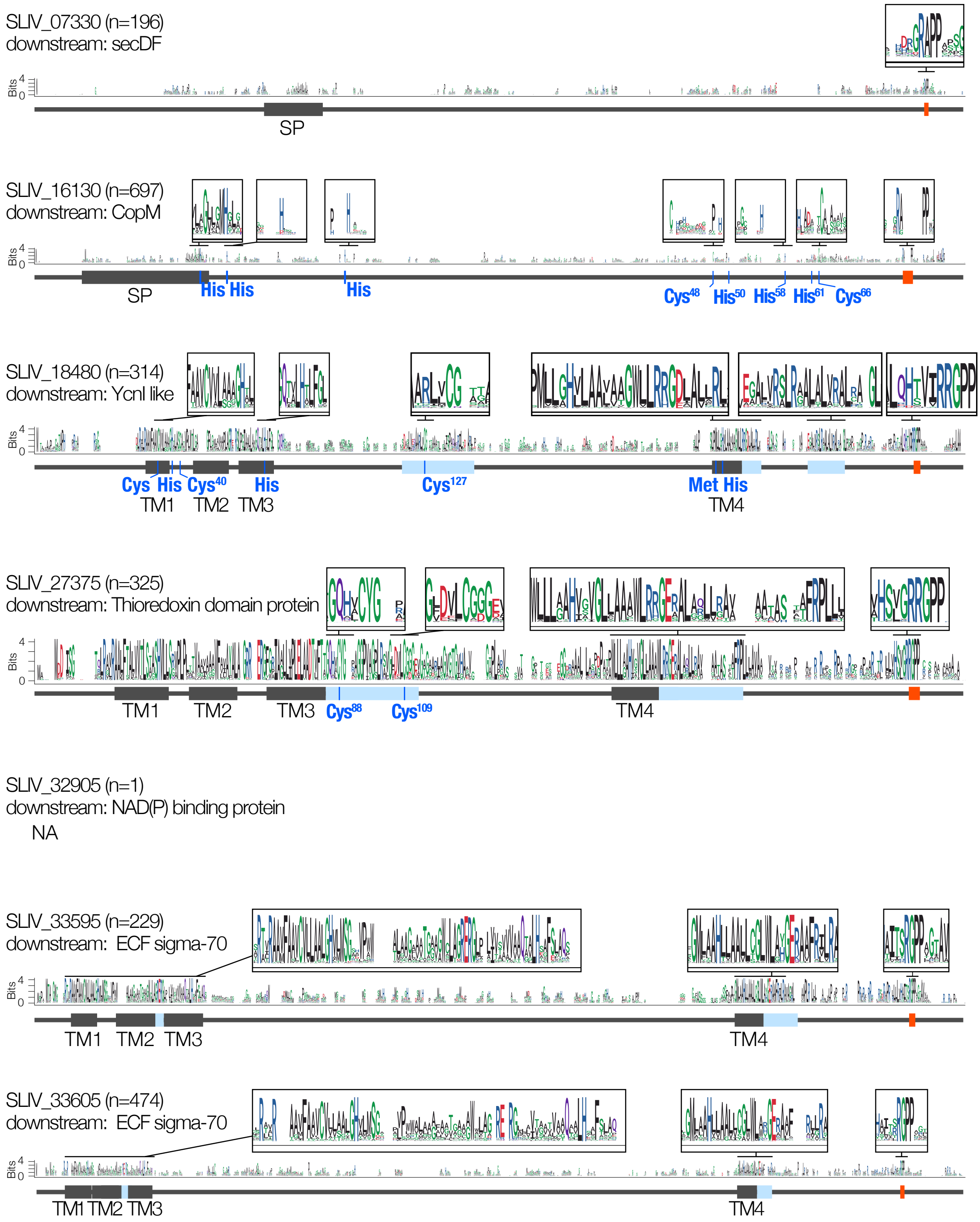

Appendix Fig. S6. Conserved motifs in *S. lividans* arrest peptides. Sequence logos illustrating the N-terminal conservation of protein homologues containing the RGPP or RAPP motifs in *Streptomyces lividans* are shown. Several highly conserved regions are shown in enlarged inset. Protein domains are depicted below each sequence logo. Signal peptides (SP) and transmembrane segments (TM) are indicated with grey boxes, relatively conserved regions with light blue boxes, and RAPP or RGPP motifs with orange-red boxes. Conserved cysteine, histidine, and methionine residues are marked in blue. Residue numbers correspond to the *S. lividans* homologue. Homologues of SLIV_32905 were not detected in the clusters analyzed (see Methods).

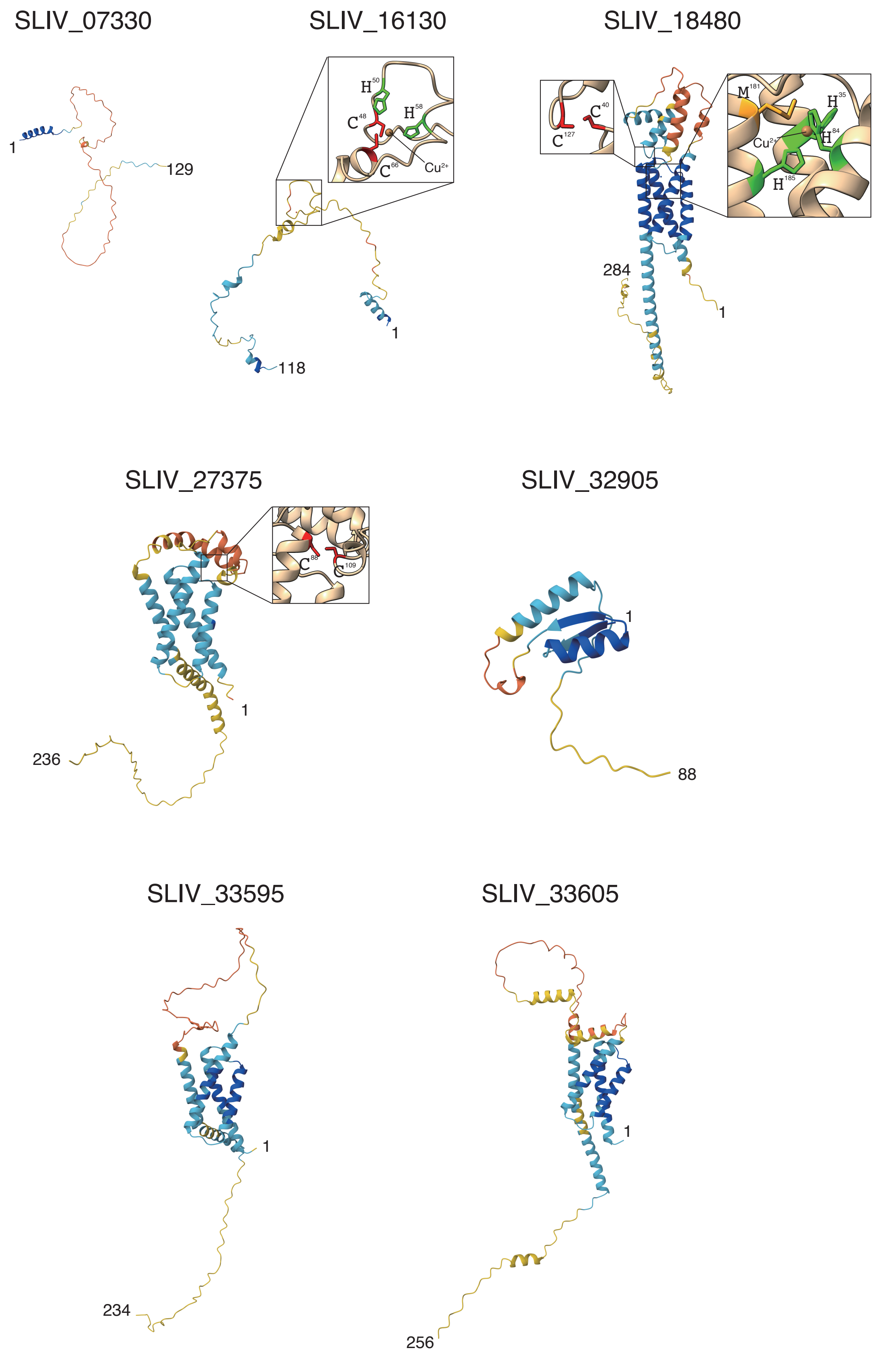

Appendix Fig. S7. Predicted structures of *S. lividans* RAPP/RGPP-containing proteins. The three-dimensional structures of each protein were predicted using AlphaFold3. For SLIV_16130 and SLIV_18480, structure predictions were performed with Cu^{2+} ion included. The predicted structures were colored according to the pLDDT scores: blue for regions with scores >90, lightblue for 70–90, yellow for 70–50, and orange for <50.

Appendix Table S1: *B. subtilis* strains

strains	genotype	parent	plasmid	ref
PY79	*wildtype*			(1)
SCB2619	*rplWΩkan*			(2)
SCB2942	*rplD(d66-70)Ωkan*			(2)
SCB3958	*amyE::PmifM gfp-apcA(62-108)-flag-lacZΩcat*	PY79	pCH2124	(3)
SCB3959	*amyE::PmifM gfp-apdA(39-128)-flag-lacZΩcat*	PY79	pCH2125	(3)
SCB3999	*amyE::PmifM gfp-apdA(39-128)(R120A)-flag-lacZΩcat*	PY79	pCH2134	(3)
SCB4351	*rplWΩkan, amyE::PmifM gfp-apcA(62-108)-myc-lacZΩcat*	SCB2619	pCH2316	(4)
SCB4358	*rplW(d65-69)Ωkan, amyE::PmifM gfp-apcA(62-108)-myc-lacZΩcat*	SCB2634	pCH2316	(4)
SCB4365	*rplD(d66-70)Ωkan, amyE::PmifM gfp-apcA(62-108)-myc-lacZΩcat*	SCB2942	pCH2316	(4)
SCB4619	*amyE::PmifM gfp-apcA(62-108)-R102A/G105P(AAPP)-myc-lacZΩcat*	PY79	pCH2600	*
SCB4622	*rplWΩkan, amyE::PmifM gfp-apcA(62-108)-myc-lacZΩcat*	SCB2619	pCH2425	*
SCB4623	*rplWΩkan, amyE::PmifM gfp-apcA(62-108)-R102A-myc-lacZΩcat*	SCB2619	pCH2426	*
SCB4624	*rplWΩkan, amyE::PmifM gfp-apcA(62-108)-G105P-myc-lacZΩcat*	SCB2619	pCH2437	*
SCB4625	*rplWΩkan, amyE::PmifM gfp-apcA(62-108)-A103G/G105P(RGPP)-myc-lacZΩcat*	SCB2619	pCH2599	*
SCB4626	*rplWΩkan, amyE::PmifM gfp-apcA(62-108)-P104G/G105P(RAGP)-myc-lacZΩcat*	SCB2619	pCH2601	*
SCB4627	*rplD(d66-70)Ωkan, amyE::PmifM gfp-apcA(62-108)-myc-lacZΩcat*	SCB2942	pCH2425	*
SCB4628	*rplD(d66-70)Ωkan, amyE::PmifM gfp-apcA(62-108)-R102A-myc-lacZΩcat*	SCB2942	pCH2426	*
SCB4629	*rplD(d66-70)Ωkan, amyE::PmifM gfp-apcA(62-108)-G105P-myc-lacZΩcat*	SCB2942	pCH2437	*
SCB4630	*rplD(d66-70)Ωkan, amyE::PmifM gfp-apcA(62-108)-A103G/G105P(RGPP)-myc-lacZΩcat*	SCB2942	pCH2599	*
SCB4631	*rplD(d66-70)Ωkan, amyE::PmifM gfp-apcA(62-108)-P104G/G105P(RAGP)-myc-lacZΩcat*	SCB2942	pCH2601	*
SCB4632	*amyE::PmifM gfp-Sm_ApdP(34-140)-myc-lacZΩcat*	PY79	pCH2429	*
SCB4633	*amyE::PmifM gfp-Sm_ApdP(34-140)-R131A-myc-lacZΩcat*	PY79	pCH2430	*
SCB4634	*amyE::PmifM gfp-Sm_ApdP(34-140)-A132G-myc-lacZΩcat*	PY79	pKIG1441	*
SCB4635	*amyE::PmifM gfp-Sm_ApdP(34-140)-P134G-myc-lacZΩcat*	PY79	pKIG1439	*
SCB4636	*amyE::PmifM gfp-Sm_ApdP(34-140)-P133G-myc-lacZΩcat*	PY79	pKIG1435	*
SCB4637	*amyE::PmifM gfp-Sm_ApdP(34-140)-R131A/P133G-myc-lacZΩcat*	PY79	pKIG1443	*
SKB52	*amyE::PmifM gfp-apcA(62-108)-R102A-flag-lacZΩcat*	PY79	pSK63	*
SKB53	*amyE::PmifM gfp-apcA(62-108)-A103S-flag-lacZΩcat*	PY79	pSK64	*
SKB54	*amyE::PmifM gfp-apcA(62-108)-P104A-flag-lacZΩcat*	PY79	pSK66	*
SKB71	*amyE::PmifM gfp-apdA(39-128)-P122A-flag-lacZΩcat*	PY79	pSK88	*
SKB73	*amyE::PmifM gfp-apdA(39-128)-A121S-flag-lacZΩcat*	PY79	pSK87	*
SKB79	*amyE::PmifM gfp-apdP(34-140)-flag-lacZΩcat*	PY79	pSK92	*
SKB87	*amyE::PmifM gfp-apdP(34-140)-R131A-flag-lacZΩcat*	PY79	pSK107	*
SKB88	*amyE::PmifM gfp-apdP(34-140)-A132S-flag-lacZΩcat*	PY79	pSK106	*
SKB89	*amyE::PmifM gfp-apdP(34-140)-P133A-flag-lacZΩcat*	PY79	pSK101	*
SKB90	*amyE::PmifM gfp-apcA(62-108)-P104G-flag-lacZΩcat*	PY79	pSK111	*
SKB91	*amyE::PmifM gfp-apdA(39-128)-P122G-flag-lacZΩcat*	PY79	pSK112	*
SKB92	*amyE::PmifM gfp-apdP(34-140)-P133G-flag-lacZΩcat*	PY79	pSK113	*
SKB93	*amyE::PmifM gfp-apcA(62-108)-P104T-flag-lacZΩcat*	PY79	pSK155	*
SKB94	*amyE::PmifM gfp-apcA(62-108)-P104F-flag-lacZΩcat*	PY79	pSK157	*
SKB95	*amyE::PmifM gfp-apcA(62-108)-P104Q-flag-lacZΩcat*	PY79	pSK156	*
SKB96	*amyE::PmifM gfp-apcA(62-108)-P104R-flag-lacZΩcat*	PY79	pSK122	*
SKB97	*amyE::PmifM gfp-apcA(62-108)-P104K-flag-lacZΩcat*	PY79	pSK139	*
SKB98	*amyE::PmifM gfp-apcA(62-108)-P104C-flag-lacZΩcat*	PY79	pSK158	*
SKB99	*amyE::PmifM gfp-apcA(62-108)-P104S-flag-lacZΩcat*	PY79	pSK138	*
SKB100	*amyE::PmifM gfp-apcA(62-108)-P104Y-flag-lacZΩcat*	PY79	pSk123	*
SKB102	*amyE::PmifM gfp-apcA(62-108)-A103W-flag-lacZΩcat*	PY79	pSK152	*
SKB103	*amyE::PmifM gfp-apcA(62-108)-A103I-flag-lacZΩcat*	PY79	pSK151	*
SKB104	*amyE::PmifM gfp-apcA(62-108)-A103C-flag-lacZΩcat*	PY79	pSK150	*
SKB106	*amyE::PmifM gfp-apcA(62-108)-A103M-flag-lacZΩcat*	PY79	pSK159	*
SKB107	*amyE::PmifM gfp-apcA(62-108)-A103N-flag-lacZΩcat*	PY79	pSK161	*
SKB108	*amyE::PmifM gfp-apcA(62-108)-A103P-flag-lacZΩcat*	PY79	pSK154	*
SKB109	*amyE::PmifM gfp-apcA(62-108)-A103R-flag-lacZΩcat*	PY79	pSK153	*

SKB110	*amyE::PmifM gfp-apcA(62-108)-A103H-flag-lacZΩ cat*	PY79	pSK148	*
SKB111	*amyE::PmifM gfp-apcA(62-108)-A103V-flag-lacZΩ cat*	PY79	pSK143	*
SKB112	*amyE::PmifM gfp-apcA(62-108)-A103G-flag-lacZΩ cat*	PY79	pSK142	*
SKB115	*amyE::PmifM gfp-apcA(62-108)-A103E-flag-lacZΩ cat*	PY79	pSK140	*
SKB116	*amyE::PmifM gfp-apcA(62-108)-A103L-flag-lacZΩ cat*	PY79	pSK149	*
SKB117	*amyE::PmifM gfp-apcA(62-108)-A103Q-flag-lacZΩ cat*	PY79	pSK166	*
SKB118	*amyE::PmifM gfp-apcA(62-108)-A103K-flag-lacZΩ cat*	PY79	pSK160	*
SKB119	*amyE::PmifM gfp-apcA(62-108)-A103Y-flag-lacZΩ cat*	PY79	pSK170	*
SKB120	*amyE::PmifM gfp-apcA(62-108)-A103D-flag-lacZΩ cat*	PY79	pSK169	*
SKB121	*amyE::PmifM gfp-apcA(62-108)-P104L-flag-lacZΩ cat*	PY79	pSK168	*
SKB122	*amyE::PmifM gfp-apcA(62-108)-P104N-flag-lacZΩ cat*	PY79	pSK167	*
SKB123	*amyE::PmifM gfp-apcA(62-108)-R102C-flag-lacZΩ cat*	PY79	pSK164	*
SKB124	*amyE::PmifM gfp-apcA(62-108)-R102D-flag-lacZΩ cat*	PY79	pSK127	*
SKB125	*amyE::PmifM gfp-apcA(62-108)-R102E-flag-lacZΩ cat*	PY79	pSK182	*
SKB127	*amyE::PmifM gfp-apcA(62-108)-R102F-flag-lacZΩ cat*	PY79	pSK162	*
SKB128	*amyE::PmifM gfp-apcA(62-108)-R102M-flag-lacZΩ cat*	PY79	pSK165	*
SKB130	*amyE::PmifM gfp-apcA(62-108)-R102I-flag-lacZΩ cat*	PY79	pSK128	*
SKB131	*amyE::PmifM gfp-apcA(62-108)-R102K-flag-lacZΩ cat*	PY79	pSK163	*
SKB132	*amyE::PmifM gfp-apcA(62-108)-R102P-flag-lacZΩ cat*	PY79	pSK137	*
SKB133	*amyE::PmifM gfp-apcA(62-108)-R102S-flag-lacZΩ cat*	PY79	pSK126	*
SKB135	*amyE::PmifM gfp-apcA(62-108)-R102V-flag-lacZΩ cat*	PY79	pSK175	*
SKB137	*amyE::PmifM gfp-apcA(62-108)-R102W-flag-lacZΩ cat*	PY79	pSK136	*
SKB138	*amyE::PmifM gfp-apcA(62-108)-R102Y-flag-lacZΩ cat*	PY79	pSK181	*
SKB140	*amyE::PmifM gfp-apcA(62-108)-A103F-flag-lacZΩ cat*	PY79	pSK177	*
SKB142	*amyE::PmifM gfp-apcA(62-108)-A103T-flag-lacZΩ cat*	PY79	pSK176	*
SKB144	*amyE::PmifM gfp-apcA(62-108)-P104D-flag-lacZΩ cat*	PY79	pSK173	*
SKB145	*amyE::PmifM gfp-apcA(62-108)-P104E-flag-lacZΩ cat*	PY79	pSK178	*
SKB147	*amyE::PmifM gfp-apcA(62-108)-P104I-flag-lacZΩ cat*	PY79	pSK171	*
SKB149	*amyE::PmifM gfp-apcA(62-108)-P104M-flag-lacZΩ cat*	PY79	pSK172	*
SKB151	*amyE::PmifM gfp-apcA(62-108)-P104V-flag-lacZΩ cat*	PY79	pSK174	*
SKB153	*amyE::PmifM gfp-apcA(62-108)-P104W-flag-lacZΩ cat*	PY79	pSK179	*
SKB154	*amyE::PmifM gfp-apcA(62-108)-R102N-flag-lacZΩ cat*	PY79	pSK190	*
SKB155	*amyE::PmifM gfp-apcA(62-108)-R102Q-flag-lacZΩ cat*	PY79	pSK189	*
SKB156	*amyE::PmifM gfp-apcA(62-108)-R102L-flag-lacZΩ cat*	PY79	pSK188	*
SKB157	*amyE::PmifM gfp-apcA(62-108)-R102H-flag-lacZΩ cat*	PY79	pSK187	*
SKB158	*amyE::PmifM gfp-apcA(62-108)-R102G-flag-lacZΩ cat*	PY79	pSK186	*
SKB160	*amyE::PmifM gfp-apcA(62-108)-R102T-flag-lacZΩ cat*	PY79	pSK184	*
SKB161	*amyE::PmifM gfp-apcA(62-108)-P104H-flag-lacZΩ cat*	PY79	pSK183	*
SKB162	*amyE::PmifM gfp-apdA(39-128)-R120C-flag-lacZΩ cat*	PY79	pSK201	*
SKB163	*amyE::PmifM gfp-apdA(39-128)-R120E-flag-lacZΩ cat*	PY79	pSK195	*
SKB164	*amyE::PmifM gfp-apdA(39-128)-R120G-flag-lacZΩ cat*	PY79	pSK196	*
SKB165	*amyE::PmifM gfp-apdA(39-128)-R120K-flag-lacZΩ cat*	PY79	pSK193	*
SKB166	*amyE::PmifM gfp-apdA(39-128)-R120L-flag-lacZΩ cat*	PY79	pSK194	*
SKB167	*amyE::PmifM gfp-apdA(39-128)-R120M-flag-lacZΩ cat*	PY79	pSK192	*
SKB168	*amyE::PmifM gfp-apdA(39-128)-R120P-flag-lacZΩ cat*	PY79	pSK198	*
SKB169	*amyE::PmifM gfp-apdA(39-128)-R120V-flag-lacZΩ cat*	PY79	pSK199	*
SKB170	*amyE::PmifM gfp-apdA(39-128)-R120W-flag-lacZΩ cat*	PY79	pSK200	*
SKB171	*amyE::PmifM gfp-apdA(39-128)-R120Y-flag-lacZΩ cat*	PY79	pSK197	*
SKB173	*amyE::PmifM gfp-apdA(39-128)-R120Q-flag-lacZΩ cat*	PY79	pSK202	*
SKB174	*amyE::PmifM gfp-apdA(39-128)-R120T-flag-lacZΩ cat*	PY79	pSK203	*
SKB175	*amyE::PmifM gfp-apdA(39-128)-A121C-flag-lacZΩ cat*	PY79	pSK210	*
SKB176	*amyE::PmifM gfp-apdA(39-128)-A121D-flag-lacZΩ cat*	PY79	pSK207	*
SKB177	*amyE::PmifM gfp-apdA(39-128)-A121E-flag-lacZΩ cat*	PY79	pSK209	*
SKB178	*amyE::PmifM gfp-apdA(39-128)-A121K-flag-lacZΩ cat*	PY79	pSK204	*
SKB179	*amyE::PmifM gfp-apdA(39-128)-A121L-flag-lacZΩ cat*	PY79	pSK206	*

SKB180	*amyE::PmifM gfp-apdA(39-128)-A121R-flag-lacZΩ cat*	PY79	pSK205	*
SKB181	*amyE::PmifM gfp-apdA(39-128)-A121T-flag-lacZΩ cat*	PY79	pSK211	*
SKB182	*amyE::PmifM gfp-apdA(39-128)-A121V-flag-lacZΩ cat*	PY79	pSK208	*
SKB183	*amyE::PmifM gfp-apdA(39-128)-R120D-flag-lacZΩ cat*	PY79	pSK212	*
SKB184	*amyE::PmifM gfp-apdA(39-128)-R120F-flag-lacZΩ cat*	PY79	pSK213	*
SKB185	*amyE::PmifM gfp-apdA(39-128)-R120H-flag-lacZΩ cat*	PY79	pSK214	*
SKB186	*amyE::PmifM gfp-apdA(39-128)-R120I-flag-lacZΩ cat*	PY79	pSK216	*
SKB187	*amyE::PmifM gfp-apdA(39-128)-R120S-flag-lacZΩ cat*	PY79	pSK215	*
SKB188	*amyE::PmifM gfp-apdA(39-128)-P122D-flag-lacZΩ cat*	PY79	pSK218	*
SKB189	*amyE::PmifM gfp-apdA(39-128)-P122E-flag-lacZΩ cat*	PY79	pSK219	*
SKB190	*amyE::PmifM gfp-apdA(39-128)-P122F-flag-lacZΩ cat*	PY79	pSK221	*
SKB191	*amyE::PmifM gfp-apdA(39-128)-P122L-flag-lacZΩ cat*	PY79	pSK217	*
SKB192	*amyE::PmifM gfp-apdA(39-128)-P122M-flag-lacZΩ cat*	PY79	pSK225	*
SKB193	*amyE::PmifM gfp-apdA(39-128)-P122Q-flag-lacZΩ cat*	PY79	pSK223	*
SKB194	*amyE::PmifM gfp-apdA(39-128)-P122R-flag-lacZΩ cat*	PY79	pSK224	*
SKB195	*amyE::PmifM gfp-apdA(39-128)-P122S-flag-lacZΩ cat*	PY79	pSK220	*
SKB196	*amyE::PmifM gfp-apdA(39-128)-P122V-flag-lacZΩ cat*	PY79	pSK222	*
SKB197	*amyE::PmifM gfp-apdA(39-128)-P122W-flag-lacZΩ cat*	PY79	pSK226	*
SKB199	*amyE::PmifM gfp-apdA(39-128)-A121F-flag-lacZΩ cat*	PY79	pSK232	*
SKB200	*amyE::PmifM gfp-apdA(39-128)-A121G-flag-lacZΩ cat*	PY79	pSK234	*
SKB201	*amyE::PmifM gfp-apdA(39-128)-A121H-flag-lacZΩ cat*	PY79	pSK230	*
SKB202	*amyE::PmifM gfp-apdA(39-128)-A121I-flag-lacZΩ cat*	PY79	pSK236	*
SKB203	*amyE::PmifM gfp-apdA(39-128)-A121M-flag-lacZΩ cat*	PY79	pSK228	*
SKB204	*amyE::PmifM gfp-apdA(39-128)-A121N-flag-lacZΩ cat*	PY79	pSK235	*
SKB205	*amyE::PmifM gfp-apdA(39-128)-A121P-flag-lacZΩ cat*	PY79	pSK231	*
SKB206	*amyE::PmifM gfp-apdA(39-128)-A121Q-flag-lacZΩ cat*	PY79	pSK229	*
SKB207	*amyE::PmifM gfp-apdA(39-128)-A121W-flag-lacZΩ cat*	PY79	pSK233	*
SKB208	*amyE::PmifM gfp-apdA(39-128)-A121Y-flag-lacZΩ cat*	PY79	pSK237	*
SKB209	*amyE::PmifM gfp-apdA(39-128)-P122N-flag-lacZΩ cat*	PY79	pSK238	*
SKB211	*amyE::PmifM gfp-apdA(39-128)-P122T-flag-lacZΩ cat*	PY79	pSK239	*
SKB213	*amyE::PmifM gfp-apdA(39-128)-R120N-flag-lacZΩ cat*	PY79	pSK241	*
SKB214	*amyE::PmifM gfp-apdA(39-128)-P122C-flag-lacZΩ cat*	PY79	pSK242	*
SKB215	*amyE::PmifM gfp-apdA(39-128)-P122H-flag-lacZΩ cat*	PY79	pSK244	*
SKB216	*amyE::PmifM gfp-apdA(39-128)-P122Y-flag-lacZΩ cat*	PY79	pSK243	*
SKB217	*amyE::PmifM gfp-apdA(39-128)-P122K-flag-lacZΩ cat*	PY79	pSK245	*
SKB218	*amyE::PmifM gfp-apdA(39-128)-P122I-flag-lacZΩ cat*	PY79	pSK246	*
SKB219	*amyE::PmifM gfp-apdP(34-140)-R131D-flag-lacZΩ cat*	PY79	pSK254	*
SKB220	*amyE::PmifM gfp-apdP(34-140)-R131E-flag-lacZΩ cat*	PY79	pSK251	*
SKB221	*amyE::PmifM gfp-apdP(34-140)-R131G-flag-lacZΩ cat*	PY79	pSK249	*
SKB222	*amyE::PmifM gfp-apdP(34-140)-R131I-flag-lacZΩ cat*	PY79	pSK250	*
SKB223	*amyE::PmifM gfp-apdP(34-140)-R131K-flag-lacZΩ cat*	PY79	pSK252	*
SKB224	*amyE::PmifM gfp-apdP(34-140)-R131L-flag-lacZΩ cat*	PY79	pSK256	*
SKB225	*amyE::PmifM gfp-apdP(34-140)-R131M-flag-lacZΩ cat*	PY79	pSK247	*
SKB226	*amyE::PmifM gfp-apdP(34-140)-R131S-flag-lacZΩ cat*	PY79	pSK255	*
SKB227	*amyE::PmifM gfp-apdP(34-140)-R131V-flag-lacZΩ cat*	PY79	pSK248	*
SKB228	*amyE::PmifM gfp-apdP(34-140)-R131W-flag-lacZΩ cat*	PY79	pSK253	*
SKB229	*amyE::PmifM gfp-apdP(34-140)-A132G-flag-lacZΩ cat*	PY79	pSK258	*
SKB230	*amyE::PmifM gfp-apdP(34-140)-A132V-flag-lacZΩ cat*	PY79	pSK257	*
SKB231	*amyE::PmifM gfp-apdP(34-140)-A132W-flag-lacZΩ cat*	PY79	pSK259	*
SKB232	*amyE::PmifM gfp-apdP(34-140)-R131C-flag-lacZΩ cat*	PY79	pSK265	*
SKB233	*amyE::PmifM gfp-apdP(34-140)-R131H-flag-lacZΩ cat*	PY79	pSK268	*
SKB234	*amyE::PmifM gfp-apdP(34-140)-R131P-flag-lacZΩ cat*	PY79	pSK269	*
SKB235	*amyE::PmifM gfp-apdP(34-140)-A132K-flag-lacZΩ cat*	PY79	pSK270	*
SKB236	*amyE::PmifM gfp-apdP(34-140)-P133H-flag-lacZΩ cat*	PY79	pSK272	*
SKB237	*amyE::PmifM gfp-apdP(34-140)-P133I-flag-lacZΩ cat*	PY79	pSK261	*

SKB238	*amyE::PmifM gfp-apdP(34-140)-P133L-flag-lacZΩ cat*	PY79	pSK273	*
SKB239	*amyE::PmifM gfp-apdP(34-140)-P133M-flag-lacZΩ cat*	PY79	pSK260	*
SKB240	*amyE::PmifM gfp-apdP(34-140)-P133S-flag-lacZΩ cat*	PY79	pSK264	*
SKB241	*amyE::PmifM gfp-apdP(34-140)-P133V-flag-lacZΩ cat*	PY79	pSK271	*
SKB242	*amyE::PmifM gfp-apdP(34-140)-P133Y-flag-lacZΩ cat*	PY79	pSK262	*
SKB246	*amyE::PmifM gfp-apdP(34-140)-R131N-flag-lacZΩ cat*	PY79	pSK266	*
SKB247	*amyE::PmifM gfp-apdP(34-140)-R131Q-flag-lacZΩ cat*	PY79	pSK274	*
SKB248	*amyE::PmifM gfp-apdP(34-140)-R131Y-flag-lacZΩ cat*	PY79	pSK267	*
SKB249	*amyE::PmifM gfp-apdP(34-140)-A132D-flag-lacZΩ cat*	PY79	pSK281	*
SKB250	*amyE::PmifM gfp-apdP(34-140)-A132E-flag-lacZΩ cat*	PY79	pSK283	*
SKB251	*amyE::PmifM gfp-apdP(34-140)-A132F-flag-lacZΩ cat*	PY79	pSK282	*
SKB252	*amyE::PmifM gfp-apdP(34-140)-A132L-flag-lacZΩ cat*	PY79	pSK292	*
SKB253	*amyE::PmifM gfp-apdP(34-140)-A132Q-flag-lacZΩ cat*	PY79	pSK285	*
SKB254	*amyE::PmifM gfp-apdP(34-140)-A132T-flag-lacZΩ cat*	PY79	pSK284	*
SKB255	*amyE::PmifM gfp-apdP(34-140)-P133F-flag-lacZΩ cat*	PY79	pSK278	*
SKB256	*amyE::PmifM gfp-apdP(34-140)-P133Q-flag-lacZΩ cat*	PY79	pSK276	*
SKB257	*amyE::PmifM gfp-apdP(34-140)-P133W-flag-lacZΩ cat*	PY79	pSK277	*
SKB268	*amyE::PmifM gfp-apdP(34-140)-R131F-flag-lacZΩ cat*	PY79	pSK300	*
SKB270	*amyE::PmifM gfp-apdP(34-140)-R131T-flag-lacZΩ cat*	PY79	pSK301	*
SKB272	*amyE::PmifM gfp-apdP(34-140)-A132C-flag-lacZΩ cat*	PY79	pSK298	*
SKB274	*amyE::PmifM gfp-apdP(34-140)-A132M-flag-lacZΩ cat*	PY79	pSK297	*
SKB276	*amyE::PmifM gfp-apdP(34-140)-A132N-flag-lacZΩ cat*	PY79	pSK299	*
SKB278	*amyE::PmifM gfp-apdP(34-140)-P133D-flag-lacZΩ cat*	PY79	pSK305	*
SKB280	*amyE::PmifM gfp-apdP(34-140)-P133E-flag-lacZΩ cat*	PY79	pSK304	*
SKB282	*amyE::PmifM gfp-apdP(34-140)-P133N-flag-lacZΩ cat*	PY79	pSK306	*
SKB284	*amyE::PmifM gfp-apdP(34-140)-P133R-flag-lacZΩ cat*	PY79	pSK303	*
SKB286	*amyE::PmifM gfp-apdP(34-140)-P133T-flag-lacZΩ cat*	PY79	pSK302	*
SKB288	*amyE::PmifM gfp-apdP(34-140)-A132H-flag-lacZΩ cat*	PY79	pSK311	*
SKB289	*amyE::PmifM gfp-apdP(34-140)-A132I-flag-lacZΩ cat*	PY79	pSK307	*
SKB290	*amyE::PmifM gfp-apdP(34-140)-A132P-flag-lacZΩ cat*	PY79	pSK308	*
SKB291	*amyE::PmifM gfp-apdP(34-140)-A132R-flag-lacZΩ cat*	PY79	pSK309	*
SKB292	*amyE::PmifM gfp-apdP(34-140)-A132Y-flag-lacZΩ cat*	PY79	pSK312	*
SKB293	*amyE::PmifM gfp-apdP(34-140)-P133C-flag-lacZΩ cat*	PY79	pSK275	*
SKB294	*amyE::PmifM gfp-apdP(34-140)-P133K-flag-lacZΩ cat*	PY79	pSK310	*
TJB60	*amyE::PmifM gfp-SLIV_07330(28-129)-myc-lacZΩ cat*	PY79	pTJ83	*
TJB61	*amyE::PmifM gfp-SLIV_07330(28-129)R121A-myc-lacZΩ cat*	PY79	pTJ86	*
TJB62	*amyE::PmifM gfp-SLIV_16130(29-118)-myc-lacZΩ cat*	PY79	pTJ101	*
TJB63	*amyE::PmifM gfp-SLIV_16130(29-118)R105A-myc-lacZΩ cat*	PY79	pTJ105	*
TJB68	*amyE::PmifM gfp-SLIV_18480(213-284)-myc-lacZΩ cat*	PY79	pTJ109	*
TJB69	*amyE::PmifM gfp-SLIV_18480(213-284)R226A-myc-lacZΩ cat*	PY79	pTJ269	*
TJB70	*amyE::PmifM gfp-SLIV_33595(169-234)-myc-lacZΩ cat*	PY79	pTJ108	*
TJB71	*amyE::PmifM gfp-SLIV_33595(169-234)R225A-myc-lacZΩ cat*	PY79	pTJ270	*
TJB72	*amyE::PmifM gfp-SLIV_33605(191-256)-myc-lacZΩ cat*	PY79	pTJ110	*
TJB73	*amyE::PmifM gfp-SLIV_33605(191-256)R247A-myc-lacZΩ cat*	PY79	pTJ275	*
TJB74	*amyE::PmifM gfp-SLIV_27375(196-236)-myc-lacZΩ cat*	PY79	pTJ321	*
TJB75	*amyE::PmifM gfp-SLIV_27375(196-236)R226A-myc-lacZΩ cat*	PY79	pTJ345	*
TJB76	*amyE::PmifM gfp-SLIV_32905(42-81)-myc-lacZΩ cat*	PY79	pTJ322	*
TJB77	*amyE::PmifM gfp-SLIV_32905(42-81)R72A-myc-lacZΩ cat*	PY79	pTJ344	*

(1) Youngman et al. (1984) *Mol. Gen. Genet.*, **195**, 424–33.

(2) Sohmen et al. (2015) *Nat. Commun.*, **6**, 6941.

(3) Sakiyama at al. (2021) *Nucleic Acids Res.*, **49**, 1550–1566.

(4) Morici et al. (2024) *Nat. Commun.,* **15**, 2432.

*This study

Appendix Table S2: *E. coli* strains

strain	genotype	parent	plasmid 1	plasmid 2
SKE39	*gfp-apdP(34-140)-flag-lacZ*	JM109	pSK92	
SKE59	*gfp-apdP(34-140)-R131A-flag-lacZ*	JM109	pSK107	
SKE60	*gfp-apdP(34-140)-A132S-flag-lacZ*	JM109	pSK106	
SKE61	*gfp-apdP(34-140)-P133A-flag-lacZ*	JM109	pSK101	
SKE68	*gfp-apdP(34-140)-P133G-flag-lacZ*	JM109	pSK113	
SKE71	*gfp-apdP(34-140)-R131D-flag-lacZ*	JM109	pSK254	
SKE72	*gfp-apdP(34-140)-R131E-flag-lacZ*	JM109	pSK251	
SKE73	*gfp-apdP(34-140)-R131G-flag-lacZ*	JM109	pSK249	
SKE74	*gfp-apdP(34-140)-R131I-flag-lacZ*	JM109	pSK250	
SKE75	*gfp-apdP(34-140)-R131K-flag-lacZ*	JM109	pSK252	
SKE76	*gfp-apdP(34-140)-R131L-flag-lacZ*	JM109	pSK256	
SKE77	*gfp-apdP(34-140)-R131M-flag-lacZ*	JM109	pSK247	
SKE78	*gfp-apdP(34-140)-R131S-flag-lacZ*	JM109	pSK255	
SKE79	*gfp-apdP(34-140)-R131V-flag-lacZ*	JM109	pSK248	
SKE80	*gfp-apdP(34-140)-R131W-flag-lacZ*	JM109	pSK253	
SKE81	*gfp-apdP(34-140)-A132G-flag-lacZ*	JM109	pSK258	
SKE82	*gfp-apdP(34-140)-A132V-flag-lacZ*	JM109	pSK257	
SKE83	*gfp-apdP(34-140)-A132W-flag-lacZ*	JM109	pSK259	
SKE84	*gfp-apdP(34-140)-R131C-flag-lacZ*	JM109	pSK265	
SKE85	*gfp-apdP(34-140)-R131H-flag-lacZ*	JM109	pSK268	
SKE86	*gfp-apdP(34-140)-R131N-flag-lacZ*	JM109	pSK266	
SKE87	*gfp-apdP(34-140)-R131P-flag-lacZ*	JM109	pSK269	
SKE88	*gfp-apdP(34-140)-R131Q-flag-lacZ*	JM109	pSK274	
SKE89	*gfp-apdP(34-140)-R131Y-flag-lacZ*	JM109	pSK267	
SKE90	*gfp-apdP(34-140)-A132D-flag-lacZ*	JM109	pSK281	
SKE91	*gfp-apdP(34-140)-A132E-flag-lacZ*	JM109	pSK283	
SKE92	*gfp-apdP(34-140)-A132F-flag-lacZ*	JM109	pSK282	
SKE93	*gfp-apdP(34-140)-A132K-flag-lacZ*	JM109	pSK270	
SKE94	*gfp-apdP(34-140)-A132L-flag-lacZ*	JM109	pSK292	
SKE95	*gfp-apdP(34-140)-A132Q-flag-lacZ*	JM109	pSK285	
SKE96	*gfp-apdP(34-140)-A132T-flag-lacZ*	JM109	pSK284	
SKE97	*gfp-apdP(34-140)-P133H-flag-lacZ*	JM109	pSK272	
SKE98	*gfp-apdP(34-140)-P133I-flag-lacZ*	JM109	pSK261	
SKE99	*gfp-apdP(34-140)-P133L-flag-lacZ*	JM109	pSK273	
SKE100	*gfp-apdP(34-140)-P133M-flag-lacZ*	JM109	pSK260	
SKE101	*gfp-apdP(34-140)-P133S-flag-lacZ*	JM109	pSK264	
SKE102	*gfp-apdP(34-140)-P133V-flag-lacZ*	JM109	pSK271	
SKE103	*gfp-apdP(34-140)-P133Y-flag-lacZ*	JM109	pSK262	
SKE105	*gfp-apdP(34-140)-P133F-flag-lacZ*	JM109	pSK278	
SKE106	*gfp-apdP(34-140)-P133Q-flag-lacZ*	JM109	pSK276	
SKE107	*gfp-apdP(34-140)-P133W-flag-lacZ*	JM109	pSK277	

SKE108	*gfp-apdP(34-140)-P133C-flag-lacZ*	JM109	pSK275	
SKE109	*gfp-apdP(34-140)-R131F-flag-lacZ*	JM109	pSK300	
SKE110	*gfp-apdP(34-140)-R131T-flag-lacZ*	JM109	pSK301	
SKE111	*gfp-apdP(34-140)-A132C-flag-lacZ*	JM109	pSK298	
SKE112	*gfp-apdP(34-140)-A132M-flag-lacZ*	JM109	pSK297	
SKE113	*gfp-apdP(34-140)-A132N-flag-lacZ*	JM109	pSK299	
SKE114	*gfp-apdP(34-140)-P133D-flag-lacZ*	JM109	pSK305	
SKE115	*gfp-apdP(34-140)-P133E-flag-lacZ*	JM109	pSK304	
SKE116	*gfp-apdP(34-140)-P133N-flag-lacZ*	JM109	pSK306	
SKE117	*gfp-apdP(34-140)-P133R-flag-lacZ*	JM109	pSK303	
SKE118	*gfp-apdP(34-140)-P133T-flag-lacZ*	JM109	pSK302	
SKE119	*gfp-apdP(34-140)-A132H-flag-lacZ*	JM109	pSK311	
SKE120	*gfp-apdP(34-140)-A132I-flag-lacZ*	JM109	pSK307	
SKE121	*gfp-apdP(34-140)-A132P-flag-lacZ*	JM109	pSK308	
SKE122	*gfp-apdP(34-140)-A132R-flag-lacZ*	JM109	pSK309	
SKE123	*gfp-apdP(34-140)-A132Y-flag-lacZ*	JM109	pSK312	
SKE124	*gfp-apdP(34-140)-P133K-flag-lacZ*	JM109	pSK310	
KFE684	*gfp-ApdP(34-140)-myc-lacZ*	JM109	pCH2429	
KFE685	*gfp-ApdP(34-140)-R131A-myc-lacZ*	JM109	pCH2430	
KFE679	*gfp-ApdP(34-140)-A132G-myc-lacZWcat*	JM109	pKIG1441	
KFE678	*gfp-ApdP(34-140)-P134G-myc-lacZWcat*	JM109	pKIG1439	
KFE676	*gfp-ApdP(34-140)-P133G-myc-lacZWcat*	JM109	pKIG1435	
KFE680	*gfp-ApdP(34-140)-R131A/P133G-myc-lacZWcat*	JM109	pKIG1443	
KFE681	*gfp-secM(38-170)-myc-lacZ*	JM109	pCH2421	
KFE683	*gfp-secM(38-170)-R163A-myc-lacZ*	JM109	pCH2435	
KFE682	*gfp-secM(38-170)-P166A-myc-lacZ*	JM109	pCH2422	
KFE672	*gfp-secM(38-170)-G165P-myc-lacZ*	JM109	pKIG1427	
KFE675	*gfp-secM(38-170)-R163A/G165P-myc-lacZ*	JM109	pKIG1433	
KFE674	*gfp-secM(38-170)-A164P/G165P-myc-lacZ*	JM109	pKIG1431	
KFE673	*gfp-secM(38-170)-G165P/P166G-myc-lacZ*	JM109	pKIG1429	
SCE664	*gfp-secM(38-170)-myc-lacZ / Plac rplV+*	JM109	pCH2421	pCH2616
SCE666	*gfp-secM(38-170)-P166A-myc-lacZ / Plac rplV+*	JM109	pCH2422	pCH2616
SCE668	*gfp-secM(38-170)-G165P-myc-lacZ / Plac rplV+*	JM109	pKIG1427	pCH2616
SCE670	*gfp-secM(38-170)-A164P/G165P-myc-lacZ / Plac rplV+*	JM109	pKIG1431	pCH2616
SCE672	*gfp-secM(38-170)-myc-lacZ / Plac rplV(dM96K97R98)*	JM109	pCH2421	pCH2617
SCE674	*gfp-secM(38-170)-P166A-myc-lacZ / Plac rplV(dM96K97R98)*	JM109	pCH2422	pCH2617
SCE676	*gfp-secM(38-170)-G165P-myc-lacZ / Plac rplV(dM96K97R98)*	JM109	pKIG1427	pCH2617
SCE678	*gfp-secM(38-170)-A164P/G165P-myc-lacZ / Plac rplV(dM96K97R98)*	JM109	pKIG1431	pCH2617

Appendix Table S3: Plasmids

plasmid	gene	ref	PCR 1			PCR 2		
			fw primer 1	rv primer 1	template 1	fw primer 2	rv primer 2	template 2
pCH2124	*gfp-apcA(62-108)-flag-lacZ*	(1)						
pCH2125	*gfp-apdA(39-128)-flag-lacZ*	(1)						
pCH2126	*gfp-apdP(34-140)-flag-lacZ*	(1)						
pCH2128	*gfp-apdP(34-140)-R131A-flag-lacZ*	(1)						
pCH2134	*gfp-apdA(39-128)-R120A-flag-lacZ*	(1)						
pCH2139	*gfp-secM38-flag-yidC2(6)-lacZ*	(2)						
pCH2311	*gfp-myc-lacZ*	(3)						
pCH2312	*gfp-secM(38-170)-myc-lacZ*	(4)						
pCH2316	*gfp-apcA(62-108)-myc-lacZ*	*	gfp238-fw	Re_Ca-myc27-rv	pCH2124	myc-lacZ7-fw	GFP238-rv	pSK69
pCH2318	*gfp-apdA(39-128)-myc-lacZ*	*	gfp238-fw	Aj_Da-myc27-rv	pCH2125	myc-lacZ7-fw	GFP238-rv	pSK69
pCH2319	*gfp-apdA(39-128)-R120A-myc-lacZ*	*	gfp238-fw	Aj_Da-myc27-rv	pCH2134	myc-lacZ7-fw	GFP238-rv	pSK69
pCH2320	*gfp-apdP(34-140)-myc-lacZ*	*	gfp238-fw	Sm_Db-myc27-rv	pCH2126	myc-lacZ7-fw	GFP238-rv	pSK69
pCH2321	*gfp-apdP(34-140)-R131A-myc-lacZ*	*	gfp238-fw	Sm_Db-myc27-rv	pCH2128	myc-lacZ7-fw	GFP238-rv	pSK69
pCH2376	*gfp-apcA(62-108)-G105P-myc-lacZ*	*	Re_apcA-G105P-myc-fw	ampR 121-128(Tm62)-antisense		ampR 121-128(Tm62)	Re_apcA-P104-rv	pCH2316
pCH2421	*gfp-secM(38-170)-myc-lacZ*	(4)						
pCH2422	*gfp-secM(38-170)-P166A-myc-lacZ*	(4)						
pCH2425	*gfp-apcA(62-108)-myc-lacZWcat*	*	myc-lacZ7-CTC-fw	myc27-rv	pCH2316			
pCH2426	*gfp-apcA(62-108)-R102A-myc-lacZWcat*	*	myc-lacZ7-CTC-fw	myc27-rv	pCH2317			
pCH2427	*gfp-apdA(39-128)-myc-lacZ*	*	myc-lacZ7-CTC-fw	myc27-rv	pCH2318			
pCH2428	*gfp-apdA(39-128)-R120A-myc-lacZ*	*	myc-lacZ7-CTC-fw	myc27-rv	pCH2319			
pCH2429	*gfp-apdP(34-140)-myc-lacZ*	*	myc-lacZ7-CTC-fw	myc27-rv	pCH2320			
pCH2430	*gfp-apdP(34-140)-R131A-myc-lacZ*	*	myc-lacZ7-CTC-fw	myc27-rv	pCH2321			
pCH2435	*gfp-secM(38-170)-R163A-myc-lacZ*	(4)						
pCH2437	*gfp-apcA(62-108)-G105P-myc-lacZWcat*	*	myc-lacZ7-CTC-fw	myc27-rv	pCH2376			
pCH2503	*gfp-apdP(34-130)-apdA(120-128)-myc-lacZ*	*	Dp130-Da120-fw	ampR 121-128(Tm62)-antisense	pCH2318	ampR 121-128(Tm62)	Dp130-rv	pCH2320
pCH2504	*gfp-apdP(34-125)-apdA(115-128)-myc-lacZ*	*	Dp125-Da115-fw	ampR 121-128(Tm62)-antisense	pCH2318	ampR 121-128(Tm62)	Dp125-rv	pCH2320
pCH2505	*gfp-apdP(34-120)-apdA(110-128)-myc-lacZ*	*	Dp120-Da110-fw	ampR 121-128(Tm62)-antisense	pCH2318	ampR 121-128(Tm62)	Dp120-rv	pCH2320
pCH2506	*gfp-apdP(34-115)-apdA(105-128)-myc-lacZ*	*	Dp115-Da105-fw	ampR 121-128(Tm62)-antisense	pCH2318	ampR 121-128(Tm62)	Dp115-rv	pCH2320
pCH2507	*gfp-apdA(39-119)-apdP(131-140)-myc-lacZ*	*	Da119-Dp131-fw	ampR 121-128(Tm62)-antisense	pCH2320	ampR 121-128(Tm62)	Da119-rv	pCH2318
pCH2508	*gfp-apdA(39-114)-apdP(126-140)-myc-lacZ*	*	Da114-Dp126-fw	ampR 121-128(Tm62)-antisense	pCH2320	ampR 121-128(Tm62)	Da114-rv	pCH2318
pCH2509	*gfp-apdA(39-109)-apdP(121-140)-myc-lacZ*	*	Da109-Dp121-fw	ampR 121-128(Tm62)-antisense	pCH2320	ampR 121-128(Tm62)	Da109-rv	pCH2318
pCH2510	*gfp-apdA(39-104)-apdP(116-140)-myc-lacZ*	*	Da104-Dp116-fw	ampR 121-128(Tm62)-antisense	pCH2320	ampR 121-128(Tm62)	Da104-rv	pCH2318
pCH2599	*gfp-apcA(62-108)-A103G/G105P(RGPP)-myc-lacZWcat*		ApcA_RGPP-myc-fw	ampR 121-128(Tm62)-antisense	pCH2437	ampR 121-128(Tm62)	Re_ApdA_R102-rv	pCH2437
pCH2600	*gfp-apcA(62-108)-R102A/G105P(AAPP)-myc-lacZWcat*		ApcA_AAPP-myc-fw	ampR 121-128(Tm62)-antisense	pCH2437	ampR 121-128(Tm62)	Re_ApdA_P101-rv	pCH2437
pCH2601	*gfp-apcA(62-108)-P104G/G105P(RAGP)-myc-lacZWcat*		ApcA_RAGP-myc-fw	ampR 121-128(Tm62)-antisense	pCH2437	ampR 121-128(Tm62)	Re_ApdA_A103-rv	pCH2437
pCH2616	*Plac rplV+*		pTWV229-AmpR-TAA-fw	pTWV229-AmpR-SD-rv	pNH106	pTWV228-cat-SD-fw	pTWV228-cat-rv	pSTV28
pCH2617	*Plac rplV(dM96K97R98)*		pTWV229-AmpR-TAA-fw	pTWV229-AmpR-SD-rv	pNH112	pTWV228-cat-SD-fw	pTWV228-cat-rv	pSTV28
pSK63	*gfp-apcA(62-108)-R102A-flag-lacZ*	*	Ca-R102A-fw	amyE-Fw2	pCH2124	Ca-R102A-rv	amyE-Fw2-rv	pCH2124
pSK64	*gfp-apcA(62-108)-A103S-flag-lacZ*	*	Ca-A103S-fw	amyE-Fw2	pCH2124	Ca-A103S-rv	amyE-Fw2-rv	pCH2124
pSK66	*gfp-apcA(62-108)-P104A-flag-lacZ*	*	Ca-P104A-fw	amyE-Fw2	pCH2124	Ca-P104A-rv	amyE-Fw2-rv	pCH2124
pSK69	*gfp-apdP(34-140)-flag-lacZ*	*	pMW118-ori-fw	pMW118-ori-rv	pMW118	pCH2126-del-ori-fw	pMW118-ori-rv	pCH2126
pSK87	*gfp-apdA(39-128)-A121S-flag-lacZ*	*	Da-A121S-fw	amyE-Fw2	pCH2125	Da-A121S-rv	amyE-Fw2-rv	pCH2125
pSK88	*gfp-apdA(39-128)-P122A-flag-lacZ*	*	Da-P122A-fw	amyE-Fw2	pCH2125	Da-P122A-rv	amyE-Fw2-rv	pCH2125
pSK92	*gfp-apdP(34-140)-flag-lacZ*	*	Db-lacZ1-9del-fw	amyE-Fw2	pSK69	Db-lacZ1-9del-rv	amyE-Fw2-rv	pSK69
pSK101	*gfp-apdP(34-140)-P133A-flag-lacZ*	*	Db-P133A-fw	amyE-Fw2	pSK92	Db-P133A-rv	amyE-Fw2-rv	pSK92
pSK106	*gfp-apdP(34-140)-A132S-flag-lacZ*	*	Db-A132S-fw	amyE-Fw2	pSK92	Db-A132S-rv	amyE-Fw2-rv	pSK92
pSK107	*gfp-apdP(34-140)-R131A-flag-lacZ*	*	Sinor medic-Db-R131A-fw	amyE-Fw2	pSK92	Sinor medic-Db-R131A-rv	amyE-Fw2-rv	pSK92
pSK111	*gfp-apcA(62-108)-P104G-flag-lacZ*	*	Ca-P104G-fw	amyE-Fw2	pCH2124	Ca-P104A-rv	amyE-Fw2-rv	pCH2124
pSK112	*gfp-apdA(39-128)-P122G-flag-lacZ*	*	Da-P122G-fw	amyE-Fw2	pCH2125	Da-P122A-rv	amyE-Fw2-rv	pCH2125
pSK113	*gfp-apdP(34-140)-P133G-flag-lacZ*	*	Db-P133G-fw	amyE-Fw2	pSK92	Db-P133A-rv	amyE-Fw2-rv	pSK92
pSK122	*gfp-apcA(62-108)-P104R-flag-lacZ*	*	Ca-P104X-fw	amyE-Fw2	pCH2124	Ca-R102A-rv	amyE-Fw2-rv	pCH2124
pSK123	*gfp-apcA(62-108)-P104Y-flag-lacZ*	*	Ca-P104X-fw	amyE-Fw2	pCH2124	Ca-R102A-rv	amyE-Fw2-rv	pCH2124
pSK126	*gfp-apcA(62-108)-R102S-flag-lacZ*	*	Ca-R102X-fw	amyE-Fw2	pCH2124	Ca-R102A-rv	amyE-Fw2-rv	pCH2124
pSK127	*gfp-apcA(62-108)-R102D-flag-lacZ*	*	Ca-R102X-fw	amyE-Fw2	pCH2124	Ca-R102A-rv	amyE-Fw2-rv	pCH2124
pSK128	*gfp-apcA(62-108)-R102I-flag-lacZ*	*	Ca-R102X-fw	amyE-Fw2	pCH2124	Ca-R102A-rv	amyE-Fw2-rv	pCH2124
pSK136	*gfp-apcA(62-108)-R102W-flag-lacZ*	*	Ca-R102X-fw	amyE-Fw2	pCH2124	Ca-R102A-rv	amyE-Fw2-rv	pCH2124
pSK137	*gfp-apcA(62-108)-R102P-flag-lacZ*	*	Ca-R102X-fw	amyE-Fw2	pCH2124	Ca-R102A-rv	amyE-Fw2-rv	pCH2124
pSK138	*gfp-apcA(62-108)-P104S-flag-lacZ*	*	Ca-P104X-fw	amyE-Fw2	pCH2124	Ca-P104A-rv	amyE-Fw2-rv	pCH2124
pSK139	*gfp-apcA(62-108)-P104K-flag-lacZ*	*	Ca-P104X-fw	amyE-Fw2	pCH2124	Ca-P104A-rv	amyE-Fw2-rv	pCH2124
pSK140	*gfp-apcA(62-108)-A103E-flag-lacZ*	*	Ca-A103X-fw	amyE-Fw2	pCH2124	Ca-A103S-rv	amyE-Fw2-rv	pCH2124
pSK142	*gfp-apcA(62-108)-A103G-flag-lacZ*	*	Ca-A103X-fw	amyE-Fw2	pCH2124	Ca-A103S-rv	amyE-Fw2-rv	pCH2124
pSK143	*gfp-apcA(62-108)-A103V-flag-lacZ*	*	Ca-A103X-fw	amyE-Fw2	pCH2124	Ca-A103S-rv	amyE-Fw2-rv	pCH2124
pSK148	*gfp-apcA(62-108)-A103H-flag-lacZ*	*	Ca-A103X-fw	amyE-Fw2	pCH2124	Ca-A103S-rv	amyE-Fw2-rv	pCH2124
pSK149	*gfp-apcA(62-108)-A103L-flag-lacZ*	*	Ca-A103X-fw	amyE-Fw2	pCH2124	Ca-A103S-rv	amyE-Fw2-rv	pCH2124
pSK150	*gfp-apcA(62-108)-A103C-flag-lacZ*	*	Ca-A103X-fw	amyE-Fw2	pCH2124	Ca-A103S-rv	amyE-Fw2-rv	pCH2124
pSK151	*gfp-apcA(62-108)-A103I-flag-lacZ*	*	Ca-A103X-fw	amyE-Fw2	pCH2124	Ca-A103S-rv	amyE-Fw2-rv	pCH2124
pSK152	*gfp-apcA(62-108)-A103W-flag-lacZ*	*	Ca-A103X-fw	amyE-Fw2	pCH2124	Ca-A103S-rv	amyE-Fw2-rv	pCH2124
pSK153	*gfp-apcA(62-108)-A103R-flag-lacZ*	*	Ca-A103X-fw	amyE-Fw2	pCH2124	Ca-A103S-rv	amyE-Fw2-rv	pCH2124
pSK154	*gfp-apcA(62-108)-A103P-flag-lacZ*	*	Ca-A103X-fw	amyE-Fw2	pCH2124	Ca-A103S-rv	amyE-Fw2-rv	pCH2124
pSK155	*gfp-apcA(62-108)-P104T-flag-lacZ*	*	Ca-P104T-fw	amyE-Fw2	pCH2124	Ca-P104A-rv	amyE-Fw2-rv	pCH2124
pSK156	*gfp-apcA(62-108)-P104Q-flag-lacZ*	*	Ca-P104Q-fw	amyE-Fw2	pCH2124	Ca-P104A-rv	amyE-Fw2-rv	pCH2124
pSK157	*gfp-apcA(62-108)-P104F-flag-lacZ*	*	Ca-P104F-fw	amyE-Fw2	pCH2124	Ca-P104A-rv	amyE-Fw2-rv	pCH2124
pSK158	*gfp-apcA(62-108)-P104C-flag-lacZ*	*	Ca-P104C-fw	amyE-Fw2	pCH2124	Ca-P104A-rv	amyE-Fw2-rv	pCH2124
pSK159	*gfp-apcA(62-108)-A103M-flag-lacZ*	*	Ca-A103M-Fw	amyE-Fw2	pCH2124	Ca-R102A-rv	amyE-Fw2-rv	pCH2124
pSK160	*gfp-apcA(62-108)-A103K-flag-lacZ*	*	Ca-A103K-Fw	amyE-Fw2	pCH2124	Ca-R102A-rv	amyE-Fw2-rv	pCH2124
pSK161	*gfp-apcA(62-108)-A103N-flag-lacZ*	*	Ca-A103N-Fw	amyE-Fw2	pCH2124	Ca-R102A-rv	amyE-Fw2-rv	pCH2124
pSK162	*gfp-apcA(62-108)-R102F-flag-lacZ*	*	Ca-R102X-Fw2	amyE-Fw2	pCH2124	Ca-R102A-rv	amyE-Fw2-rv	pCH2124
pSK163	*gfp-apcA(62-108)-R102K-flag-lacZ*	*	Ca-R102X-Fw2	amyE-Fw2	pCH2124	Ca-R102A-rv	amyE-Fw2-rv	pCH2124
pSK164	*gfp-apcA(62-108)-R102C-flag-lacZ*	*	Ca-R102X-Fw2	amyE-Fw2	pCH2124	Ca-R102A-rv	amyE-Fw2-rv	pCH2124
pSK165	*gfp-apcA(62-108)-R102M-flag-lacZ*	*	Ca-R102X-Fw2	amyE-Fw2	pCH2124	Ca-R102A-rv	amyE-Fw2-rv	pCH2124
pSK166	*gfp-apcA(62-108)-A103Q-flag-lacZ*	*	Ca-A103Q-Fw	amyE-Fw2	pCH2124	Ca-R102A-rv	amyE-Fw2-rv	pCH2124
pSK167	*gfp-apcA(62-108)-P104N-flag-lacZ*	*	Ca-P104X-fw	amyE-Fw2	pCH2124	Ca-P104A-rv	amyE-Fw2-rv	pCH2124
pSK168	*gfp-apcA(62-108)-P104L-flag-lacZ*	*	Ca-P104X-fw	amyE-Fw2	pCH2124	Ca-P104A-rv	amyE-Fw2-rv	pCH2124
pSK169	*gfp-apcA(62-108)-A103D-flag-lacZ*	*	Ca-A103X-fw	amyE-Fw2	pCH2124	Ca-A103S-rv	amyE-Fw2-rv	pCH2124
pSK170	*gfp-apcA(62-108)-A103Y-flag-lacZ*	*	Ca-A103X-fw	amyE-Fw2	pCH2124	Ca-A103S-rv	amyE-Fw2-rv	pCH2124
pSK171	*gfp-apcA(62-108)-P104I-flag-lacZ*	*	Ca-P104X-fw	amyE-Fw2	pCH2124	Ca-R102A-rv	amyE-Fw2-rv	pCH2124
pSK172	*gfp-apcA(62-108)-P104M-flag-lacZ*	*	Ca-P104X-fw	amyE-Fw2	pCH2124	Ca-R102A-rv	amyE-Fw2-rv	pCH2124
pSK173	*gfp-apcA(62-108)-P104D-flag-lacZ*	*	Ca-P104X-fw	amyE-Fw2	pCH2124	Ca-R102A-rv	amyE-Fw2-rv	pCH2124
pSK174	*gfp-apcA(62-108)-P104V-flag-lacZ*	*	Ca-P104X-fw	amyE-Fw2	pCH2124	Ca-R102A-rv	amyE-Fw2-rv	pCH2124
pSK175	*gfp-apcA(62-108)-R102V-flag-lacZ*	*	Ca-R102V-Fw	amyE-Fw2	pCH2124	Ca-R102A-rv	amyE-Fw2-rv	pCH2124
pSK176	*gfp-apcA(62-108)-A103T-flag-lacZ*	*	Ca-A103X-fw	amyE-Fw2	pCH2124	Ca-A103S-rv	amyE-Fw2-rv	pCH2124
pSK177	*gfp-apcA(62-108)-A103F-flag-lacZ*	*	Ca-A103X-fw	amyE-Fw2	pCH2124	Ca-A103S-rv	amyE-Fw2-rv	pCH2124
pSK178	*gfp-apcA(62-108)-P104E-flag-lacZ*	*	Ca-P104X-fw	amyE-Fw2	pCH2124	Ca-R102A-rv	amyE-Fw2-rv	pCH2124
pSK179	*gfp-apcA(62-108)-P104W-flag-lacZ*	*	Ca-P104X-fw	amyE-Fw2	pCH2124	Ca-R102A-rv	amyE-Fw2-rv	pCH2124
pSK181	*gfp-apcA(62-108)-R102Y-flag-lacZ*	*	Ca-R102Y-Fw	amyE-Fw2	pCH2124	Ca-R102A-rv	amyE-Fw2-rv	pCH2124
pSK182	*gfp-apcA(62-108)-R102E-flag-lacZ*	*	Ca-R102E-Fw	amyE-Fw2	pCH2124	Ca-R102A-rv	amyE-Fw2-rv	pCH2124
pSK183	*gfp-apcA(62-108)-P104H-flag-lacZ*	*	Ca-P104H-fw	amyE-Fw2	pCH2124	Ca-P104A-rv	amyE-Fw2-rv	pCH2124
pSK184	*gfp-apcA(62-108)-R102T-flag-lacZ*	*	Ca-R102X-Fw	amyE-Fw2	pCH2124	Ca-R102A-rv	amyE-Fw2-rv	pCH2124
pSK186	*gfp-apcA(62-108)-R102G-flag-lacZ*	*	Ca-R102X-Fw	amyE-Fw2	pCH2124	Ca-R102A-rv	amyE-Fw2-rv	pCH2124
pSK187	*gfp-apcA(62-108)-R102H-flag-lacZ*	*	Ca-R102X-Fw	amyE-Fw2	pCH2124	Ca-R102A-rv	amyE-Fw2-rv	pCH2124
pSK188	*gfp-apcA(62-108)-R102L-flag-lacZ*	*	Ca-R102X-Fw	amyE-Fw2	pCH2124	Ca-R102A-rv	amyE-Fw2-rv	pCH2124
pSK189	*gfp-apcA(62-108)-R102Q-flag-lacZ*	*	Ca-R102X-Fw	amyE-Fw2	pCH2124	Ca-R102A-rv	amyE-Fw2-rv	pCH2124
pSK190	*gfp-apcA(62-108)-R102N-flag-lacZ*	*	Ca-R102X-Fw	amyE-Fw2	pCH2124	Ca-R102A-rv	amyE-Fw2-rv	pCH2124
pSK192	*gfp-apdA(39-128)-R120M-flag-lacZ*	*	Da-R120X-Fw	amyE-Fw2	pCH2125	Da-R120X-Rv	amyE-Fw2-rv	pCH2125
pSK193	*gfp-apdA(39-128)-R120K-flag-lacZ*	*	Da-R120X-Fw	amyE-Fw2	pCH2125	Da-R120X-Rv	amyE-Fw2-rv	pCH2125
pSK194	*gfp-apdA(39-128)-R120L-flag-lacZ*	*	Da-R120X-Fw	amyE-Fw2	pCH2125	Da-R120X-Rv	amyE-Fw2-rv	pCH2125
pSK195	*gfp-apdA(39-128)-R120E-flag-lacZ*	*	Da-R120X-Fw	amyE-Fw2	pCH2125	Da-R120X-Rv	amyE-Fw2-rv	pCH2125
pSK196	*gfp-apdA(39-128)-R120G-flag-lacZ*	*	Da-R120X-Fw	amyE-Fw2	pCH2125	Da-R120X-Rv	amyE-Fw2-rv	pCH2125
pSK197	*gfp-apdA(39-128)-R120Y-flag-lacZ*	*	Da-R120X-Fw	amyE-Fw2	pCH2125	Da-R120X-Rv	amyE-Fw2-rv	pCH2125
pSK198	*gfp-apdA(39-128)-R120P-flag-lacZ*	*	Da-R120X-Fw	amyE-Fw2	pCH2125	Da-R120X-Rv	amyE-Fw2-rv	pCH2125
pSK199	*gfp-apdA(39-128)-R120V-flag-lacZ*	*	Da-R120X-Fw	amyE-Fw2	pCH2125	Da-R120X-Rv	amyE-Fw2-rv	pCH2125
pSK200	*gfp-apdA(39-128)-R120W-flag-lacZ*	*	Da-R120X-Fw	amyE-Fw2	pCH2125	Da-R120X-Rv	amyE-Fw2-rv	pCH2125
pSK201	*gfp-apdA(39-128)-R120C-flag-lacZ*	*	Da-R120X-Fw	amyE-Fw2	pCH2125	Da-R120X-Rv	amyE-Fw2-rv	pCH2125
pSK202	*gfp-apdA(39-128)-R120Q-flag-lacZ*	*	Da-R120X-Fw	amyE-Fw2	pCH2125	Da-R120X-Rv	amyE-Fw2-rv	pCH2125
pSK203	*gfp-apdA(39-128)-R120T-flag-lacZ*	*	Da-R120X-Fw	amyE-Fw2	pCH2125	Da-R120X-Rv	amyE-Fw2-rv	pCH2125
pSK204	*gfp-apdA(39-128)-A121K-flag-lacZ*	*	Da-A121X-Fw	amyE-Fw2	pCH2125	Da-A121X-Rv	amyE-Fw2-rv	pCH2125
pSK205	*gfp-apdA(39-128)-A121R-flag-lacZ*	*	Da-A121X-Fw	amyE-Fw2	pCH2125	Da-A121X-Rv	amyE-Fw2-rv	pCH2125
pSK206	*gfp-apdA(39-128)-A121L-flag-lacZ*	*	Da-A121X-Fw	amyE-Fw2	pCH2125	Da-A121X-Rv	amyE-Fw2-rv	pCH2125
pSK207	*gfp-apdA(39-128)-A121D-flag-lacZ*	*	Da-A121X-Fw	amyE-Fw2	pCH2125	Da-A121X-Rv	amyE-Fw2-rv	pCH2125
pSK208	*gfp-apdA(39-128)-A121V-flag-lacZ*	*	Da-A121X-Fw	amyE-Fw2	pCH2125	Da-A121X-Rv	amyE-Fw2-rv	pCH2125
pSK209	*gfp-apdA(39-128)-A121E-flag-lacZ*	*	Da-A121X-Fw	amyE-Fw2	pCH2125	Da-A121X-Rv	amyE-Fw2-rv	pCH2125
pSK210	*gfp-apdA(39-128)-A121C-flag-lacZ*	*	Da-A121X-Fw	amyE-Fw2	pCH2125	Da-A121X-Rv	amyE-Fw2-rv	pCH2125
pSK211	*gfp-apdA(39-128)-A121T-flag-lacZ*	*	Da-A121X-Fw	amyE-Fw2	pCH2125	Da-A121X-Rv	amyE-Fw2-rv	pCH2125
pSK212	*gfp-apdA(39-128)-R120D-flag-lacZ*	*	Da-R120D-Fw	amyE-Fw2	pCH2125	Da-R120X-Rv	amyE-Fw2-rv	pCH2125
pSK213	*gfp-apdA(39-128)-R120F-flag-lacZ*	*	Da-R120F-Fw	amyE-Fw2	pCH2125	Da-R120X-Rv	amyE-Fw2-rv	pCH2125
pSK214	*gfp-apdA(39-128)-R120H-flag-lacZ*	*	Da-R120H-Fw	amyE-Fw2	pCH2125	Da-R120X-Rv	amyE-Fw2-rv	pCH2125
pSK215	*gfp-apdA(39-128)-R120S-flag-lacZ*	*	Da-R120X-Fw2	amyE-Fw2	pCH2125	Da-R120X-Rv	amyE-Fw2-rv	pCH2125
pSK216	*gfp-apdA(39-128)-R120I-flag-lacZ*	*	Da-R120X-Fw2	amyE-Fw2	pCH2125	Da-R120X-Rv	amyE-Fw2-rv	pCH2125
pSK217	*gfp-apdA(39-128)-P122L-flag-lacZ*	*	Da-P122X-Fw	amyE-Fw2	pCH2125	Da-P122X-Rv	amyE-Fw2-rv	pCH2125
pSK218	*gfp-apdA(39-128)-P122D-flag-lacZ*	*	Da-P122X-Fw	amyE-Fw2	pCH2125	Da-P122X-Fw	amyE-Fw2-rv	pCH2125
pSK219	*gfp-apdA(39-128)-P122E-flag-lacZ*	*	Da-P122X-Fw	amyE-Fw2	pCH2125	Da-P122X-Fw	amyE-Fw2-rv	pCH2125
pSK220	*gfp-apdA(39-128)-P122S-flag-lacZ*	*	Da-P122X-Fw	amyE-Fw2	pCH2125	Da-P122X-Fw	amyE-Fw2-rv	pCH2125
pSK221	*gfp-apdA(39-128)-P122F-flag-lacZ*	*	Da-P122X-Fw	amyE-Fw2	pCH2125	Da-P122X-Fw	amyE-Fw2-rv	pCH2125
pSK222	*gfp-apdA(39-128)-P122V-flag-lacZ*	*	Da-P122X-Fw	amyE-Fw2	pCH2125	Da-P122X-Fw	amyE-Fw2-rv	pCH2125

pSK223	*gfp-apdA(39-128)-P122Q-flag-lacZ*	*	Da-P122X-Fw	amyE-Fw2	pCH2125	Da-P122X-Fw	amyE-Fw2-rv	pCH2125
pSK224	*gfp-apdA(39-128)-P122R-flag-lacZ*	*	Da-P122X-Fw	amyE-Fw2	pCH2125	Da-P122X-Fw	amyE-Fw2-rv	pCH2125
pSK225	*gfp-apdA(39-128)-P122M-flag-lacZ*	*	Da-P122X-Fw	amyE-Fw2	pCH2125	Da-P122X-Fw	amyE-Fw2-rv	pCH2125
pSK226	*gfp-apdA(39-128)-P122W-flag-lacZ*	*	Da-P122X-Fw	amyE-Fw2	pCH2125	Da-P122X-Fw	amyE-Fw2-rv	pCH2125
pSK228	*gfp-apdA(39-128)-A121M-flag-lacZ*	*	Da-A121IM-fw	amyE-Fw2	pCH2125	Da-R120X-Rv	amyE-Fw2-rv	pCH2125
pSK229	*gfp-apdA(39-128)-A121Q-flag-lacZ*	*	Da-A121HQP-fw	amyE-Fw2	pCH2125	Da-R120X-Rv	amyE-Fw2-rv	pCH2125
pSK230	*gfp-apdA(39-128)-A121H-flag-lacZ*	*	Da-A121HQP-fw	amyE-Fw2	pCH2125	Da-R120X-Rv	amyE-Fw2-rv	pCH2125
pSK231	*gfp-apdA(39-128)-A121P-flag-lacZ*	*	Da-A121HQP-fw	amyE-Fw2	pCH2125	Da-R120X-Rv	amyE-Fw2-rv	pCH2125
pSK232	*gfp-apdA(39-128)-A121F-flag-lacZ*	*	Da-A121F-fw	amyE-Fw2	pCH2125	Da-R120X-Rv	amyE-Fw2-rv	pCH2125
pSK233	*gfp-apdA(39-128)-A121W-flag-lacZ*	*	Da-A121W-fw	amyE-Fw2	pCH2125	Da-R120X-Rv	amyE-Fw2-rv	pCH2125
pSK234	*gfp-apdA(39-128)-A121G-flag-lacZ*	*	Da-A121G-fw	amyE-Fw2	pCH2125	Da-R120X-Rv	amyE-Fw2-rv	pCH2125
pSK235	*gfp-apdA(39-128)-A121N-flag-lacZ*	*	Da-A121YN-fw	amyE-Fw2	pCH2125	Da-R120X-Rv	amyE-Fw2-rv	pCH2125
pSK236	*gfp-apdA(39-128)-A121I-flag-lacZ*	*	Da-A121IM-fw	amyE-Fw2	pCH2125	Da-R120X-Rv	amyE-Fw2-rv	pCH2125
pSK237	*gfp-apdA(39-128)-A121Y-flag-lacZ*	*	Da-A121YN-fw	amyE-Fw2	pCH2125	Da-R120X-Rv	amyE-Fw2-rv	pCH2125
pSK238	*gfp-apdA(39-128)-P122N-flag-lacZ*	*	Da-P122X-Fw	amyE-Fw2	pCH2125	Da-P122X-Fw	amyE-Fw2-rv	pCH2125
pSK239	*gfp-apdA(39-128)-P122T-flag-lacZ*	*	Da-P122X-Fw	amyE-Fw2	pCH2125	Da-P122X-Fw	amyE-Fw2-rv	pCH2125
pSK241	*gfp-apdA(39-128)-R120N-flag-lacZ*	*	Da-R120N-Fw	amyE-Fw2	pCH2125	Da-R120X-Rv	amyE-Fw2-rv	pCH2125
pSK242	*gfp-apdA(39-128)-P122C-flag-lacZ*	*	Da-P122C-Fw	amyE-Fw2	pCH2125	Da-R120X-Rv	amyE-Fw2-rv	pCH2125
pSK243	*gfp-apdA(39-128)-P122Y-flag-lacZ*	*	Da-P122HY-Fw	amyE-Fw2	pCH2125	Da-R120X-Rv	amyE-Fw2-rv	pCH2125
pSK244	*gfp-apdA(39-128)-P122H-flag-lacZ*	*	Da-P122HY-Fw	amyE-Fw2	pCH2125	Da-R120X-Rv	amyE-Fw2-rv	pCH2125
pSK245	*gfp-apdA(39-128)-P122K-flag-lacZ*	*	Da-P122K-Fw	amyE-Fw2	pCH2125	Da-R120X-Rv	amyE-Fw2-rv	pCH2125
pSK246	*gfp-apdA(39-128)-P122I-flag-lacZ*	*	Da-P122I-fw2	amyE-Fw2	pCH2125	Da-R120X-Rv	amyE-Fw2-rv	pCH2125
pSK247	*gfp-apdP(34-140)-R131M-flag-lacZ*	*	Db-R131X-Fw	amyE-Fw2	pSK92	Db-R131X-Rv	amyE-Fw2-rv	pSK92
pSK248	*gfp-apdP(34-140)-R131V-flag-lacZ*	*	Db-R131X-Fw	amyE-Fw2	pSK92	Db-R131X-Rv	amyE-Fw2-rv	pSK92
pSK249	*gfp-apdP(34-140)-R131G-flag-lacZ*	*	Db-R131X-Fw	amyE-Fw2	pSK92	Db-R131X-Rv	amyE-Fw2-rv	pSK92
pSK250	*gfp-apdP(34-140)-R131I-flag-lacZ*	*	Db-R131X-Fw	amyE-Fw2	pSK92	Db-R131X-Rv	amyE-Fw2-rv	pSK92
pSK251	*gfp-apdP(34-140)-R131E-flag-lacZ*	*	Db-R131X-Fw	amyE-Fw2	pSK92	Db-R131X-Rv	amyE-Fw2-rv	pSK92
pSK252	*gfp-apdP(34-140)-R131K-flag-lacZ*	*	Db-R131X-Fw	amyE-Fw2	pSK92	Db-R131X-Rv	amyE-Fw2-rv	pSK92
pSK253	*gfp-apdP(34-140)-R131W-flag-lacZ*	*	Db-R131X-Fw	amyE-Fw2	pSK92	Db-R131X-Rv	amyE-Fw2-rv	pSK92
pSK254	*gfp-apdP(34-140)-R131D-flag-lacZ*	*	Db-R131X-Fw	amyE-Fw2	pSK92	Db-R131X-Rv	amyE-Fw2-rv	pSK92
pSK255	*gfp-apdP(34-140)-R131S-flag-lacZ*	*	Db-R131X-Fw	amyE-Fw2	pSK92	Db-R131X-Rv	amyE-Fw2-rv	pSK92
pSK256	*gfp-apdP(34-140)-R131L-flag-lacZ*	*	Db-R131X-Fw	amyE-Fw2	pSK92	Db-R131X-Rv	amyE-Fw2-rv	pSK92
pSK257	*gfp-apdP(34-140)-A132V-flag-lacZ*	*	Db-A132X-Fw	amyE-Fw2	pSK92	Db-R131X-Rv	amyE-Fw2-rv	pSK92
pSK258	*gfp-apdP(34-140)-A132G-flag-lacZ*	*	Db-A132X-Fw	amyE-Fw2	pSK92	Db-R131X-Rv	amyE-Fw2-rv	pSK92
pSK259	*gfp-apdP(34-140)-A132W-flag-lacZ*	*	Db-A132X-Fw	amyE-Fw2	pSK92	Db-R131X-Rv	amyE-Fw2-rv	pSK92
pSK260	*gfp-apdP(34-140)-P133M-flag-lacZ*	*	Db-P133X-Fw	amyE-Fw2	pSK92	Db-R131X-Rv	amyE-Fw2-rv	pSK92
pSK261	*gfp-apdP(34-140)-P133I-flag-lacZ*	*	Db-P133X-Fw	amyE-Fw2	pSK92	Db-R131X-Rv	amyE-Fw2-rv	pSK92
pSK262	*gfp-apdP(34-140)-P133Y-flag-lacZ*	*	Db-P133X-Fw	amyE-Fw2	pSK92	Db-R131X-Rv	amyE-Fw2-rv	pSK92
pSK264	*gfp-apdP(34-140)-P133S-flag-lacZ*	*	Db-P133X-Fw	amyE-Fw2	pSK92	Db-R131X-Rv	amyE-Fw2-rv	pSK92
pSK265	*gfp-apdP(34-140)-R131C-flag-lacZ*	*	Db-R131X-Fw	amyE-Fw2	pSK92	Db-R131X-Rv	amyE-Fw2-rv	pSK92
pSK266	*gfp-apdP(34-140)-R131N-flag-lacZ*	*	Db-R131X-Fw	amyE-Fw2	pSK92	Db-R131X-Rv	amyE-Fw2-rv	pSK92
pSK267	*gfp-apdP(34-140)-R131Y-flag-lacZ*	*	Db-R131X-Fw	amyE-Fw2	pSK92	Db-R131X-Rv	amyE-Fw2-rv	pSK92
pSK268	*gfp-apdP(34-140)-R131H-flag-lacZ*	*	Db-R131X-Fw	amyE-Fw2	pSK92	Db-R131X-Rv	amyE-Fw2-rv	pSK92
pSK269	*gfp-apdP(34-140)-R131P-flag-lacZ*	*	Db-R131X-Fw	amyE-Fw2	pSK92	Db-R131X-Rv	amyE-Fw2-rv	pSK92
pSK270	*gfp-apdP(34-140)-A132K-flag-lacZ*	*	Db-A132X-Fw	amyE-Fw2	pSK92	Db-R131X-Rv	amyE-Fw2-rv	pSK92
pSK271	*gfp-apdP(34-140)-P133V-flag-lacZ*	*	Db-P133X-Fw	amyE-Fw2	pSK92	Db-R131X-Rv	amyE-Fw2-rv	pSK92
pSK272	*gfp-apdP(34-140)-P133H-flag-lacZ*	*	Db-P133X-Fw	amyE-Fw2	pSK92	Db-R131X-Rv	amyE-Fw2-rv	pSK92
pSK273	*gfp-apdP(34-140)-P133L-flag-lacZ*	*	Db-P133X-Fw	amyE-Fw2	pSK92	Db-R131X-Rv	amyE-Fw2-rv	pSK92
pSK274	*gfp-apdP(34-140)-R131Q-flag-lacZ*	*	Db-R131X-Fw	amyE-Fw2	pSK92	Db-R131X-Rv	amyE-Fw2-rv	pSK92
pSK275	*gfp-apdP(34-140)-P133C-flag-lacZ*	*	Db-P133X-Fw	amyE-Fw2	pSK92	Db-R131X-Rv	amyE-Fw2-rv	pSK92
pSK276	*gfp-apdP(34-140)-P133Q-flag-lacZ*	*	Db-P133X-Fw	amyE-Fw2	pSK92	Db-R131X-Rv	amyE-Fw2-rv	pSK92
pSK277	*gfp-apdP(34-140)-P133W-flag-lacZ*	*	Db-P133X-Fw	amyE-Fw2	pSK92	Db-R131X-Rv	amyE-Fw2-rv	pSK92
pSK278	*gfp-apdP(34-140)-P133F-flag-lacZ*	*	Db-P133X-Fw	amyE-Fw2	pSK92	Db-R131X-Rv	amyE-Fw2-rv	pSK92
pSK281	*gfp-apdP(34-140)-A132D-flag-lacZ*	*	Db-A132X-Fw	amyE-Fw2	pSK92	Db-R131X-Rv	amyE-Fw2-rv	pSK92
pSK282	*gfp-apdP(34-140)-A132F-flag-lacZ*	*	Db-A132X-Fw	amyE-Fw2	pSK92	Db-R131X-Rv	amyE-Fw2-rv	pSK92
pSK283	*gfp-apdP(34-140)-A132E-flag-lacZ*	*	Db-A132X-Fw	amyE-Fw2	pSK92	Db-R131X-Rv	amyE-Fw2-rv	pSK92
pSK284	*gfp-apdP(34-140)-A132T-flag-lacZ*	*	Db-A132X-Fw	amyE-Fw2	pSK92	Db-R131X-Rv	amyE-Fw2-rv	pSK92
pSK285	*gfp-apdP(34-140)-A132Q-flag-lacZ*	*	Db-A132X-Fw	amyE-Fw2	pSK92	Db-R131X-Rv	amyE-Fw2-rv	pSK92
pSK292	*gfp-apdP(34-140)-A132L-flag-lacZ*	*	Db-A132X-Fw	amyE-Fw2	pSK92	Db-R131X-Rv	amyE-Fw2-rv	pSK92
pSK297	*gfp-apdP(34-140)-A132M-flag-lacZ*	*	Db-A132IM-fw	amyE-Fw2	pSK92	Db-R131X-Rv	amyE-Fw2-rv	pSK92
pSK298	*gfp-apdP(34-140)-A132C-flag-lacZ*	*	Db-A132YC-fw	amyE-Fw2	pSK92	Db-R131X-Rv	amyE-Fw2-rv	pSK92
pSK299	*gfp-apdP(34-140)-A132N-flag-lacZ*	*	Db-A132N-w	amyE-Fw2	pSK92	Db-R131X-Rv	amyE-Fw2-rv	pSK92
pSK300	*gfp-apdP(34-140)-R131F-flag-lacZ*	*	Db-R131F-fw	amyE-Fw2	pSK92	Db-R131X-Rv	amyE-Fw2-rv	pSK92
pSK301	*gfp-apdP(34-140)-R131T-flag-lacZ*	*	Db-R131T-fw	amyE-Fw2	pSK92	Db-R131X-Rv	amyE-Fw2-rv	pSK92
pSK302	*gfp-apdP(34-140)-P133T-flag-lacZ*	*	Db-P133T-fw	amyE-Fw2	pSK92	Db-R131X-Rv	amyE-Fw2-rv	pSK92
pSK303	*gfp-apdP(34-140)-P133R-flag-lacZ*	*	Db-P133R-Fw	amyE-Fw2	pSK92	Db-R131X-Rv	amyE-Fw2-rv	pSK92
pSK304	*gfp-apdP(34-140)-P133E-flag-lacZ*	*	Db-P133DE-fw	amyE-Fw2	pSK92	Db-R131X-Rv	amyE-Fw2-rv	pSK92
pSK305	*gfp-apdP(34-140)-P133D-flag-lacZ*	*	Db-P133DE-fw	amyE-Fw2	pSK92	Db-R131X-Rv	amyE-Fw2-rv	pSK92
pSK306	*gfp-apdP(34-140)-P133N-flag-lacZ*	*	Db-P133KN-fw	amyE-Fw2	pSK92	Db-R131X-Rv	amyE-Fw2-rv	pSK92
pSK307	*gfp-apdP(34-140)-A132I-flag-lacZ*	*	Db-A132IM-fw	amyE-Fw2	pSK92	Db-R131X-Rv	amyE-Fw2-rv	pSK92
pSK308	*gfp-apdP(34-140)-A132P-flag-lacZ*	*	Db-A132PRH-fw	amyE-Fw2	pSK92	Db-R131X-Rv	amyE-Fw2-rv	pSK92
pSK309	*gfp-apdP(34-140)-A132R-flag-lacZ*	*	Db-A132PRH-fw	amyE-Fw2	pSK92	Db-R131X-Rv	amyE-Fw2-rv	pSK92
pSK310	*gfp-apdP(34-140)-P133K-flag-lacZ*	*	Db-P133KN-fw	amyE-Fw2	pSK92	Db-R131X-Rv	amyE-Fw2-rv	pSK92
pSK311	*gfp-apdP(34-140)-A132H-flag-lacZ*	*	Db-A132PRH-fw	amyE-Fw2	pSK92	Db-R131X-Rv	amyE-Fw2-rv	pSK92
pSK312	*gfp-apdP(34-140)-A132Y-flag-lacZ*	*	Db-A132YC-fw	amyE-Fw2	pSK92	Db-R131X-Rv	amyE-Fw2-rv	pSK92
pKIG1246	*gfp-apdP(34-103)-5P-myc-lacZ*	*	myc-lacZ7-CTC-fw	LacZ60_stp_amyEf rv	pSK69	LacZ60_stp_amyEf fw	LapdP-5P-myc rv	pSK69
pKIG1257	*gfp-apdP(34-103)-5A-myc-lacZ*	*	ampR 121-128(Tm62)	RQH_vec rv	pSK69	LapdP-5A-myc fw	ampR 121-128(Tm62).antisense	pKIG1245
pKIG1427	*gfp-secM(38-170)-G165P-myc-lacZ*	(4)						
pKIG1429	*gfp-secM(38-170)-G165P/P166G-myc-lacZ*	*	ampR 121-128(Tm62)	secM_Ile163 rv	pCH2421	secM_RAPG fw	ampR 121-128(Tm62).antisense	pCH2421
pKIG1431	*gfp-secM(38-170)-A164P/G165P-myc-lacZ*	*	ampR 121-128(Tm62)	secM_Ile163 rv	pCH2421	secM_RGPP fw	ampR 121-128(Tm62).antisense	pCH2421
pKIG1433	*gfp-secM(38-170)-R163A/G165P-myc-lacZ*	(4)						
pKIG1435	*gfp-ApdP(34-140)-P133G-myc-lacZWcat*	*	ampR 121-128(Tm62)	Db-R131X-Rv	pCH2429	Dp_RAGP fw	ampR 121-128(Tm62).antisense	pCH2429
pKIG1439	*gfp-ApdP(34-140)-P134G-myc-lacZWcat*	*	ampR 121-128(Tm62)	Db-R131X-Rv	pCH2429	Dp_RAPG fw	ampR 121-128(Tm62).antisense	pCH2429
pKIG1441	*gfp-ApdP(34-140)-A132G-myc-lacZWcat*	*	ampR 121-128(Tm62)	Db-R131X-Rv	pCH2429	Dp_RGPP fw	ampR 121-128(Tm62).antisense	pCH2429
pKIG1443	*gfp-ApdP(34-140)-R131A/P133G-myc-lacZWcat*	*	ampR 121-128(Tm62)	Db-R131X-Rv	pCH2429	Dp_AAGP fw	ampR 121-128(Tm62).antisense	pCH2429
pSTV28	*vector*	Takara						
pNH106	*Plac rplV+*	(5)						
pNH112	*Plac rplV(dM96K97R98)*	(5)						
pTJ83	*gfp-SLIV_07330(28-129)-myc-lacZ*	*	gfp238-Sl_apdA28-fw	Sl_apdA-myc27-rv2	S lividans chrDN	gfp238-rv	myc-lacZ7-fw	pSK69
pTJ86	*gfp-SLIV_07330(28-129)R121A-myc-lacZ*	*	Sl_Da-R121A-fw2	ampR 121-128(Tm62).antisense	pTJ83	Sl_Da-R121A-rv2	ampR 121-128(Tm62)	pTJ83
pTJ101	*gfp-SLIV_16130(29-118)-myc-lacZ*	*	302.gfp-anotherRAPP28.fw	303.anotherRAPP117-myc.rv	S lividans chrDN	304.anotherRAPP117-myc.f	305.gfp-anotherRAPP28.rv(myc.fw	pSK69
pTJ105	*gfp-SLIV_16130(29-118)R105A-myc-lacZ*	*	327.16130R105A.fw(H)	ampR 121-128(Tm62).antisense	pTJ101	328.16130R105A.rv(H)	ampR 121-128(Tm62)	pTJ101
pTJ109	*gfp-SLIV_18480(213-284)-myc-lacZ*	*	340.gfp-18480.fw	341.18480-myc.rv	S lividans chrDN	gfp238-rv	myc-lacZ7-fw	pSK69
pTJ269	*gfp-SLIV_18480(213-284)R226A-myc-lacZ*	*	735.SL18480RA.fw	ampR 121-128(Tm62).antisense	pTJ109	736.SL18480RA.rv	ampR 121-128(Tm62)	pTJ109
pTJ108	*gfp-SLIV_33595(169-234)-myc-lacZ*	*	342.gfp-33595.fw	343.33595-myc.rv	S lividans chrDN	gfp238-rv	myc-lacZ7-fw	pSK69
pTJ270	*gfp-SLIV_33595(169-234)R225A-myc-lacZ*	*	737.SL33595RA.fw	ampR 121-128(Tm62).antisense	pTJ108	738.SL33595RA.rv	ampR 121-128(Tm62)	pTJ108
pTJ110	*gfp-SLIV_33605(191-256)-myc-lacZ*	*	344.gfp-33605.fw	355.33605-myc.rv	S lividans chrDN	gfp238-rv	myc-lacZ7-fw	pSK69
pTJ275	*gfp-SLIV_33605(191-256)R247A-myc-lacZ*	*	739.SL33605RA.fw	ampR 121-128(Tm62).antisense	pTJ110	740.SL33605RA.rv	ampR 121-128(Tm62)	pTJ110
pTJ321	*gfp-SLIV_27375(196-236)-myc-lacZ*	*	550.27375.fw2	521.27375.rv	S lividans chrDN	gfp238-rv	myc-lacZ7_CTC-fw	pSK69
pTJ345	*gfp-SLIV_27375(196-236)R226A-myc-lacZ*	*	741.SL27375RA.fw	ampR 121-128(Tm62).antisense	pTJ321	742.SL27375RA.rv	ampR 121-128(Tm62)	pTJ321
pTJ322	*gfp-SLIV_32905(42-81)-myc-lacZ*	*	522.32905.fw	523.32905.rv	S lividans chrDN	gfp238-rv	myc-lacZ7_CTC-fw	pSK69
pTJ344	*gfp-SLIV_32905(42-81)R72A-myc-lacZ*	*	743.SL32905RA.fw	ampR 121-128(Tm62).antisense	pTJ322	744.SL32905RA.rv	ampR 121-128(Tm62)	pTJ322

(1) Sakiyama.,at al. (2021) *Nucleic Acids Res.*, **49**, 1550–1566.
(2) Fujiwara et al. (2024) *Nat. Commun.*, **15**, 2711.
(3) Morici et al. (2024) *Nat. Commun.,* **15**, 2432.
(4) Gersteuer et al. (2024) *Nat. Commun.*, **15**, 2431.
(5) Nakatogawa et al., (2002) *Cell*, **108**, 629-636.
*This study

Appendix Table S4: Primers

Primer name	sequence (5' to 3')
Aj_Da-myc27-rv	ATCCTCTTCTGAGATGAGTTTTTGTTCGACGGCTACCGGGAAAGGAGG
ampR 121-128(Tm62)	GCAGTGCTGCCATAACCATGAGTG
ampR 121-128(Tm62)-antisense	CACTCATGGTTATGGCAGCACTGC
amyE-Fw2	ACGCGGTCATCAATCATACC
amyE-Fw2-rv	GGTATGATTGATGACCGCGT
Ca-A103K-Fw	CCCGGCCGTCCACAGCCTCGAAAACCGGGAATCGGTCTGGACTAT
Ca-A103M-Fw	CCCGGCCGTCCACAGCCTCGAATGCCGGGAATCGGTCTGGACTAT
Ca-A103N-Fw	CCCGGCCGTCCACAGCCTCGAAATCCGGGAATCGGTCTGGACTAT
Ca-A103Q-Fw	CCCGGCCGTCCACAGCCTCGACAGCCGGGAATCGGTCTGGACTAT
Ca-A103S-fw	CCCGGCCGTCCACAGCCTCGATCTCCGGGAATCGGTCTGGACTAT
Ca-A103S-rv	TCGAGGCTGTGGACGGCCGGG
Ca-A103X-fw	CCCGGCCGTCCACAGCCTCGANNNCCGGGAATCGGTCTGGACTAT
Ca-P104A-fw	GGCCGTCCACAGCCTCGAGCTGCGGGAATCGGTCTGGACTATAAA
Ca-P104A-rv	AGCTCGAGGCTGTGGACGGCC
Ca-P104C-fw	GGCCGTCCACAGCCTCGAGCTTGCGGAATCGGTCTGGACTATAAA
Ca-P104F-fw	GGCCGTCCACAGCCTCGAGCTTTCGGAATCGGTCTGGACTATAAA
Ca-P104G-fw	GGCCGTCCACAGCCTCGAGCTGGGGGAATCGGTCTGGACTATAAA
Ca-P104H-fw	GGCCGTCCACAGCCTCGAGCTCATGGAATCGGTCTGGACTATAAA
Ca-P104Q-fw	GGCCGTCCACAGCCTCGAGCTCAGGGAATCGGTCTGGACTATAAA
Ca-P104T-fw	GGCCGTCCACAGCCTCGAGCTACGGGAATCGGTCTGGACTATAAA
Ca-P104X-fw	GGCCGTCCACAGCCTCGAGCTNNNGGAATCGGTCTGGACTATAAA
Ca-R102A-fw	GCGCCCGGCCGTCCACAGCCTGCAGCTCCGGGAATCGGTCTGGAC
Ca-R102A-rv	AGGCTGTGGACGGCCGGGCGC
Ca-R102E-Fw	GCGCCCGGCCGTCCACAGCCTGAAGCTCCGGGAATCGGTCTGGAC
Ca-R102V-Fw	GCGCCCGGCCGTCCACAGCCTGTGGCTCCGGGAATCGGTCTGGAC
Ca-R102X-fw	GCGCCCGGCCGTCCACAGCCTNNNGCTCCGGGAATCGGTCTGGAC
Ca-R102X-Fw2	GCGCCCGGCCGTCCACAGCCTDDSGCTCCGGGAATCGGTCTGGAC
Ca-R102Y-Fw	GCGCCCGGCCGTCCACAGCCTTATGCTCCGGGAATCGGTCTGGAC
Da-A121F-fw	CGCCTCGCGCTGGGTGATCGCTTCCCTCCTTTCCCGGTAGCCGTC
Da-A121G-fw	CGCCTCGCGCTGGGTGATCGCGGCCCTCCTTTCCCGGTAGCCGTC
Da-A121HQP-fw	CGCCTCGCGCTGGGTGATCGCCMSCCTCCTTTCCCGGTAGCCGTC
Da-A121IM-fw	CGCCTCGCGCTGGGTGATCGCATNCCTCCTTTCCCGGTAGCCGTC
Da-A121S-fw	CGCCTCGCGCTGGGTGATCGCTCTCCTCCTTTCCCGGTAGCCGTC
Da-A121S-rv	GCGATCACCCAGCGCGAGGCG
Da-A121W-fw	CGCCTCGCGCTGGGTGATCGCTGGCCTCCTTTCCCGGTAGCCGTC
Da-A121X-Fw	CGCCTCGCGCTGGGTGATCGCNNNCCTCCTTTCCCGGTAGCCGTC
Da-A121X-Rv	GCGATCACCCAGCGCGAGGCG
Da-A121YN-fw	CGCCTCGCGCTGGGTGATCGCWACCCTCCTTTCCCGGTAGCCGTC
Da-P122A-fw	CTCGCGCTGGGTGATCGCGCTGCTCCTTTCCCGGTAGCCGTCGAC
Da-P122A-rv	AGCGCGATCACCCAGCGCGAG
Da-P122C-Fw	CTCGCGCTGGGTGATCGCGCTTGTCCTTTCCCGGTAGCCGTCGAC
Da-P122G-fw	CAGAGCAAGTGCATTCGCGCGGGGCCAGCGGCGGGAGCCTTCCTT
Da-P122HY-Fw	CTCGCGCTGGGTGATCGCGCTYATCCTTTCCCGGTAGCCGTCGAC
Da-P122I-fw2	CTCGCGCTGGGTGATCGCGCTATCCCTTTCCCGGTAGCCGTCGAC
Da-P122K-Fw	CTCGCGCTGGGTGATCGCGCTAAACCTTTCCCGGTAGCCGTCGAC
Da-P122X-Fw	CTCGCGCTGGGTGATCGCGCTNNNCCTTTCCCGGTAGCCGTCGAC
Da-P122X-Rv	AGCGCGATCACCCAGCGCGAG
Da-R120D-Fw	AACCGCCTCGCGCTGGGTGATGACGCTCCTCCTTTCCCGGTAGCC
Da-R120F-Fw	AACCGCCTCGCGCTGGGTGATTTCGCTCCTCCTTTCCCGGTAGCC
Da-R120H-Fw	AACCGCCTCGCGCTGGGTGATCATGCTCCTCCTTTCCCGGTAGCC
Da-R120N-Fw	AACCGCCTCGCGCTGGGTGATAACGCTCCTCCTTTCCCGGTAGCC
Da-R120X-Fw	AACCGCCTCGCGCTGGGTGATNNNGCTCCTCCTTTCCCGGTAGCC
Da-R120X-Fw2	AACCGCCTCGCGCTGGGTGATADCGCTCCTCCTTTCCCGGTAGCC
Da-R120X-Rv	ATCACCCAGCGCGAGGCGGTT
Da104-Dp116-fw	AGCGAGCTCCACGACGAAAAAACCAGAATTGCGCGTCTC
Da104-rv	TTCGTCGTGGAGCTCGCTGAG
Da109-Dp121-fw	GAAGCAGCTCCCGCCTCGCGTCTCCCATCCTGTCAGAGC
Da109-rv	CGAGGCGGGAGCTGCTTCGTC
Da114-Dp126-fw	TCGCGCACGGCGAACCGCCAGAGCAAGTGCATTCGCGCG
Da114-rv	GCGGTTCGCCGTGCGCGAGGC
Da119-Dp131-fw	CGCCTCGCGCTGGGTGATCGCGCGCCGCCAGCGGCGGGA
Da119-rv	ATCACCCAGCGCGAGGCGGTT
Db-A132IM-fw	TGTCAGAGCAAGTGCATTCGCATKCCGCCAGCGGCGGGAGCCTTC
Db-A132N-w	TGTCAGAGCAAGTGCATTCGCAACCCGCCAGCGGCGGGAGCCTTC
Db-A132PRH-fw	TGTCAGAGCAAGTGCATTCGCCVTCCGCCAGCGGCGGGAGCCTTC
Db-A132S-fw	TGTCAGAGCAAGTGCATTCGCTCGCCGCCAGCGGCGGGAGCCTTC
Db-A132S-rv	GCGAATGCACTTGCTCTGACA
Db-A132X-Fw	TGTCAGAGCAAGTGCATTCGCNNNCCGCCAGCGGCGGGAGCCTTC
Db-A132YC-fw	TGTCAGAGCAAGTGCATTCGCTRTCCGCCAGCGGCGGGAGCCTTC
Db-lacZ1-9del-fw	TTAAAAACATATCAAACTAGTGTCGTTTTACAACGTCGTGAC
Db-lacZ1-9del-rv	ACTAGTTTGATATGTTTTTAA
Db-P133A-fw	CAGAGCAAGTGCATTCGCGCGGCGCCAGCGGCGGGAGCCTTCCTT
Db-P133A-rv	CGCGCGAATGCACTTGCTCTG
Db-P133DE-fw	CAGAGCAAGTGCATTCGCGCGGAMCCAGCGGCGGGAGCCTTCCTT
Db-P133G-fw	CAGAGCAAGTGCATTCGCGCGGGGCCAGCGGCGGGAGCCTTCCTT
Db-P133KN-fw	CAGAGCAAGTGCATTCGCGCGAAMCCAGCGGCGGGAGCCTTCCTT
Db-P133R-Fw	CAGAGCAAGTGCATTCGCGCGCGCCCAGCGGCGGGAGCCTTCCTT
Db-P133T-fw	CAGAGCAAGTGCATTCGCGCGACCCCAGCGGCGGGAGCCTTCCTT
Db-P133X-Fw	CAGAGCAAGTGCATTCGCGCGNNNCCAGCGGCGGGAGCCTTCCTT
Db-R131F-fw	TCCTGTCAGAGCAAGTGCATTTTCGCGCCGCCAGCGGCGGGAGCC
Db-R131T-fw	TCCTGTCAGAGCAAGTGCATTACCGCGCCGCCAGCGGCGGGAGCC
Db-R131X-Fw	TCCTGTCAGAGCAAGTGCATTNNNGCGCCGCCAGCGGCGGGAGCC

Db-R131X-Rv	AATGCACTTGCTCTGACAGGA
Dp115-Da105-fw	CCTTCCCTGCCGGTGGCGGCAGCTCCCGCCTCGCGCACG
Dp115-rv	CGCCACCGGCAGGGAAGGAGC
Dp120-Da110-fw	GCGAAAACCAGAATTGCGCGCACGGCGAACCGCCTCGCG
Dp120-rv	CGCAATTCTGGTTTTCGCCAC
Dp125-Da115-fw	GCGCGTCTCCCATCCTGTCTCGCGCTGGGTGATCGCGCT
Dp125-rv	ACAGGATGGGAGACGCGCAAT
Dp130-Da120-fw	TGTCAGAGCAAGTGCATTCGCGCTCCTCCTTTCCCGGTA
Dp130-rv	AATGCACTTGCTCTGACAGGA
gfp238-fw	GGCATGGATGAACTATACAAA
gfp238-mifM35-fw2	GGCATGGATGAACTATACAAACGGGAGTGCCGTGCGGGCGAG
GFP238-myc-rv	ATCCTCTTCTGAGATGAGTTTTTGTTCTTTGTATAGTTCATCCATGCC
GFP238-rv	TTTGTATAGTTCATCCATGCC
lacZ60-TAATAA-21-rv	TGGTGCCGGAAACCAGGCAAATTATTAGCGCCATTCGCCATTCAGGCT
mifM-myc27-rv	ATCCTCTTCTGAGATGAGTTTTTGTTCTAAAAGAAGAGAACCGGCGTC
myc-lacZ7-fw	AAACTCATCTCAGAAGAGGATCTGTCACTGGCCGTCGTTTTACAA
pCH2126-del-ori-fw	TGGGGAAAAAATCATCGACCTCGAGCTGGATACTTCCCG
pMW118-ori-fw	GACAGTAAGACGGGTAAGCCTGTT
pMW118-ori-rv	ATGATTTTTTCCCCACGGGAGGCG
PT7-RBSkf-GFP	TAACTTTAAGAAGGAGGGAGATATACCAATGACAATGTTTGTGGGATC
Re_Ca-myc27-rv	ATCCTCTTCTGAGATGAGTTTTTGTTCCAGACCGATTCCCGGAGC
Sinor medic-Db-R131A-fw	AAGTGCATTGCCGCGCCGCCA
Sinor medic-Db-R131A-rv	CGCGGCAATGCACTTGCTCTGACAGGA
Sm_Db-myc27-rv	ATCCTCTTCTGAGATGAGTTTTTGTTCAAGGAAGGCTCCCGCCGCTGG
Universal-primer-77(PURE)	GAAATTAATACGACTCACTATAGGGAGACCACAACGGTTTCCCTCTAGAAATAATTTTGTTTAACTTTAAGAAGGAG
myc-lacZ7-CTC-fw	AAACTCATCTCAGAAGAGGATCTCTCACTCGCCGTCGTTTTACAA
myc27-rv	ATCCTCTTCTGAGATGAGTTTTTGTTC
LacZ60_stp_amyEf rv	GCCGTTATTAGCGCCATTCGCCATTCAGGC
RQH_vec rv	CAGGGCAATGGCCTGGAAAG
LacZ60_stp_amyEf fw	CGAATGGCGCTAATAACGGCCGTAATAGAG
LapdP-5A-myc fw	CTTTCCAGGCCATTGCCCTGGCAGCTGCCGCTGCAGAACAAAAACTCATCTCAGAAGAGGAT
LapdP-5P-myc rv	ATCCTCTTCTGAGATGAGTTTTTGTTCAGGTGGCGGTGGAGGCAGGGCAATGGCCTGGAAAG
LapdP-10P-myc rv	ATCCTCTTCTGAGATGAGTTTTTGTTCAGGTGGCGGTGGAGGAGGTGGCGGTGGAGGCAGGGCAATGGCCTGGAAAG
Re_apcA-G105P-myc-fw	CCACAGCCTCGAGCTCCGCCAATCGGTCTGGAACAAAAA
Re_apcA-P104-rv	CGGAGCTCGAGGCTGTGGACG
ApcA_RGPP-myc-fw	GGCCGTCCACAGCCTCGAGGTCCGCCAATCGGTCTGGAA
Re_ApdA_R102-rv	TCGAGGCTGTGGACGGCCGGG
ApcA_RAGP-myc-fw	CGTCCACAGCCTCGAGCTGGACCAATCGGTCTGGAACAAAAA
Re_ApdA_A103-rv	AGCTCGAGGCTGTGGACGGCC
ApcA_AAPP-myc-fw	CCCGGCCGTCCACAGCCTGCAGCTCCGCCAATCGGTCTGGAACAAAAA
Re_ApdA_P101-rv	AGGCTGTGGACGGCCGGGCGC
pTWV229-AmpR-TAA-fw	CTGTCAGACCAAGTTTACTCA
pTWV229-AmpR-SD-rv	TTTCAATATTATTGAAGCATTTATCAG
pTWV228-cat-SD-fw	AATGCTTCAATAATATTGAAAAGATTTTCAGGAGCTAAGGAA
pTWV228-cat-rv	GTAAACTTGGTCTGACAGTTACGCCCCGCCCTGCCA
secM_Ile163 rv	GATGCCTTGCGCCTGGCTTAT
secM_RAPG fw	AGCCAGGCGCAAGGCATCCGTGCTCCTGGACAACGCCTCACCGAA
secM_RGPP fw	AGCCAGGCGCAAGGCATCCGTGGTCCTCCTCAACGCCTCACCGAA
Db-R131X-Rv	AATGCACTTGCTCTGACAGGA
Dp_RAGP fw	TGTCAGAGCAAGTGCATTCGCGCGGGACCAGCGGCGGGAGCCTTC
Dp_RAPG fw	TGTCAGAGCAAGTGCATTCGCGCGCCGGGAGCGGCGGGAGCCTTC
Dp_RGPP fw	TGTCAGAGCAAGTGCATTCGCGGGCCGCCAGCGGCGGGAGCCTTC
Dp_AAGP fw	TGTCAGAGCAAGTGCATTGCCGCGGGACCAGCGGCGGGAGCCTTC
gfp238-Sl_apdA28-fw	GGCATGGATGAACTATACAAAGCGCACGTCGGTCCGAGC
Sl_apdA-myc27-rv2	ATCCTCTTCTGAGATGAGTTTTTGTTCGGTGCCGGAGGAGACCGGTGG
Sl_Da-R121A-fw2	GACAGCGGCGCGGCGCCACCGGTCTCC
Sl_Da-R121A-rv2	TGGCGCCGCGCCGCTGTCGTGGCTCGCGCG
302.gfp-anotherRAPP28.fw	ACTATACAAAGGTGCCGGTCACGGGACGGTGATGA
303.anotherRAPP117-myc.rv	GTTTTTGTTCTATCCGCAGGAGCTGCAACTCGGCG
304.anotherRAPP117-myc.fw(gfprv	GACCGGCACCTTTGTATAGTTCATCCATGCCATGT
305.gfp-anotherRAPP28.rv(myc.fw	CCTGCGGATAGAACAAAAACTCATCTCAGAAGAGG
327.16130R105A.fw(H)	CACGACGGCGCGGCTCCGCCCGAC
328.16130R105A.rv(H)	GTCGGGCGGAGCCGCGCCGTCGTG
340.gfp-18480.fw	CATGGCATGGATGAACTATACAAATCGGCCCAGTCCATGGCCGAAGC
341.18480-myc.rv	CCTCTTCTGAGATGAGTTTTTGTTCGGCGGCGAGGGCGAACGCGTCACT
735.SL18480RA.fw	ACACCGTCATCAGGGCCGGCCCGCCGAGTGACGCGTTC
736.SL18480RA.rv	GAACGCGTCACTCGGCGGGCCGGCCCTGATGACGGTGT
342.gfp-33595.fw	CATGGCATGGATGAACTATACAAAGAACGCGCGACCTTCCGCGTTCTGC
343.33595-myc.rv	CCTCTTCTGAGATGAGTTTTTGTTCGACGACAGCGGTCCCTTCAGGCGG
737.SL33595RA.fw	GTGATCACTTCTGCGGGGCCGCCTGAAGGGACCGCT
738.SL33595RA.rv	AGCGGTCCCTTCAGGCGGCCCCGCAGAAGTGATCAC
344.gfp-33605.fw	CATGGCATGGATGAACTATACAAAGAGAGCGCGCTCTTCAAGACGGTCC
355.33605-myc.rv	CCTCTTCTGAGATGAGTTTTTGTTCGACGACAGCGGTCTCCCCGGGAGG
739.SL33605RA.fw	CGTCCTCACCACCGCGGGCCCTCCCGGGGAGACCGC
740.SL33605RA.rv	GCGGTCTCCCCGGGAGGGCCCGCGGTGGTGAGGACG
520.27375.fw	GCATGGATGAACTATACAAACGCCGGGCCCCGGCGGTGCG
521.27375.rv	TCTGAGATGAGTTTTTGTTCGACGAGGGTGGCCACCGGGC
741.SL27375RA.fw	CTCCCTGGGACGTGCCGGACCGCCGTGCCCGGTGGCCAC
742.SL27375RA.rv	GTGGCCACCGGGCACGGCGGTCCGGCACGTCCCAGGGAG
522.32905.fw	GCATGGATGAACTATACAAATTGCACCAGGTGCGCACCTT
523.32905.rv	TCTGAGATGAGTTTTTGTTCAAACCGTCTGCTGTGACATG
743.SL32905RA.fw	GGGCGATCCTGCGGGACCGCCATGTCACAGCA
744.SL32905RA.rv	TGCTGTGACATGGCGGTCCCGCAGGATCGCCC

Appendix Table. 5: Templates for PURE system

gene	1st PCR			2nd PCR	
	fw primer 1	rv primer 1	template 1	fw primer 2	rv primer 2
gfp-apdP(34-103)-5P-lacZa	PT7-RBSkf-GFP	lacZ60-TAATAA-21-rv	pKIG1246	Universal-primer-77(PURE)	lacZ60-TAATAA-21-rv
gfp-apdP(34-103)-5A-lacZa	PT7-RBSkf-GFP	lacZ60-TAATAA-21-rv	pKIG1257	Universal-primer-77(PURE)	lacZ60-TAATAA-21-rv
gfp-apdA-lacZa	PT7-RBSkf-GFP	lacZ60-TAATAA-21-rv	pCH2427	Universal-primer-77(PURE)	lacZ60-TAATAA-21-rv
gfp-apdA(R120A)-lacZa	PT7-RBSkf-GFP	lacZ60-TAATAA-21-rv	pCH2428	Universal-primer-77(PURE)	lacZ60-TAATAA-21-rv
gfp-apdP-lacZa	PT7-RBSkf-GFP	lacZ60-TAATAA-21-rv	pCH2429	Universal-primer-77(PURE)	lacZ60-TAATAA-21-rv
gfp-apdP(R131A)-lacZa	PT7-RBSkf-GFP	lacZ60-TAATAA-21-rv	pCH2430	Universal-primer-77(PURE)	lacZ60-TAATAA-21-rv
gfp-apdP(34-140)-A132G-myc-lacZWcat	PT7-RBSkf-GFP	lacZ60-TAATAA-21-rv	pKIG1441	Universal-primer-77(PURE)	lacZ60-TAATAA-21-rv
gfp-apdP(34-140)-P134G-myc-lacZWcat	PT7-RBSkf-GFP	lacZ60-TAATAA-21-rv	pKIG1439	Universal-primer-77(PURE)	lacZ60-TAATAA-21-rv
gfp-apdP(34-140)-P133G-myc-lacZWcat	PT7-RBSkf-GFP	lacZ60-TAATAA-21-rv	pKIG1435	Universal-primer-77(PURE)	lacZ60-TAATAA-21-rv
gfp-apcA(62-108)-myc-lacZWcat	PT7-RBSkf-GFP	lacZ60-TAATAA-21-rv	pCH2425	Universal-primer-77(PURE)	lacZ60-TAATAA-21-rv
gfp-apcA(62-108)-G105P-myc-lacZWcat	PT7-RBSkf-GFP	lacZ60-TAATAA-21-rv	pCH2437	Universal-primer-77(PURE)	lacZ60-TAATAA-21-rv
gfp-apcA(62-108)-A103G/G105P(RGPP)-myc-lacZWcat	PT7-RBSkf-GFP	lacZ60-TAATAA-21-rv	pCH2599	Universal-primer-77(PURE)	lacZ60-TAATAA-21-rv
gfp-apcA(62-108)-R102A/G105P(AAPP)-myc-lacZWcat	PT7-RBSkf-GFP	lacZ60-TAATAA-21-rv	pCH2600	Universal-primer-77(PURE)	lacZ60-TAATAA-21-rv
gfp-apcA(62-108)-P104G/G105P(RAGP)-myc-lacZWcat	PT7-RBSkf-GFP	lacZ60-TAATAA-21-rv	pCH2601	Universal-primer-77(PURE)	lacZ60-TAATAA-21-rv
gfp-secM(38-170)-myc-lacZ	PT7-RBSkf-GFP	lacZ60-TAATAA-21-rv	pCH2421	Universal-primer-77(PURE)	lacZ60-TAATAA-21-rv
gfp-secM(38-170)-G165P-myc-lacZ	PT7-RBSkf-GFP	lacZ60-TAATAA-21-rv	pKIG1427	Universal-primer-77(PURE)	lacZ60-TAATAA-21-rv
gfp-secM(38-170)-R163A/G165P-myc-lacZ	PT7-RBSkf-GFP	lacZ60-TAATAA-21-rv	pKIG1433	Universal-primer-77(PURE)	lacZ60-TAATAA-21-rv
gfp-secM(38-170)-A164P/G165P-myc-lacZ	PT7-RBSkf-GFP	lacZ60-TAATAA-21-rv	pKIG1431	Universal-primer-77(PURE)	lacZ60-TAATAA-21-rv
gfp-secM(38-170)-G165P/P166G-myc-lacZ	PT7-RBSkf-GFP	lacZ60-TAATAA-21-rv	pKIG1429	Universal-primer-77(PURE)	lacZ60-TAATAA-21-rv
gfp-SLIV_07330(28-129)-myc-lacZ	PT7-RBSkf-GFP	lacZ-140Rv	pTJ83	Universal-primer-77(PURE)	lacZ60-TAATAA-21-rv
gfp-SLIV_07330(28-129)R121A-myc-lacZ	PT7-RBSkf-GFP	lacZ-140Rv	pTJ86	Universal-primer-77(PURE)	lacZ60-TAATAA-21-rv
gfp-SLIV_16130(29-118)-myc-lacZ	PT7-RBSkf-GFP	lacZ-140Rv	pTJ101	Universal-primer-77(PURE)	lacZ60-TAATAA-21-rv
gfp-SLIV_16130(29-118)R105A-myc-lacZ	PT7-RBSkf-GFP	lacZ-140Rv	pTJ105	Universal-primer-77(PURE)	lacZ60-TAATAA-21-rv
gfp-SLIV_18480(213-284)-myc-lacZ	PT7-RBSkf-GFP	lacZ-140Rv	pTJ109	Universal-primer-77(PURE)	lacZ60-TAATAA-21-rv
gfp-SLIV_18480(213-284)R226A-myc-lacZ	PT7-RBSkf-GFP	lacZ-140Rv	pTJ269	Universal-primer-77(PURE)	lacZ60-TAATAA-21-rv
gfp-SLIV_33595(169-234)-myc-lacZ	PT7-RBSkf-GFP	lacZ-140Rv	pTJ108	Universal-primer-77(PURE)	lacZ60-TAATAA-21-rv
gfp-SLIV_33595(169-234)R225A-myc-lacZ	PT7-RBSkf-GFP	lacZ-140Rv	pTJ270	Universal-primer-77(PURE)	lacZ60-TAATAA-21-rv
gfp-SLIV_33605(191-256)-myc-lacZ	PT7-RBSkf-GFP	lacZ-140Rv	pTJ110	Universal-primer-77(PURE)	lacZ60-TAATAA-21-rv
gfp-SLIV_33605(191-256)R247A-myc-lacZ	PT7-RBSkf-GFP	lacZ-140Rv	pTJ275	Universal-primer-77(PURE)	lacZ60-TAATAA-21-rv
gfp-SLIV_27375(196-236)-myc-lacZ	PT7-RBSkf-GFP	lacZ-140Rv	pTJ321	Universal-primer-77(PURE)	lacZ60-TAATAA-21-rv
gfp-SLIV_27375(196-236)R226A-myc-lacZ	PT7-RBSkf-GFP	lacZ-140Rv	pTJ345	Universal-primer-77(PURE)	lacZ60-TAATAA-21-rv
gfp-SLIV_32905(42-81)-myc-lacZ	PT7-RBSkf-GFP	lacZ-140Rv	pTJ322	Universal-primer-77(PURE)	lacZ60-TAATAA-21-rv
gfp-SLIV_32905(42-81)R72A-myc-lacZ	PT7-RBSkf-GFP	lacZ-140Rv	pTJ344	Universal-primer-77(PURE)	lacZ60-TAATAA-21-rv
