# Supplementary material for: Evolutionary adaptation of bacterial proteomes to translation-impeding sequences
Source: EMBO J. 2025 Dec 9;45(6):1957–79. doi: 10.1038/s44318-025-00651-6 (PMC12992588; doi:10.1038/s44318-025-00651-6)
Supplement: Supplementary file 3 — Source data Fig. 1 [file 44318_2025_651_MOESM3_ESM.zip › Figure 1/1B/b-galactosidase assay_ApcA.pdf]

arrest peptide	genotype	b-galactosidase activity (units)			
		rep1	rep2	rep3	means
apcA	WT	12.39	15.44	15.19	14.34
apcA	A103C	65.75	78.47	101.08	81.77
apcA	A103D	60.76	94.03	71.61	75.47
apcA	A103E	43.45	62.27	47.98	51.23
apcA	A103F	55.98	76.05	56.08	62.71
apcA	A103G	47.55	66.06	45.75	53.12
apcA	A103H	55.57	91.70	59.40	68.89
apcA	A103I	50.48	82.26	65.93	66.22
apcA	A103K	58.07	91.33	67.58	72.33
apcA	A103L	60.39	74.35	57.65	64.13
apcA	A103M	41.46	65.75	48.04	51.75
apcA	A103N	65.66	100.75	79.40	81.94
apcA	A103P	47.75	69.59	44.35	53.90
apcA	A103Q	56.02	91.39	70.04	72.48
apcA	A103R	55.97	97.81	68.39	74.06
apcA	A103S	39.63	67.01	51.52	52.72
apcA	A103T	50.75	81.28	54.42	62.15
apcA	A103V	58.27	91.51	72.18	73.99
apcA	A103W	56.61	92.47	59.16	69.41
apcA	A103Y	62.63	78.30	92.49	77.81
apcA	P104A	61.57	109.24	104.34	91.72
apcA	P104C	64.87	105.43	82.75	84.35
apcA	P104D	62.55	106.30	67.07	78.64
apcA	P104E	61.76	113.41	79.68	84.95
apcA	P104F	72.04	105.83	98.28	92.05
apcA	P104G	71.54	88.54	92.09	84.06
apcA	P104H	65.36	99.36	83.22	82.65
apcA	P104I	65.23	102.29	113.00	93.51
apcA	P104K	60.41	89.76	90.04	80.07
apcA	P104L	63.19	87.87	68.18	73.08
apcA	P104M	59.53	82.19	71.95	71.22
apcA	P104N	69.88	98.40	78.94	82.41
apcA	P104Q	64.44	82.72	75.75	74.31
apcA	P104R	64.66	99.59	82.52	82.25
apcA	P104S	40.33	83.17	69.78	64.43
apcA	P104T	60.12	89.03	74.49	74.54
apcA	P104V	67.29	98.34	74.27	79.97
apcA	P104W	66.03	86.44	68.23	73.57
apcA	P104Y	70.86	95.26	79.08	81.73
apcA	R102A	72.47	88.30	86.02	82.26
apcA	R102C	69.65	84.35	65.41	73.14
apcA	R102D	87.16	92.10	76.37	85.21
apcA	R102E	82.09	94.26	87.87	88.07
apcA	R102F	64.41	79.48	73.34	72.41
apcA	R102G	67.56	86.44	86.40	80.13
apcA	R102H	61.32	72.74	57.45	63.83
apcA	R102I	67.83	87.78	74.41	76.67
apcA	R102K	65.64	99.09	75.65	80.12
apcA	R102L	62.97	95.71	69.88	76.19
apcA	R102M	61.93	89.90	71.77	74.53
apcA	R102N	59.07	80.28	74.79	71.38
apcA	R102P	77.07	88.23	75.95	80.42
apcA	R102Q	62.02	72.45	56.34	63.60
apcA	R102S	65.86	90.62	78.49	78.32
apcA	R102T	58.15	89.34	82.54	76.68
apcA	R102V	72.40	94.08	75.97	80.82
apcA	R102W	61.63	77.86	71.85	70.45
apcA	R102Y	69.80	73.26	60.39	67.82
