# Supplementary material for: Evolutionary adaptation of bacterial proteomes to translation-impeding sequences
Source: EMBO J. 2025 Dec 9;45(6):1957–79. doi: 10.1038/s44318-025-00651-6 (PMC12992588; doi:10.1038/s44318-025-00651-6)
Supplement: Supplementary file 3 — Source data Fig. 1 [file 44318_2025_651_MOESM3_ESM.zip › Figure 1/1E/b-galactosidase assay_ApdP_Ec.pdf]

arrest peptide	genotype	b-galactosidase activity (units)			
		rep1	rep2	rep3	means
apdP	WT	163.52	167.01	185.30	171.94
apdP	R131A	3349.03	3341.76	3372.89	3354.56
apdP	R131D	2540.74	2775.80	2877.66	2731.40
apdP	R131E	2756.45	2893.54	2868.48	2839.49
apdP	R131G	2502.25	2582.27	2722.63	2602.38
apdP	R131I	2648.61	2661.80	2703.31	2671.24
apdP	R131K	2438.20	2525.25	2864.26	2609.24
apdP	R131L	2300.62	2367.63	2148.67	2272.30
apdP	R131M	2567.83	3091.14	2643.25	2767.41
apdP	R131S	2768.15	2937.00	2818.90	2841.35
apdP	R131V	2580.02	2741.55	2722.48	2681.35
apdP	R131W	2582.29	3267.95	2711.75	2854.00
apdP	R131C	2329.53	2449.38	2369.16	2382.69
apdP	R131H	2508.44	2502.46	2552.38	2521.09
apdP	R131N	1560.74	1236.50	1674.44	1490.56
apdP	R131P	2473.08	2424.87	2584.09	2494.02
apdP	R131Q	2667.57	2674.50	3044.13	2795.40
apdP	R131Y	2739.78	2764.29	2724.24	2742.77
apdP	R131F	2717.69	2877.89	2820.95	2805.51
apdP	R131T	2817.10	2949.12	2490.77	2752.33
apdP	WT	154.50	148.63	152.41	151.85
apdP	A132S	1193.10	1006.35	1065.56	1088.34
apdP	A132G	23.16	20.74	20.16	21.35
apdP	A132V	2538.47	2503.45	2581.77	2541.23
apdP	A132W	2218.92	2267.49	2219.52	2235.31
apdP	A132D	744.48	694.12	652.18	696.93
apdP	A132E	1543.90	1546.19	1556.31	1548.80
apdP	A132F	3275.88	3255.59	3003.44	3178.30
apdP	A132K	3294.53	3352.34	3130.66	3259.18
apdP	A132L	2389.03	2356.22	2385.15	2376.80
apdP	A132Q	2287.35	2493.52	2502.72	2427.86
apdP	A132T	2491.47	2380.72	2460.05	2444.08
apdP	A132C	2177.99	2426.80	2399.73	2334.84
apdP	A132M	2347.89	2569.58	2410.50	2442.66
apdP	A132N	2269.12	2292.77	2365.33	2309.08
apdP	A132H	2217.38	2353.54	2267.47	2279.47
apdP	A132I	2254.24	2250.81	2426.65	2310.57
apdP	A132P	483.96	503.37	485.82	491.05
apdP	A132R	2527.16	2390.69	2546.87	2488.24
apdP	A132Y	1883.15	2026.49	2030.04	1979.89
apdP	WT	178.83	150.81	153.71	161.12
apdP	P133A	2283.58	2348.28	2269.26	2300.37
apdP	P133G	338.35	334.84	326.23	333.14
apdP	P133H	2470.11	2260.40	2342.61	2357.71
apdP	P133I	3022.17	3121.51	3128.64	3090.78
apdP	P133L	2622.54	2668.18	2525.03	2605.25
apdP	P133M	2380.64	2376.89	2462.63	2406.72
apdP	P133S	2039.85	1965.99	2112.57	2039.47
apdP	P133V	2489.47	2727.34	2674.07	2630.29
apdP	P133Y	2464.25	2619.56	2395.05	2492.95
apdP	P133F	2425.48	2439.21	2546.33	2470.34
apdP	P133Q	2577.74	2519.63	2548.69	2548.69
apdP	P133W	2324.21	2532.39	2619.34	2491.98
apdP	P133C	2314.66	2597.00	2392.06	2434.57
apdP	P133D	2071.01	2231.59	2135.67	2146.09
apdP	P133E	3469.87	3513.99	3301.13	3428.33
apdP	P133N	2630.31	2464.12	2565.83	2553.42
apdP	P133R	2690.57	2568.06	2724.51	2661.04
apdP	P133T	3172.74	3256.79	3371.76	3267.10
apdP	P133K	2555.79	2631.53	2852.90	2680.07
