# Supplementary material for: Evolutionary adaptation of bacterial proteomes to translation-impeding sequences
Source: EMBO J. 2025 Dec 9;45(6):1957–79. doi: 10.1038/s44318-025-00651-6 (PMC12992588; doi:10.1038/s44318-025-00651-6)
Supplement: Supplementary file 3 — Source data Fig. 1 [file 44318_2025_651_MOESM3_ESM.zip › Figure 1/1D/b-galactosidase assay_ApdP_Bs.pdf]

arrest peptide	genotype	b-galactosidase activity (units)			
		rep1	rep2	rep3	means
apdP	WT	2.3	1.3	2.2	1.9
apdP	R131A	36.9	20.9	46.0	34.6
apdP	R131C	34.9	20.4	44.8	33.4
apdP	R131D	33.8	22.5	47.7	34.6
apdP	R131E	33.7	19.3	43.3	32.1
apdP	R131F	34.2	21.3	40.8	32.1
apdP	R131G	36.2	24.2	42.3	34.3
apdP	R131H	35.5	23.4	41.4	33.4
apdP	R131I	38.0	23.2	41.6	34.2
apdP	R131K	37.9	25.2	46.1	36.4
apdP	R131L	38.8	25.7	45.4	36.6
apdP	R131M	38.6	23.8	43.8	35.4
apdP	R131N	30.6	19.1	39.5	29.7
apdP	R131P	38.4	22.6	45.4	35.5
apdP	R131Q	37.5	24.9	42.6	35.0
apdP	R131S	41.0	26.7	44.2	37.3
apdP	R131T	34.7	19.8	46.7	33.7
apdP	R131V	37.9	20.4	41.8	33.4
apdP	R131W	38.6	24.7	43.1	35.5
apdP	R131Y	35.2	22.4	43.4	33.7
apdP	A132C	28.7	17.7	36.8	27.7
apdP	A132D	13.7	7.1	13.1	11.3
apdP	A132E	23.8	11.2	22.5	19.1
apdP	A132F	34.2	19.9	39.0	31.0
apdP	A132G	1.2	1.4	0.1	0.9
apdP	A132H	31.5	17.3	35.4	28.1
apdP	A132I	34.5	21.1	40.8	32.1
apdP	A132K	34.9	21.3	41.3	32.5
apdP	A132L	29.0	16.3	33.6	26.3
apdP	A132M	30.7	25.2	72.7	42.8
apdP	A132N	41.7	20.8	43.2	35.2
apdP	A132P	2.4	1.5	3.3	2.4
apdP	A132Q	40.1	21.0	42.3	34.4
apdP	A132R	36.1	18.0	37.0	30.4
apdP	A132S	17.4	8.9	21.4	15.9
apdP	A132T	36.3	25.0	39.1	33.5
apdP	A132V	17.2	9.3	17.8	14.8
apdP	A132W	38.3	21.0	38.4	32.6
apdP	A132Y	34.5	17.8	37.0	29.7
apdP	P133A	39.3	22.3	38.2	33.3
apdP	P133C	38.8	19.2	44.9	34.3
apdP	P133D	27.1	15.2	49.4	30.6
apdP	P133E	37.8	24.7	40.4	34.3
apdP	P133F	40.1	21.9	40.0	34.0
apdP	P133G	7.4	4.2	7.7	6.4
apdP	P133H	41.0	21.0	41.5	34.5
apdP	P133I	44.8	22.5	46.7	38.0
apdP	P133K	35.7	19.8	85.0	46.8
apdP	P133L	40.1	29.5	41.1	36.9
apdP	P133M	39.9	23.0	44.4	35.8
apdP	P133N	35.1	20.6	86.3	47.4
apdP	P133Q	43.7	23.9	44.1	37.3
apdP	P133R	34.9	24.2	40.3	33.2
apdP	P133S	37.0	16.7	67.6	40.4
apdP	P133T	34.3	25.7	43.2	34.4
apdP	P133V	44.8	27.5	44.3	38.9
apdP	P133W	44.5	27.1	44.1	38.6
apdP	P133Y	41.4	21.9	40.9	34.7
