# Supplementary material for: Evolutionary adaptation of bacterial proteomes to translation-impeding sequences
Source: EMBO J. 2025 Dec 9;45(6):1957–79. doi: 10.1038/s44318-025-00651-6 (PMC12992588; doi:10.1038/s44318-025-00651-6)
Supplement: Supplementary file 3 — Source data Fig. 1 [file 44318_2025_651_MOESM3_ESM.zip › Figure 1/1C/b-galactosidase assay_ApdA.pdf]

arrest peptide	genotype	b-galactosidase activity (units)			
		rep1	rep2	rep3	means
apdA	WT	4.1	2.5	2.5	3.0
apdA	R120A	32.7	23.7	34.0	30.1
apdA	R120C	39.9	25.6	41.5	35.7
apdA	R120D	29.5	36.1	35.5	33.7
apdA	R120E	29.6	23.1	37.1	29.9
apdA	R120F	28.4	25.7	30.4	28.2
apdA	R120G	43.4	30.7	37.6	37.2
apdA	R120H	33.4	24.6	30.5	29.5
apdA	R120I	32.3	23.9	30.4	28.9
apdA	R120K	36.5	24.5	42.8	34.6
apdA	R120L	32.2	17.0	36.9	28.7
apdA	R120M	34.6	39.1	41.3	38.3
apdA	R120N	33.4	20.5	32.9	28.9
apdA	R120P	22.2	17.7	22.1	20.7
apdA	R120Q	34.6	26.3	36.9	32.6
apdA	R120S	28.5	20.9	31.0	26.8
apdA	R120T	32.2	32.4	50.7	38.4
apdA	R120V	34.2	24.2	38.2	32.2
apdA	R120W	30.7	16.9	32.4	26.7
apdA	R120Y	28.2	25.8	29.2	27.7
apdA	A121C	31.0	18.2	31.2	26.8
apdA	A121D	12.8	6.1	11.2	10.0
apdA	A121E	35.4	31.2	36.7	34.4
apdA	A121F	28.4	16.3	26.9	23.9
apdA	A121G	2.9	2.0	2.4	2.4
apdA	A121H	41.7	18.2	34.1	31.3
apdA	A121I	31.2	12.4	25.7	23.1
apdA	A121K	41.1	16.7	35.7	31.2
apdA	A121L	38.7	23.5	42.7	35.0
apdA	A121M	39.2	32.5	41.7	37.8
apdA	A121N	36.8	20.0	30.0	28.9
apdA	A121P	13.7	8.4	16.4	12.9
apdA	A121Q	49.9	23.5	43.0	38.8
apdA	A121R	74.1	44.9	71.9	63.7
apdA	A121S	11.2	5.2	10.7	9.0
apdA	A121T	32.8	14.7	26.4	24.6
apdA	A121V	23.6	22.0	33.9	26.5
apdA	A121W	33.9	31.9	41.5	35.8
apdA	A121Y	41.3	24.8	44.5	36.9
apdA	P122A	62.5	22.7	56.4	47.2
apdA	P122C	70.6	37.0	56.7	54.8
apdA	P122D	58.5	26.8	49.1	44.8
apdA	P122E	61.3	29.6	51.3	47.4
apdA	P122F	54.4	19.7	41.3	38.5
apdA	P122G	27.5	19.7	35.0	27.4
apdA	P122H	45.1	40.3	56.4	47.3
apdA	P122I	56.4	25.0	49.1	43.5
apdA	P122K	61.0	23.9	53.1	46.0
apdA	P122L	77.4	30.5	61.6	56.5
apdA	P122M	77.9	34.4	63.5	58.6
apdA	P122N	56.2	27.3	43.1	42.2
apdA	P122Q	50.8	34.6	55.7	47.0
apdA	P122R	65.8	57.1	65.5	62.8
apdA	P122S	44.3	29.6	47.6	40.5
apdA	P122T	59.4	30.7	64.2	51.4
apdA	P122V	54.3	30.0	57.5	47.3
apdA	P122W	51.0	27.9	59.8	46.2
apdA	P122Y	43.9	31.4	44.4	39.9
