# Supplementary material for: Evolutionary adaptation of bacterial proteomes to translation-impeding sequences
Source: EMBO J. 2025 Dec 9;45(6):1957–79. doi: 10.1038/s44318-025-00651-6 (PMC12992588; doi:10.1038/s44318-025-00651-6)
Supplement: Supplementary file 4 — Source data Fig. 2 [file 44318_2025_651_MOESM4_ESM.zip › Figure 2/2A/b-galactosidase assay_ApdP_Bs_RAPP motif.pdf]

arrest peptide	genotype	b-galactosidase activity (units)			
		rep1	rep2	rep3	means
ApdP	WT	1.6	2.5	1.4	1.9
ApdP	AAPP	27.9	29.0	26.7	27.9
ApdP	RGPP	0.6	0.2	0.5	0.4
ApdP	RAPG	36.2	34.8	36.0	35.7
ApdP	RAGP	5.5	5.7	5.5	5.6
ApdP	AAGP	32.8	31.7	27.3	30.6
