# Supplementary material for: Evolutionary adaptation of bacterial proteomes to translation-impeding sequences
Source: EMBO J. 2025 Dec 9;45(6):1957–79. doi: 10.1038/s44318-025-00651-6 (PMC12992588; doi:10.1038/s44318-025-00651-6)
Supplement: Supplementary file 4 — Source data Fig. 2 [file 44318_2025_651_MOESM4_ESM.zip › Figure 2/2C/b-galactosidase assay_ApcA_RAPP motif.pdf]

arrest peptide	genotype	b-galactosidase activity (units)			
		rep1	rep2	rep3	means
ApcA	WT	14.9	13.5	11.6	13.3
ApcA	AAPG	88.6	89.9	83.5	87.3
ApcA	RAPP	1.2	1.1	1.1	1.2
ApcA	RGPP	0.8	1.0	0.9	0.9
ApcA	AAPP	72.5	69.6	71.7	71.3
ApcA	RAGP	8.0	7.9	7.1	7.7
ApcA	AAGP	90.3	94.4	76.9	87.2
