# Supplementary material for: Evolutionary adaptation of bacterial proteomes to translation-impeding sequences
Source: EMBO J. 2025 Dec 9;45(6):1957–79. doi: 10.1038/s44318-025-00651-6 (PMC12992588; doi:10.1038/s44318-025-00651-6)
Supplement: Supplementary file 4 — Source data Fig. 2 [file 44318_2025_651_MOESM4_ESM.zip › Figure 2/2D/b-galactosidase assay_SecM_RAPP motif.pdf]

arrest peptide	genotype	b-galactosidase activity (units)			
		rep1	rep2	rep3	means
SecM	WT	43.5	41.1	39.4	41.3
SecM	AAGP	2467.9	2268.1	2547.9	2428.0
SecM	RAGA	2115.7	2388.6	2357.4	2287.2
SecM	RAPP	49.9	49.0	55.8	51.6
SecM	AAPP	2069.4	2058.2	2129.2	2085.6
SecM	RGPP	6.9	7.0	5.6	6.5
SecM	RAPG	2375.9	2477.2	2323.6	2392.2
