# Supplementary material for: Evolutionary adaptation of bacterial proteomes to translation-impeding sequences
Source: EMBO J. 2025 Dec 9;45(6):1957–79. doi: 10.1038/s44318-025-00651-6 (PMC12992588; doi:10.1038/s44318-025-00651-6)
Supplement: Supplementary file 4 — Source data Fig. 2 [file 44318_2025_651_MOESM4_ESM.zip › Figure 2/2J/b-galactosidase assay_ApcA_rplD.pdf]

arrest peptide	subgroup	genotype	b-galactosidase activity (units)			
			rep1	rep2	rep3	means
ApcA	_rplD+	WT	26.29	24.68	23.01	24.66
ApcA	_rplD+	AAPG	113.47	136.16	101.46	117.03
ApcA	_rplD+	RAPP	6.49	3.81	2.95	4.42
ApcA	_rplD+	RGPP	2.97	3.57	2.88	3.14
ApcA	_rplD+	RAGP	14.74	12.54	15.45	14.24
ApcA	d66-70	WT	90.57	96.04	95.11	93.91
ApcA	d66-70	AAPG	113.67	116.13	121.86	117.22
ApcA	d66-70	RAPP	4.17	4.15	4.29	4.20
ApcA	d66-70	RGPP	3.29	3.05	2.36	2.90
ApcA	d66-70	RAGP	23.32	25.20	27.38	25.30
