# Supplementary material for: Evolutionary adaptation of bacterial proteomes to translation-impeding sequences
Source: EMBO J. 2025 Dec 9;45(6):1957–79. doi: 10.1038/s44318-025-00651-6 (PMC12992588; doi:10.1038/s44318-025-00651-6)
Supplement: Supplementary file 4 — Source data Fig. 2 [file 44318_2025_651_MOESM4_ESM.zip › Figure 2/2K/b-galactosidase assay_SecM_rplV.pdf]

arrest peptide	subgroup	genotype	b-galactosidase activity (units)			
			rep1	rep2	rep3	means
SecM	rplV+	WT	30.45	31.68	31.70	31.27
SecM	rplV+	RAPP	31.96	26.48	29.81	29.41
SecM	rplV+	RGPP	5.63	5.18	5.77	5.53
SecM	dMKR	WT	95.70	76.04	97.38	89.70
SecM	dMKR	RAPP	56.20	54.21	55.07	55.16
SecM	dMKR	RGPP	7.50	8.17	4.34	6.67
