# Supplementary material for: Evolutionary adaptation of bacterial proteomes to translation-impeding sequences
Source: EMBO J. 2025 Dec 9;45(6):1957–79. doi: 10.1038/s44318-025-00651-6 (PMC12992588; doi:10.1038/s44318-025-00651-6)
Supplement: Supplementary file 4 — Source data Fig. 2 [file 44318_2025_651_MOESM4_ESM.zip › Figure 2/2B/b-galactosidase assay_ApdP_Ec_RAPP motif.pdf]

arrest peptide	genotype	b-galactosidase activity (units)			
		rep1	rep2	rep3	means
ApdP	WT	110.2	113.5	104.8	109.5
ApdP	AAPP	2485.8	2060.4	2493.8	2346.6
ApdP	RGPP	11.9	12.2	10.2	11.4
ApdP	RAPG	2375.1	2421.7	2467.5	2421.5
ApdP	RAGP	150.6	150.1	153.1	151.3
ApdP	AAGP	2503.1	2346.4	2444.2	2431.2
