# Supplementary figures and images for: Evolutionary adaptation of bacterial proteomes to translation-impeding sequences
Source: EMBO J. 2025 Dec 9;45(6):1957–79. doi: 10.1038/s44318-025-00651-6 (PMC12992588; doi:10.1038/s44318-025-00651-6)

Fig. 2G

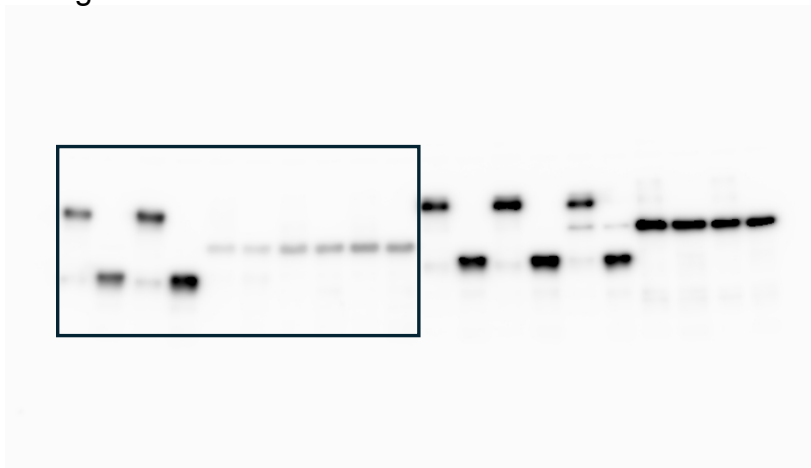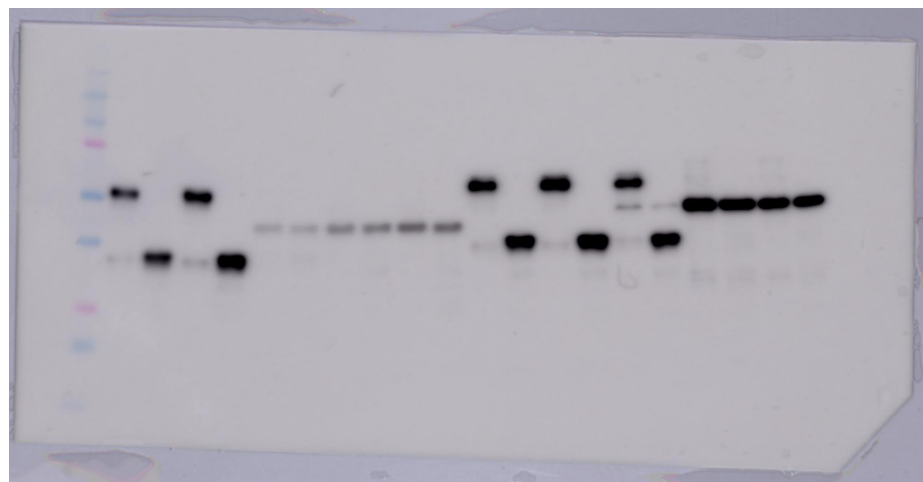

Overlay with molecular weight marker

Replicate

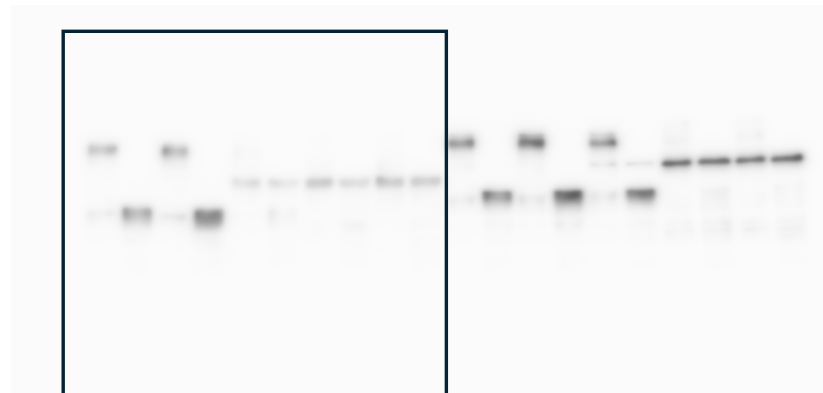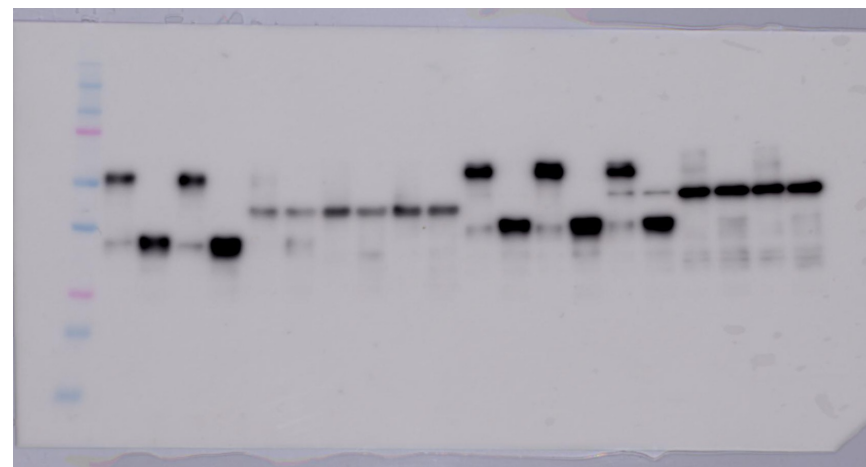

Overlay with molecular weight marker

Supplement: Supplementary file 4 — Source data Fig. 2 [file 44318_2025_651_MOESM4_ESM.zip › Figure 2/2G/Western blot_ApdP_Ec.pdf]

Fig. 2I

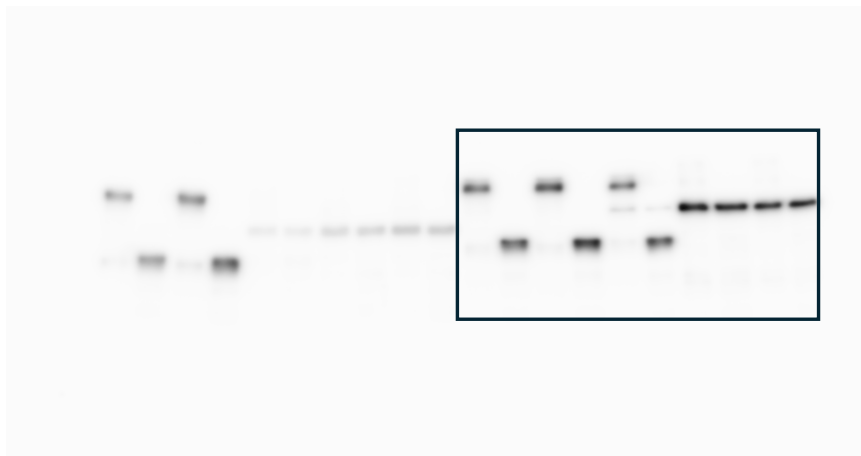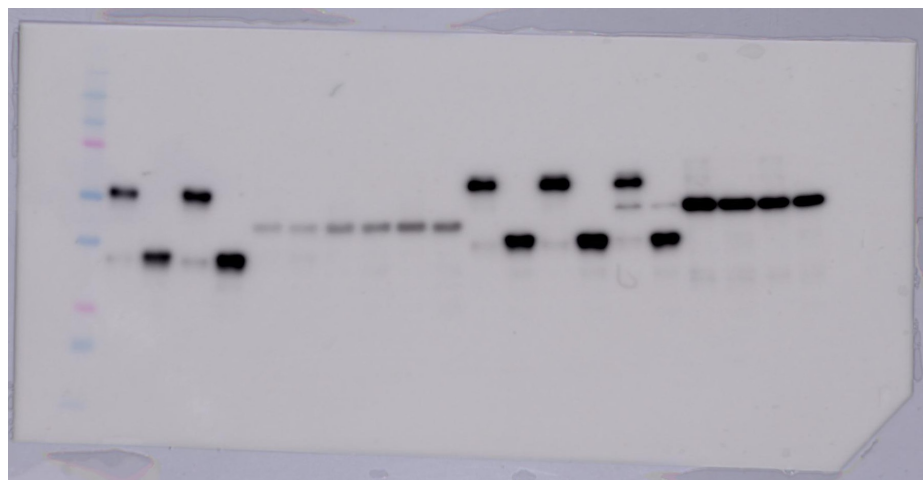

Overlay with molecular weight marker

Replicate

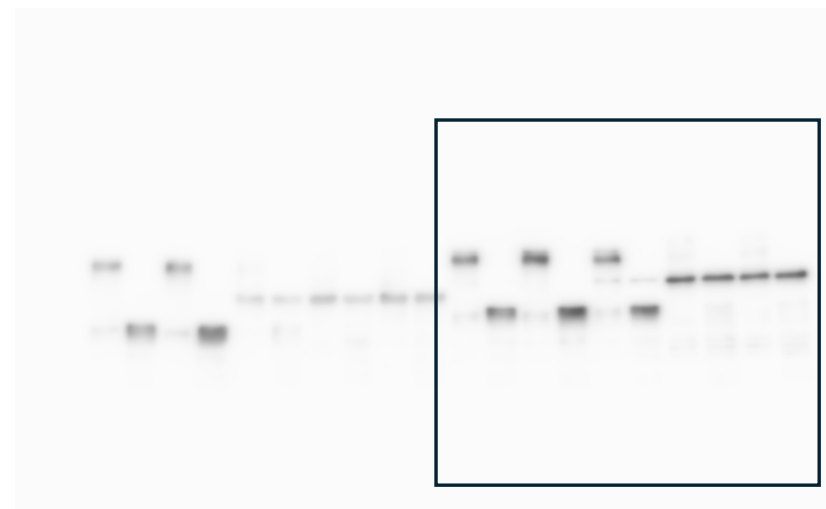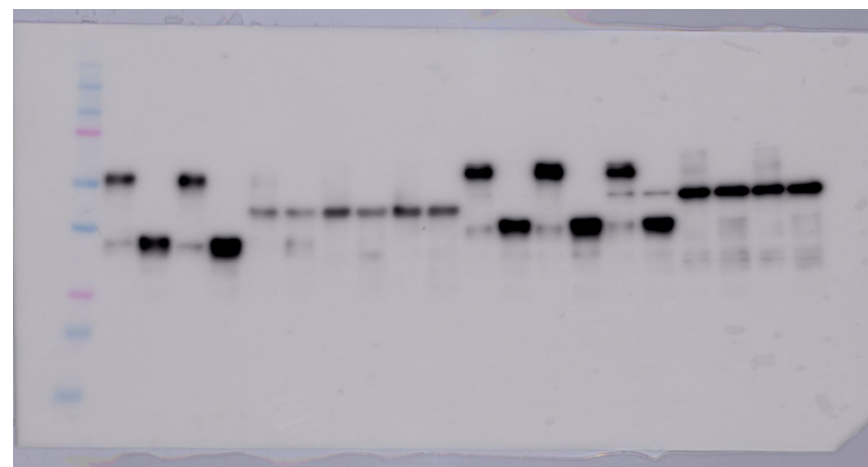

Overlay with molecular weight marker

Supplement: Supplementary file 4 — Source data Fig. 2 [file 44318_2025_651_MOESM4_ESM.zip › Figure 2/2I/Western blot_SecM.pdf]

Fig. 2H

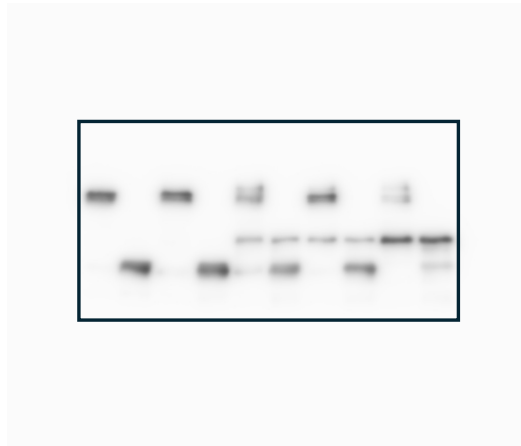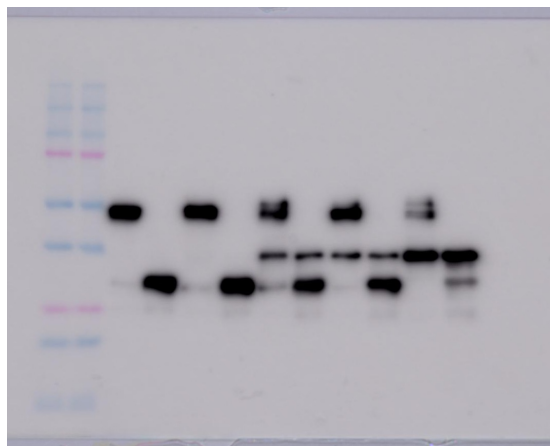

Overlay with molecular weight marker

Replicate

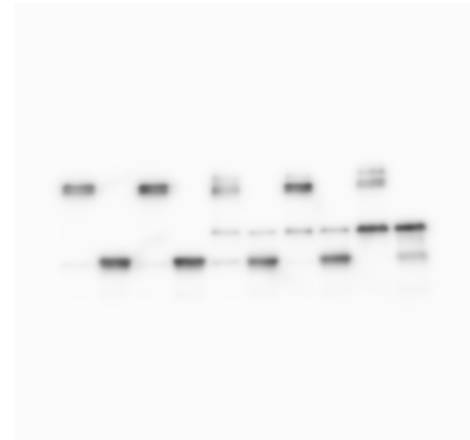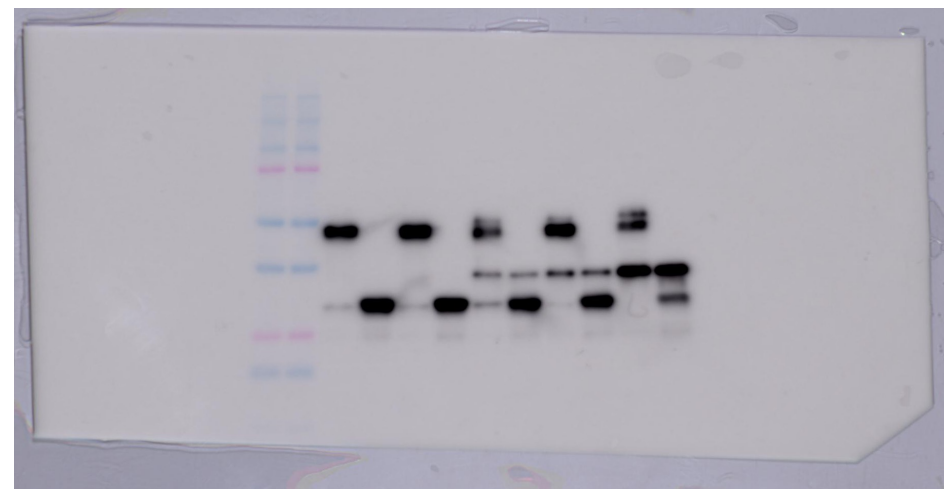

Overlay with molecular weight marker

Supplement: Supplementary file 4 — Source data Fig. 2 [file 44318_2025_651_MOESM4_ESM.zip › Figure 2/2H/Western blot_ApcA.pdf]

Fig. 2F

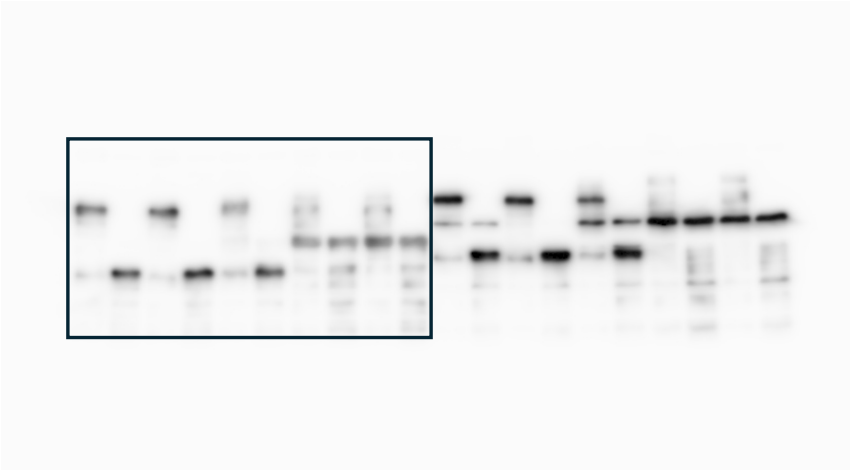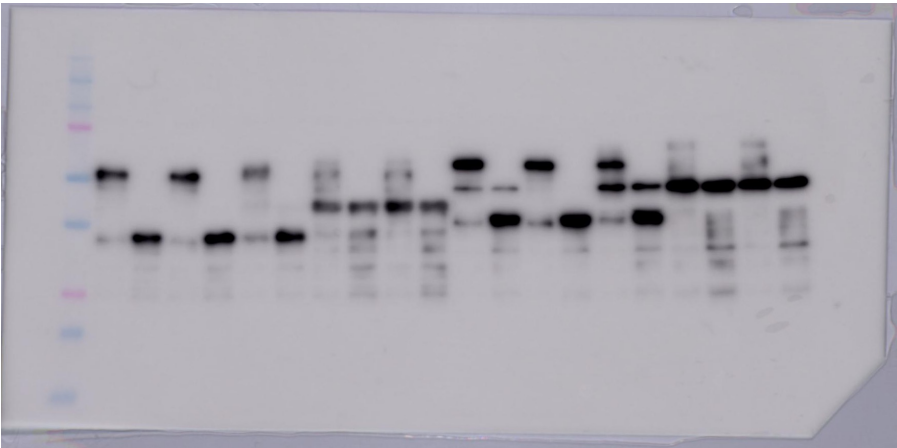

Overlay with molecular weight marker

replicate

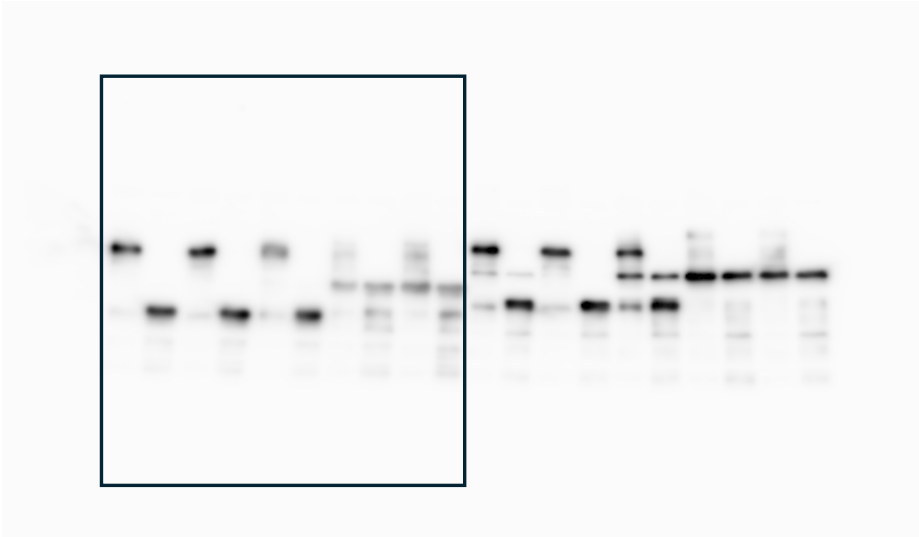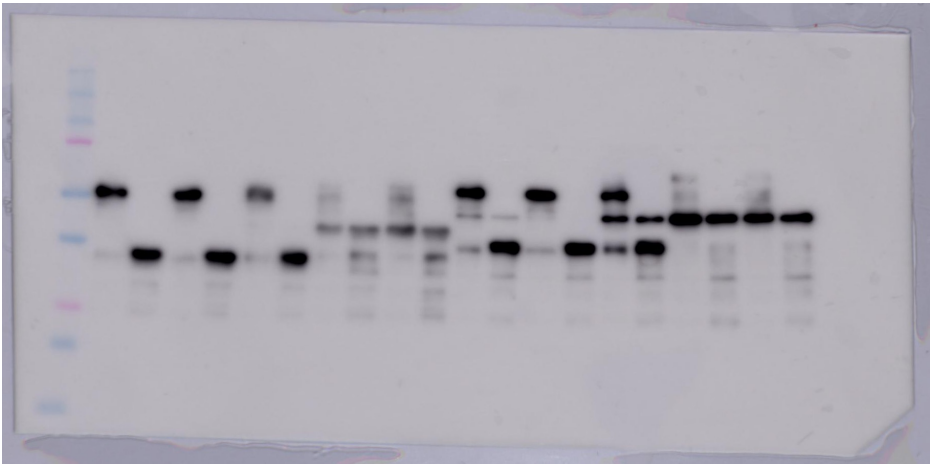

Overlay with molecular weight marker

Supplement: Supplementary file 4 — Source data Fig. 2 [file 44318_2025_651_MOESM4_ESM.zip › Figure 2/2F/Western blot_ApdP_Bs.pdf]

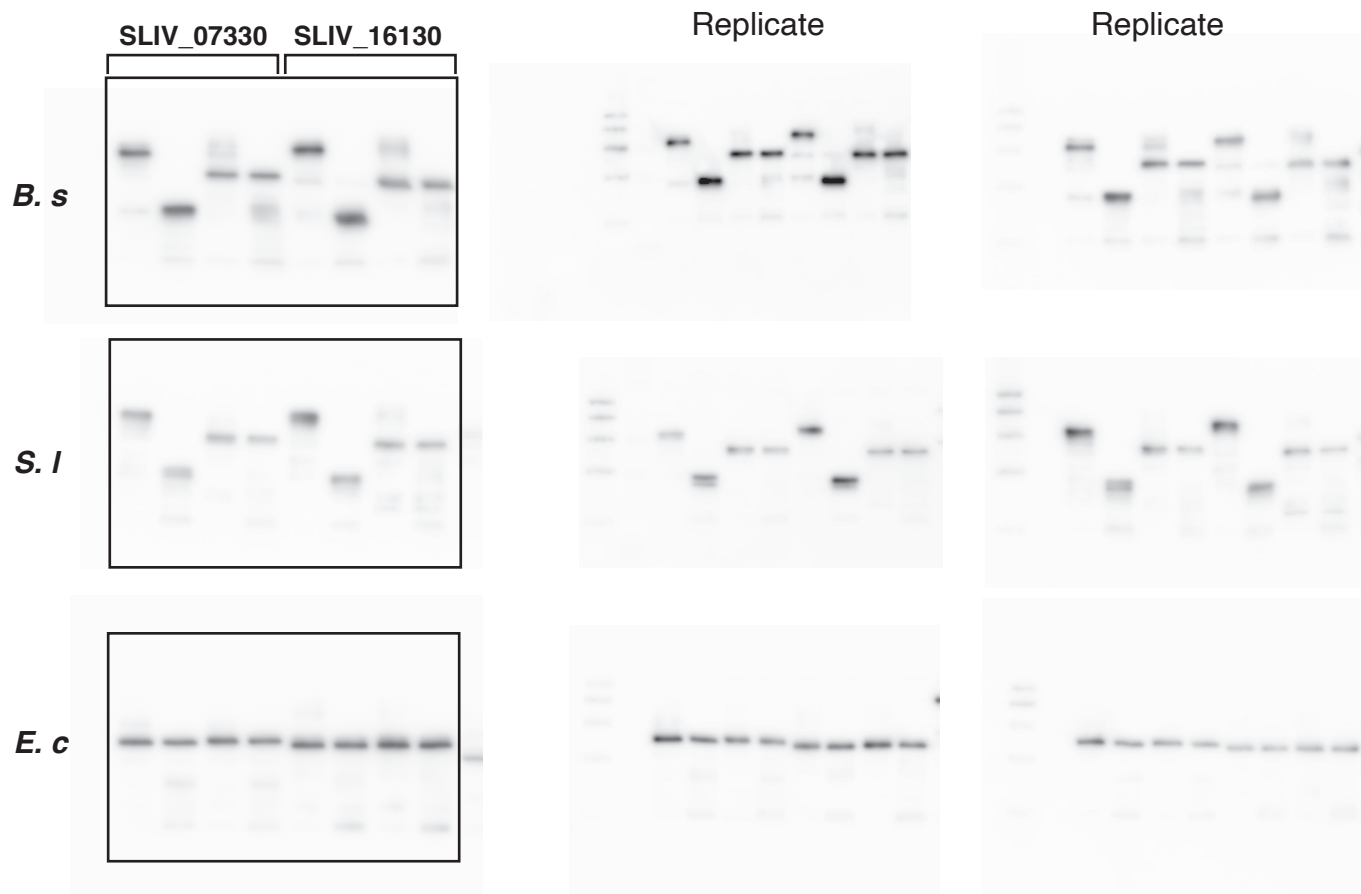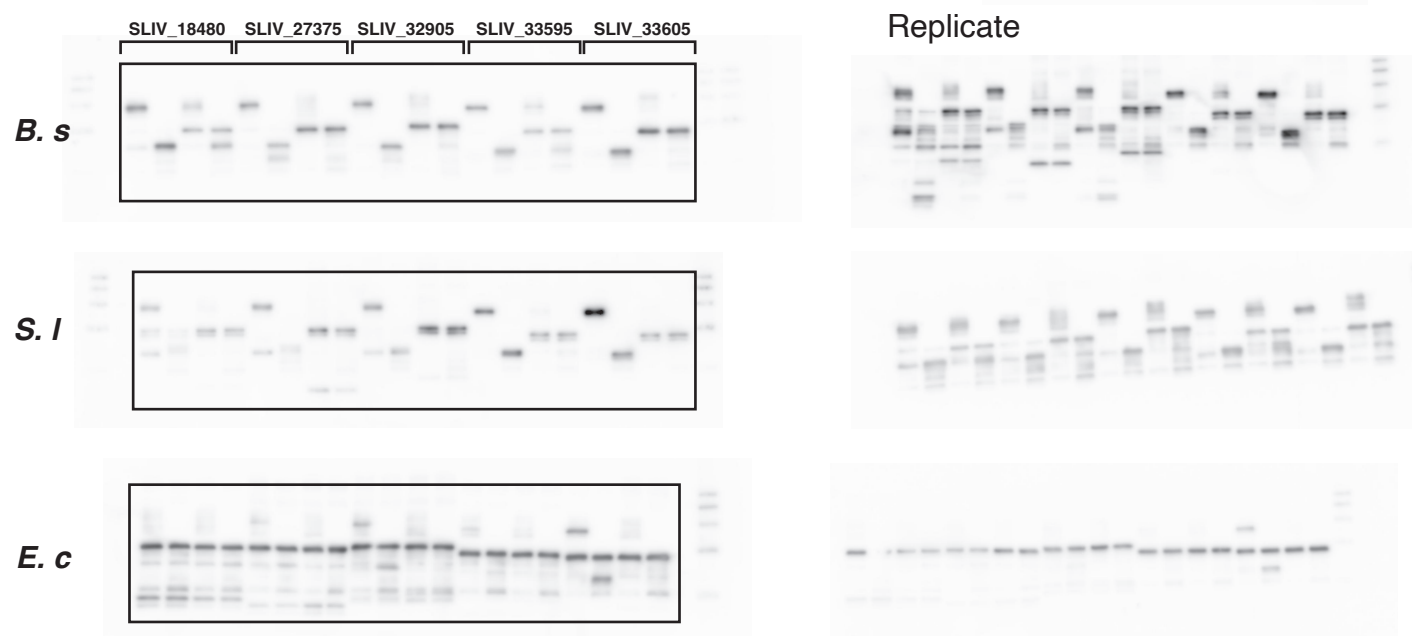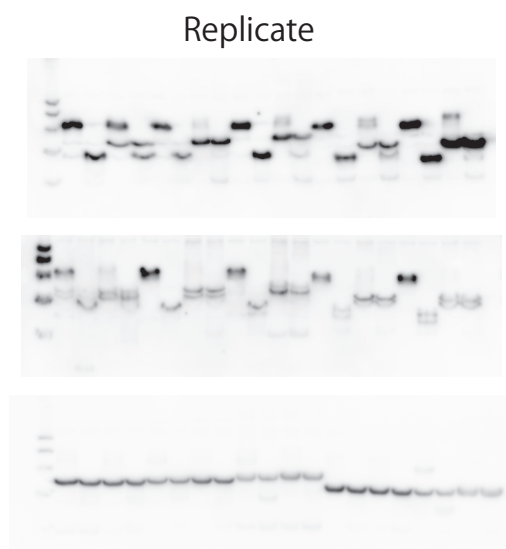

Supplement: Supplementary file 7 — Source data Fig. 5 [file 44318_2025_651_MOESM7_ESM.zip › Figure 5/5B/Western blot.pdf]

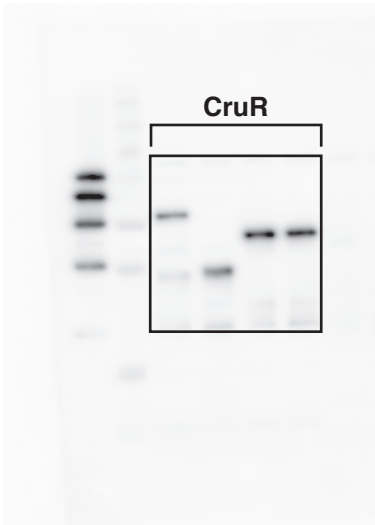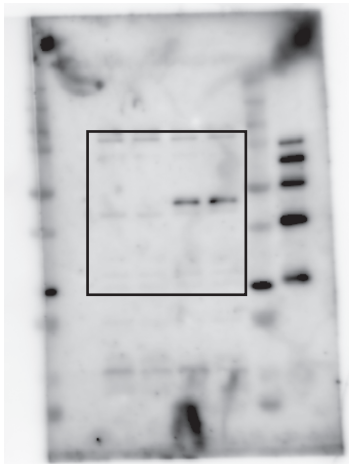

Replicate

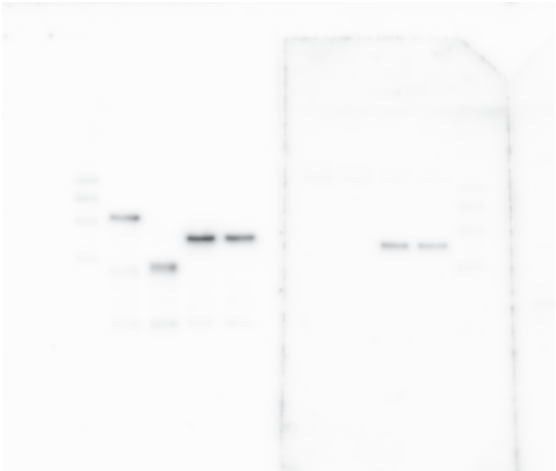

Supplement: Supplementary file 12 — Figure EV5 Source Data [file 44318_2025_651_MOESM12_ESM.zip › Source data_Fig EV5B.pdf]
