# Supplementary material for: Evolutionary adaptation of bacterial proteomes to translation-impeding sequences
Source: EMBO J. 2025 Dec 9;45(6):1957–79. doi: 10.1038/s44318-025-00651-6 (PMC12992588; doi:10.1038/s44318-025-00651-6)
Supplement: Supplementary file 7 — Source data Fig. 5 [file 44318_2025_651_MOESM7_ESM.zip › Figure 5/5C/b-galactosidase assay.pdf]

arrest peptide	genotype	b-galactosidase activity (units)			
		rep1	rep2	rep3	means
SLIV_07330	WT	6.18	5.18	4.91	5.42
SLIV_07330	R121A	72.26	49.45	73.45	65.05
SLIV_16130	WT	14.12	17.51	16.35	15.99
SLIV_16130	R105A	138.92	107.65	135.28	127.28
SLIV_08320	WT	10.82	10.06	10.24	10.37
SLIV_08320	R255A	6.05	6.90	6.39	6.45
SLIV_08320	P257,258A	20.04	17.79	15.12	17.65
SLIV_26065	WT	13.43	15.05	14.69	14.39
SLIV_18480	WT	1.82	1.38	2.14	1.78
SLIV_18480	R273A	18.84	19.41	19.15	19.13
SLIV_33595	WT	0.93	1.16	0.98	1.03
SLIV_33595	R225A	18.92	15.22	17.03	17.06
SLIV_33605	WT	1.55	1.70	1.65	1.63
SLIV_33605	R247A	9.26	8.41	8.75	8.81
SLIV_27375	WT	2.78	2.59	3.05	2.81
SLIV_27375	R273A	22.11	20.89	23.38	22.13
SLIV_32905	WT	1.55	2.03	1.57	1.72
SLIV_32905	R72A	6.77	8.13	8.57	7.82
