# Supplementary material for: Evolutionary adaptation of bacterial proteomes to translation-impeding sequences
Source: EMBO J. 2025 Dec 9;45(6):1957–79. doi: 10.1038/s44318-025-00651-6 (PMC12992588; doi:10.1038/s44318-025-00651-6)
Supplement: Supplementary file 8 — Figure EV1 Source Data [file 44318_2025_651_MOESM8_ESM.zip › Fig EV1/source_Appendix Fig. S1comp.pdf]

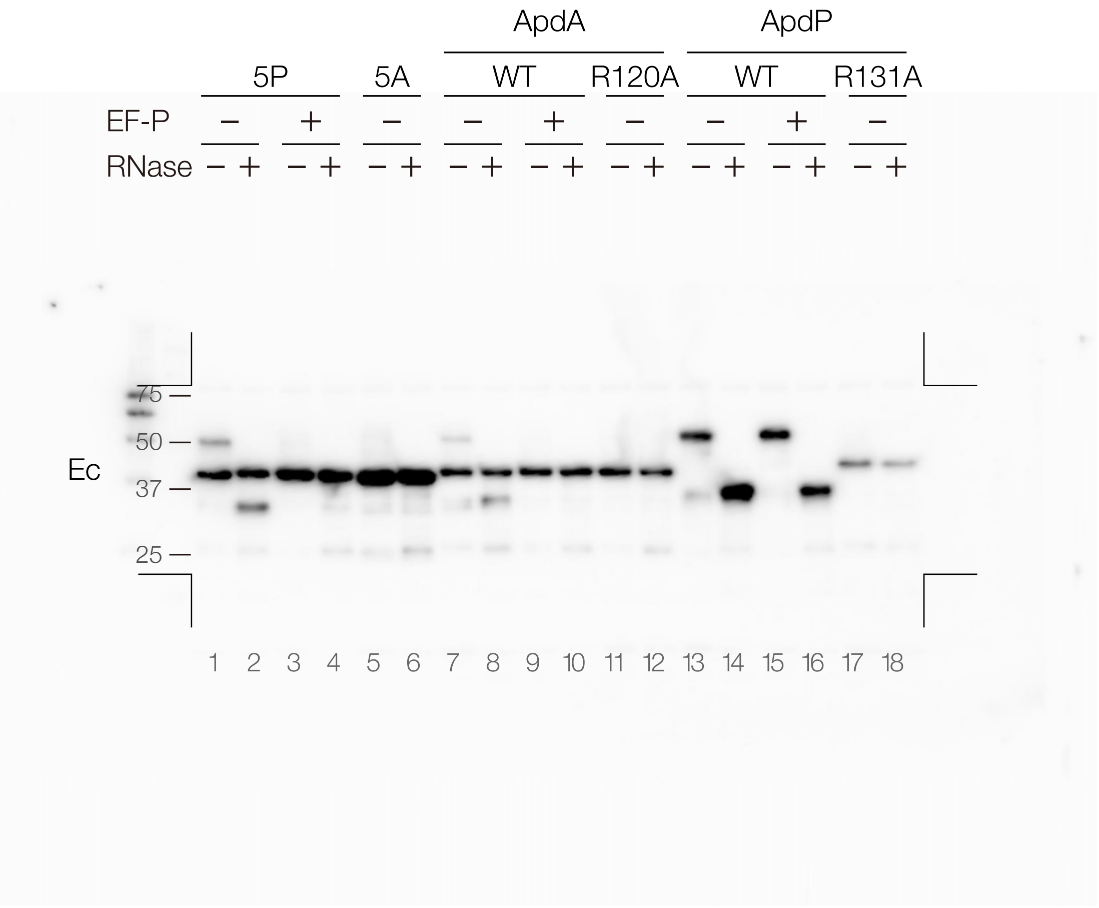

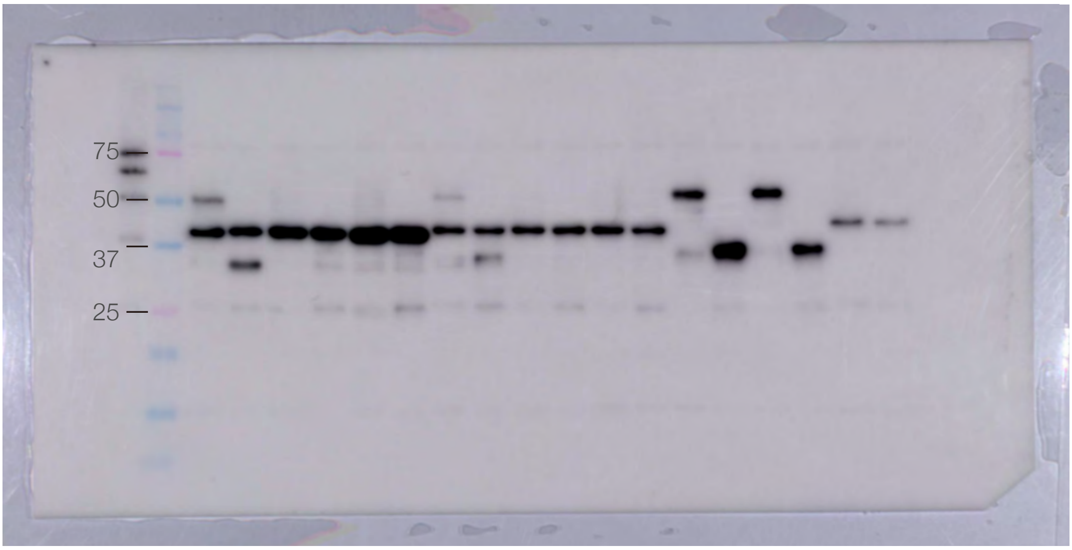

Figure S1, upper panel

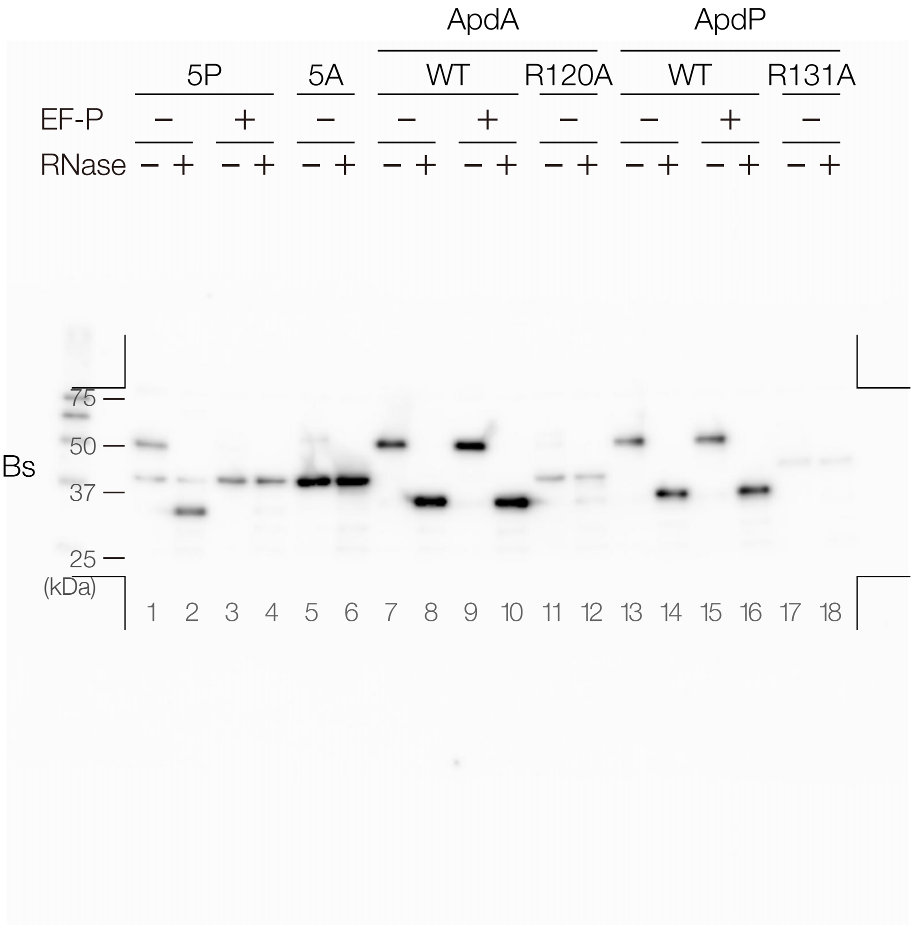

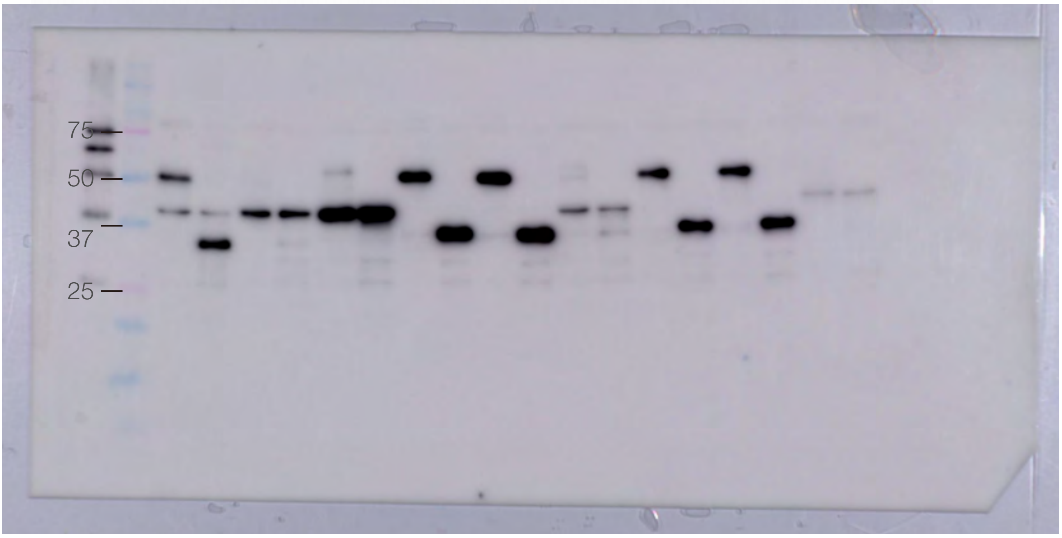

Figure S1, lower panel

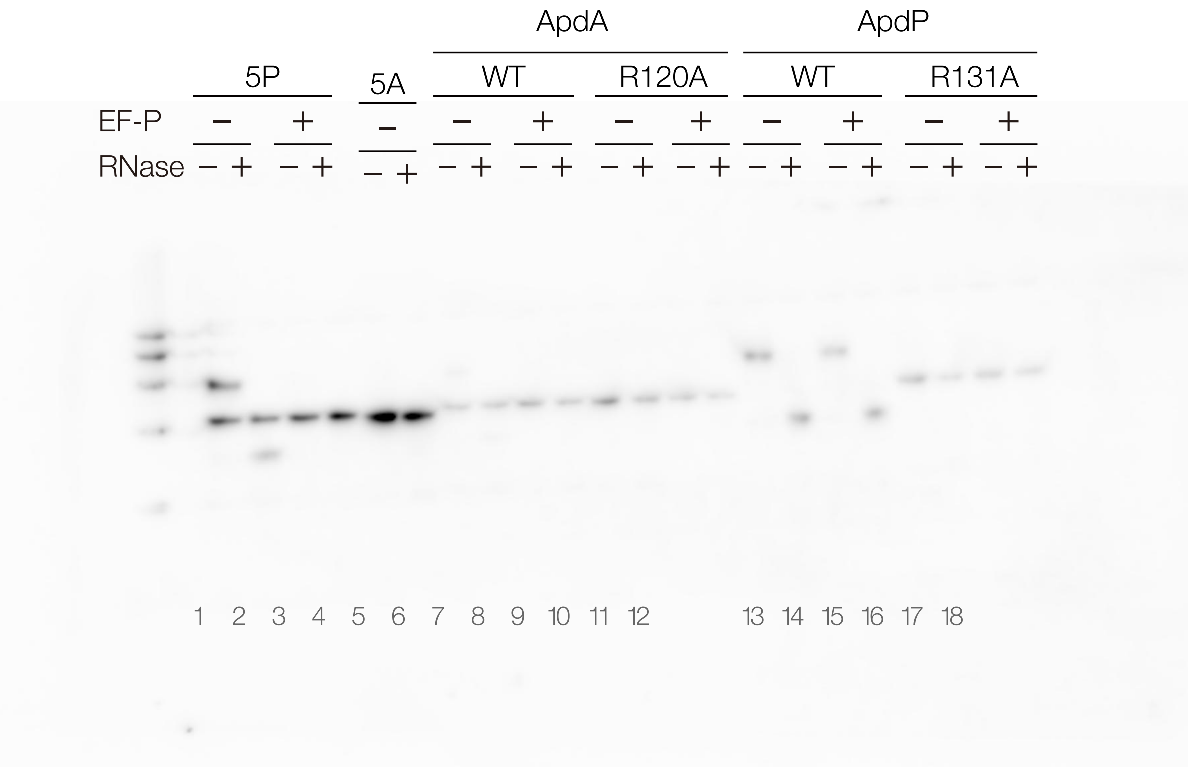

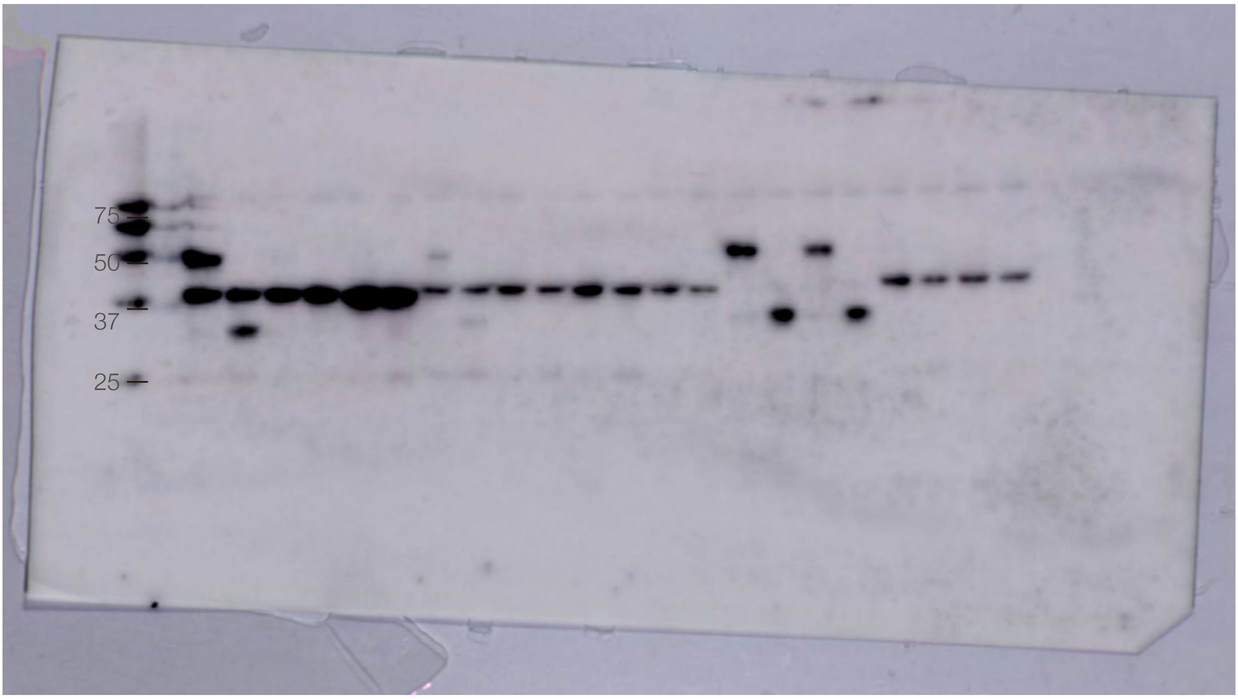

replicate data for
Figure S1, upper panel

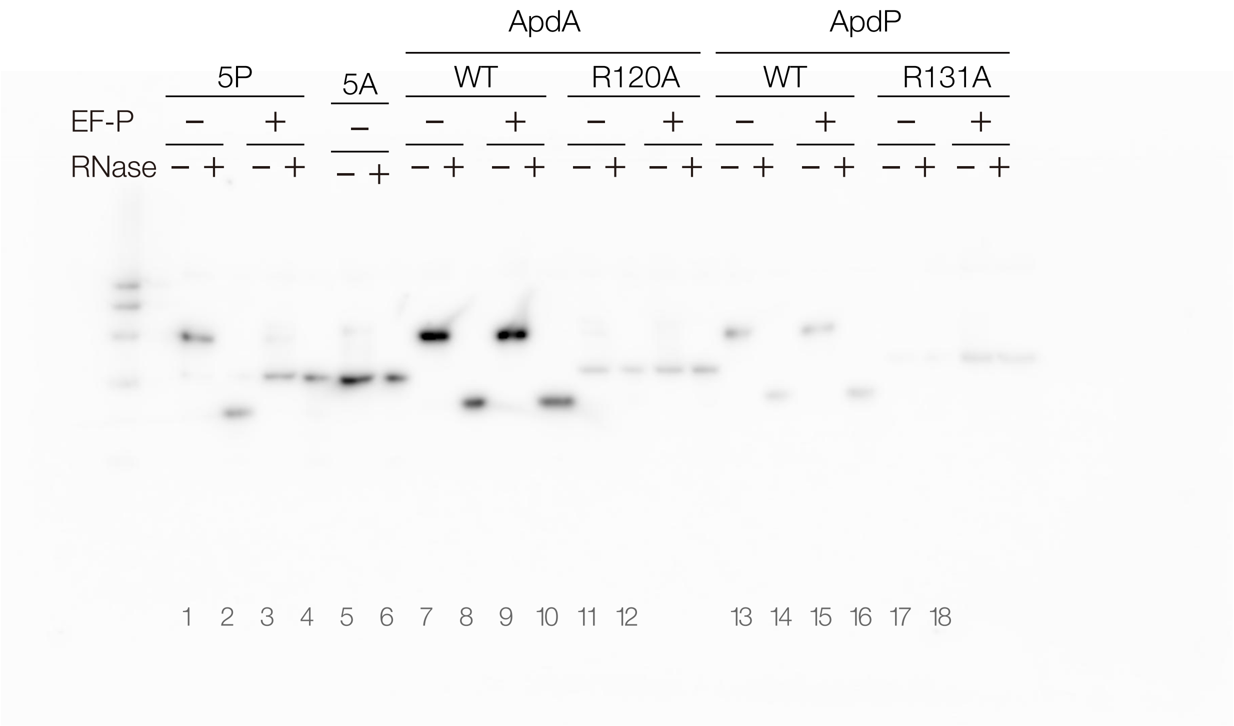

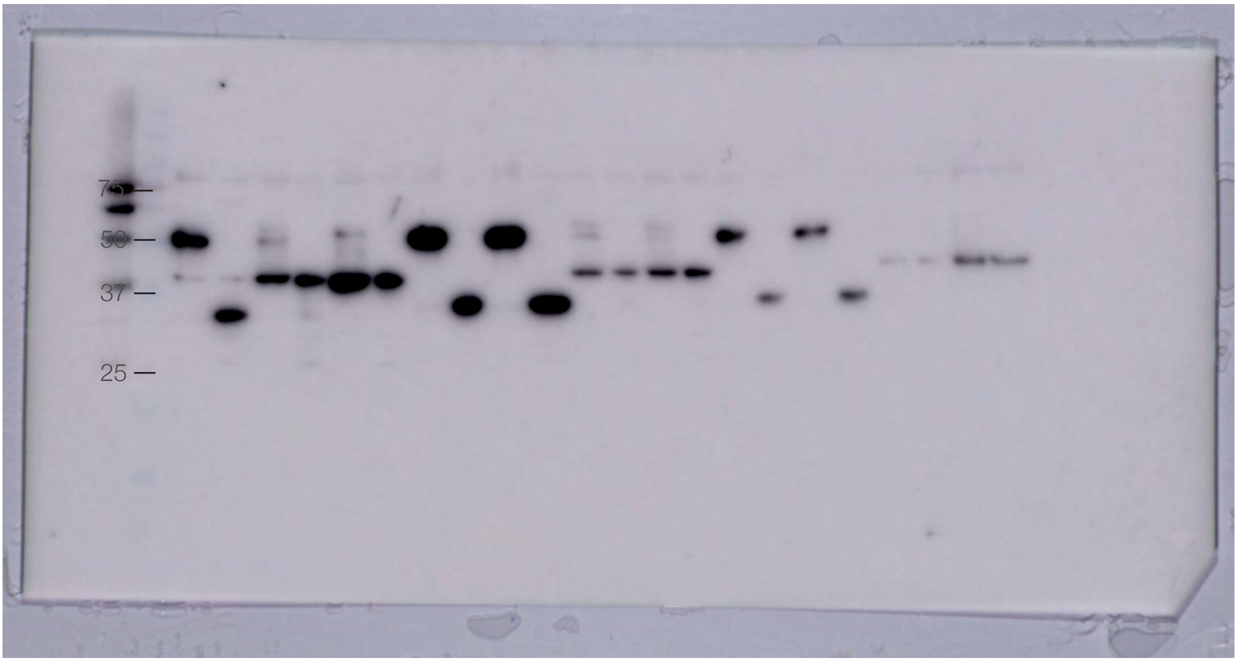

replicate data for
Figure S1, lower panel
