# Supplementary material for: Evolutionary adaptation of bacterial proteomes to translation-impeding sequences
Source: EMBO J. 2025 Dec 9;45(6):1957–79. doi: 10.1038/s44318-025-00651-6 (PMC12992588; doi:10.1038/s44318-025-00651-6)
Supplement: Supplementary file 14 — Expanded View Figures [file 44318_2025_651_MOESM14_ESM.pdf]

Expanded View Figures

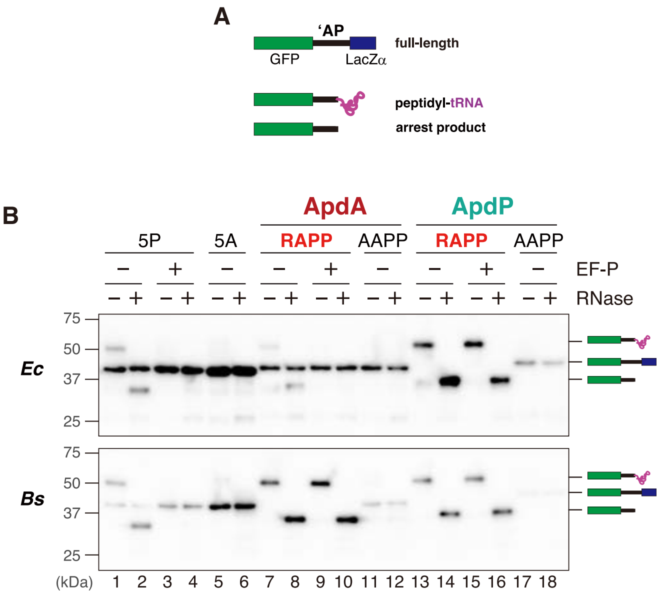

Figure EV1. Translation arrest of ApdA and ApdP is refractory to EF-P.

(**A**) A schematic representation of the reporter used for the in vitro translation arrest assay. A gene segment for a protein containing five consecutive prolines (5P), alanines (5A), or the arrest sequence of ApdA or ApdP was sandwich-fused with *gfp* and *lacZα*. The tRNA is shown in magenta. (**B**) Western blot analysis of the in vitro translation products. The reporter genes harboring the 5P, 5A sequences, or the arrest motifs of wild-type (RAPP) or mutant (AAPP) sequences indicated at the top were translated in the Ec (upper) or Bs (lower) PURE systems in the presence or absence of EF-P. The products were separated in neutral-pH gels and immunoblotted using anti-GFP antibody. Before the separation, a portion of the samples were treated with RNase A, to degrade the tRNA moiety. Molecular size standards are indicated on the left. Source data are available online for this figure.

 © The Author(s)

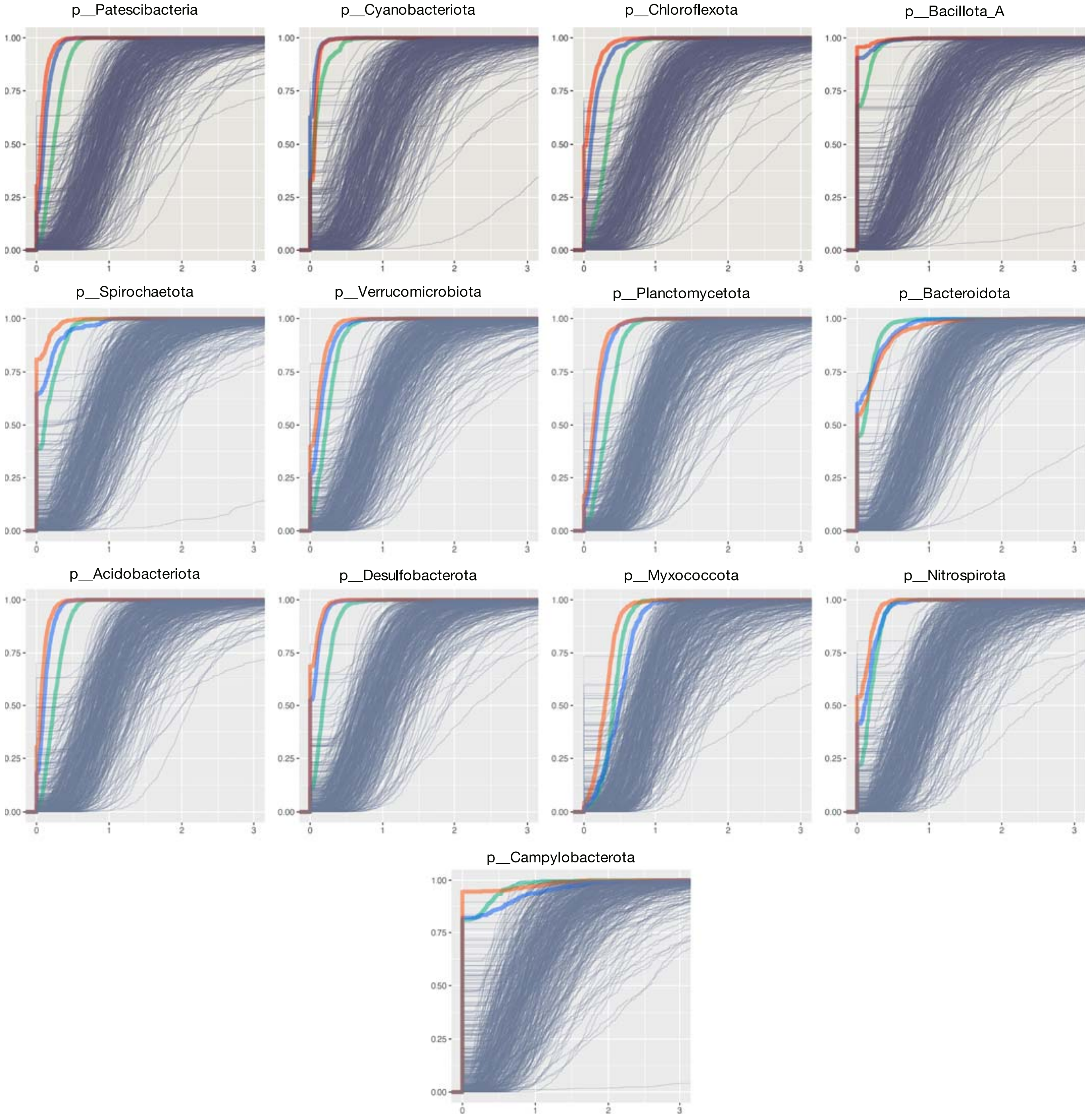

Figure EV2. RAPP-like sequences are underrepresented across bacterial phyla.

Empirical cumulative distribution functions (CDFs) of frequency bias for four-amino-acid sequences in phyla Patescibacteria ($n = 1000$), Cyanobacteriota ($n = 944$), Chloroflexota ($n = 1000$), Bacillota_A ($n = 1000$), Spirochaetota ($n = 606$), Verrucomicrobiota ($n = 865$), Planctomycetota ($n = 1000$), Bacteroidota ($n = 1000$), Acidobacteriota ($n = 758$), Myxococcota ($n = 425$), Nitrospirota ($n = 300$), Desulfobacterota ($n = 836$) and Campylobacterota ($n = 501$). Frequency bias values for the RGPP (red), RAPP (blue), RAGP (green) and randomly selected 500 patterns are plotted. Source data are available online for this figure.

A

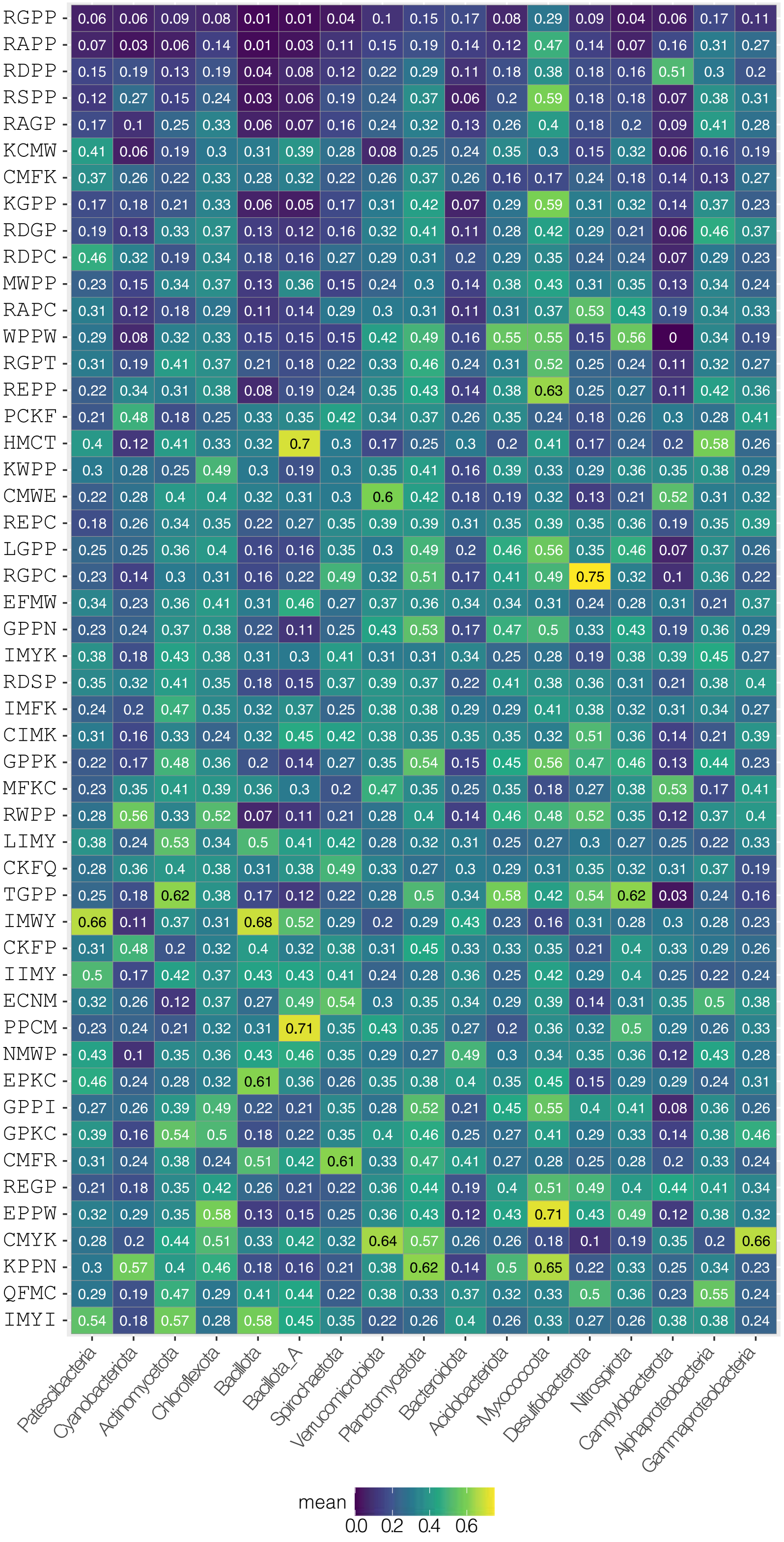

B

Gammaproteobacteria

pattern	mean	median	sd
RGPP	0.1052	0.0712	0.1261
CPCM	0.1114	0.0000	1.0029
HMCQ	0.1486	0.0000	0.8045
TGPP	0.1570	0.1047	0.2622
WCIM	0.1578	0.0000	0.9353
MWYD	0.1771	0.0000	0.6419
HMKC	0.1793	0.0000	0.9448
CPYM	0.1813	0.0000	0.7585
QCMC	0.1813	0.0000	1.4293
HGPP	0.1826	0.0000	0.2616
CMWY	0.1835	0.0000	1.4602
KCQM	0.1869	0.0000	0.7010
CKFQ	0.1888	0.0000	0.5779
KCMW	0.1888	0.0000	1.4757
KMCW	0.1930	0.0000	1.2579
WPPW	0.1931	0.0000	0.6024
PCMW	0.1957	0.0000	1.2009
KCMY	0.1968	0.0000	0.9455
RDPP	0.2002	0.1793	0.1937
GPPH	0.2015	0.1269	0.3362

C

Bacillota

pattern	mean	median	sd
RGPP	0.0107	0.0000	0.1538
RAPP	0.0144	0.0000	0.0984
RSPP	0.0302	0.0000	0.0937
RDPP	0.0373	0.0000	0.1595
RPPP	0.0429	0.0000	0.3477
KGPP	0.0619	0.0000	0.1411
RAGP	0.0632	0.0000	0.1923
RWPP	0.0743	0.0000	0.3424
REPP	0.0826	0.0000	0.1910
KPPP	0.0848	0.0000	0.1897
KPPW	0.0880	0.0000	0.3067
GPPW	0.0910	0.0000	0.3733
HPPP	0.0969	0.0000	0.3308
RAPC	0.1133	0.0000	0.4868
KDPP	0.1239	0.0000	0.2014
HGPP	0.1250	0.0000	0.4376
MWPP	0.1255	0.0000	0.6787
EPPW	0.1328	0.0000	0.5259
RDGP	0.1335	0.0000	0.2537
KPPD	0.1356	0.0000	0.2061

© The Author(s)

◀ **Figure EV3. Four-amino-acid sequences underrepresented in bacteria.**

(**A**) Heatmap showing the average frequency bias of four-amino-acid sequences with low frequency bias. The average frequency bias for each four-amino-acid sequence was calculated for each 15 phylum and two major class of the phylum Pseudomonadota. The top 50 patterns with the lowest mean of the frequency bias across all phyla are shown. (**B**, **C**) List of the top 20 underrepresented four-amino-acid sequences for class Gammaproteobacteria (**B**) and phylum Bacillota (**C**). Source data are available online for this figure.

© The Author(s)

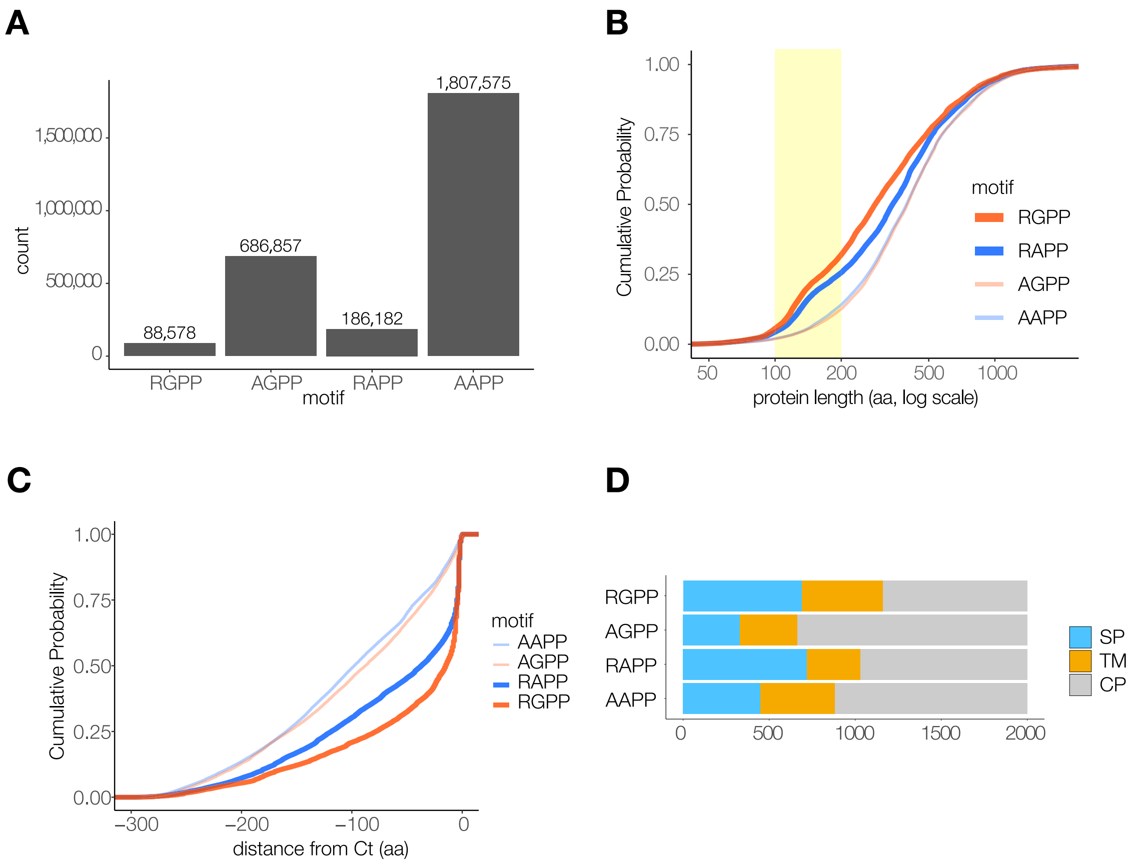

Figure EV4. RAPP-like motifs are enriched in the C-terminal regions of small proteins.

(**A**) Bar chart shows the count of proteins containing RGPP, AGPP, RAPP or AAPP motif within their sequences, found among representative proteomes of bacteria. (**B**) RAPP and RGPP motifs are enriched in relatively small proteins. ECDF plots show the length distribution of proteins containing RGPP (red), RAPP (blue), AGPP (pale red), and AAPP (pale blue). (**C**) ECDF plot shows the length distribution of distance from the C-terminus and RGPP (red, $n = 46{,}906$), RAPP (blue, $n = 78{,}810$), AGPP (pale red, $n = 216{,}471$), and AAPP (pale blue, $n = 600{,}525$). Analysis was performed on proteins with 300 or fewer amino acid residues. (**D**) RAPP and RGPP motifs are enriched in secretory proteins. Stacked bar plots display the proportions of predicted secretory proteins (SP: light blue), membrane proteins (TM: orange), and cytoplasmic proteins (CP: grey) containing RGPP, RAPP, AGPP, and AAPP motifs. Analysis was performed on 2000 randomly selected proteins with 300 or fewer amino acid residues using deepTMHMM. Source data are available online for this figure.

© The Author(s)

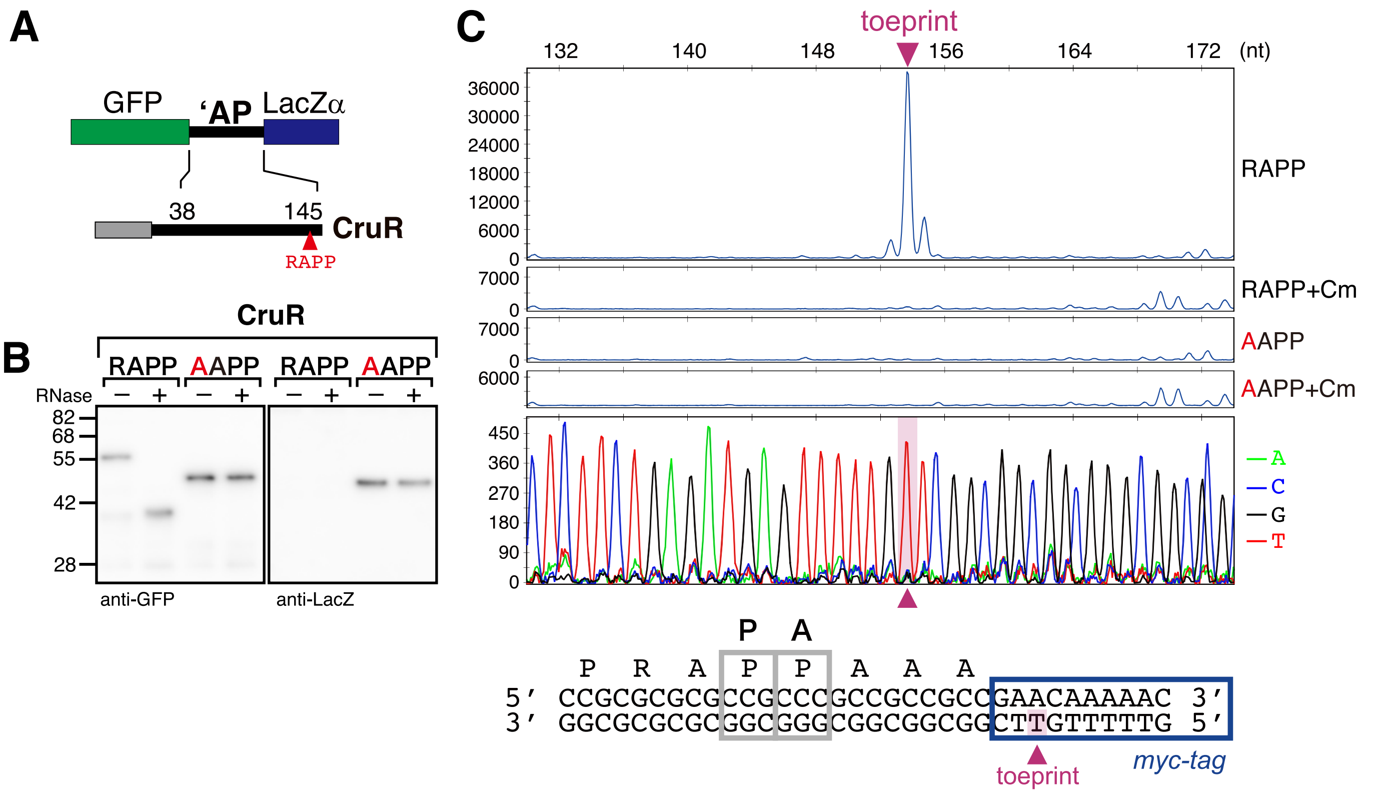

Figure EV5. ***Bordetella pertussis*** **CruR induces translation arrest in Ec PURE system.**

(**A**) A schematic representation of the reporter used for the in vitro translation arrest assay. A gene segment encoding the C-terminal soluble domain of CruR was fused in-frame between *gfp* and *lacZα*. (**B**) Western blot analysis of in vitro translation products. Since *B. pertussis* is a bacterium that belongs to the phylum Proteobacteria, we employed Ec PURE. The wild-type (RAPP) and R139A (AAPP) reporter genes, indicated at the top, were translated in the Ec PURE system in the presence of EF-P. The products were separated on neutral-pH gels and immunoblotted using anti-GFP and anti-LacZ antibodies. Prior to separation, a portion of the samples was treated with RNase A to degrade the tRNA moiety. Molecular size standards are indicated on the left. (**C**) Toeprint analysis of CruR. In vitro transcription-translation reaction mixtures containing templates encoding wild-type (RAPP) or R139A (AAPP) mutant reporters in the presence or absence of a translation inhibitor chloramphenicol (Cm) were subjected to fragment analysis on a SeqStudio Genetic Analyzer. Source data are available online for this figure.

© The Author(s)
